# Supplementary material for: High-throughput super-resolution single-particle trajectory analysis reconstructs organelle dynamics and membrane reorganization
Source: Cell Rep Methods. 2022 Aug 22;2(8):100277. doi: 10.1016/j.crmeth.2022.100277 (PMC9421586; doi:10.1016/j.crmeth.2022.100277)
Supplement: Document S1. Figure S1–S9 and Tables S1–S7 [file mmc1.pdf]

Cell Reports Methods, Volume *2*

Supplemental information

High-throughput super-resolution single-particle trajectory analysis reconstructs organelle dynamics and membrane reorganization

Pierre Parutto, Jennifer Heck, Meng Lu, Clemens Kaminski, Edward Avezov, Martin Heine, and David Holcman

Supplementary Figures and Tables

P. Parutto[1], J. Heck[2], M. Lu, C. Kaminski*
E. Avezov*, M. Heine[2*] and D. Holcman[13] *

July 23, 2022

[1] Group of Data Modeling and Computational Biology, IBENS, Ecole Normale Supérieure,75005 Paris, France. [2] Research Group Functional Neurobiology at the Institute of Developmental Biology and Neurobiology, Johannes Gutenberg University Mainz, Mainz, Germany and [3] DAMPT, University Of Cambridge, DAMPT and Churchill College CB30DS, United Kingdom. equally.

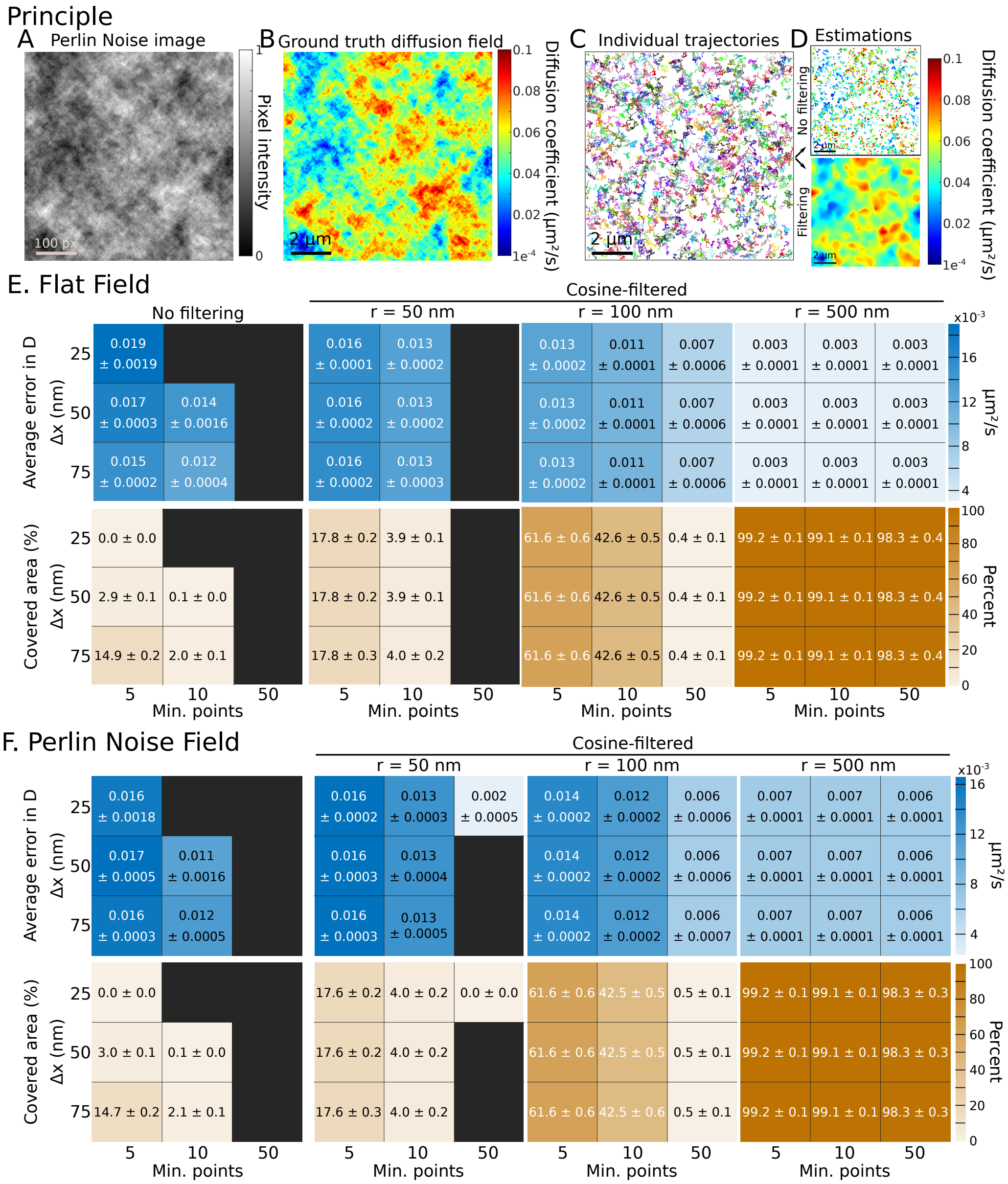

Figure S1: **Evaluation of diffusion field estimation**, related to STAR Methods. **A.** Perlin noise image used to generate a non-uniform diffusion field. **B.** The non-uniform diffusion field generated from A by setting a pixel size of 64.5 nm, $D_{min} = 1e^{-4} \mu m^2/s$ and $D_{max} = 0.1 \mu m^2/s$. **C.** Individual Brownian trajectories simulated from the diffusion field presented in B. **D.** Diffusion maps estimated from the trajectories presented in C without filtering (top, parameters $\Delta x = 100$ nm, $minPts = 5$) and with cosine-filtering (bottom, parameters $\Delta x = 50$ nm, $minPts = 10$, $r_{filt} = 500$ nm). **E.** Heat maps evaluating the quality of the reconstructed diffusion field for a uniform field with $D = 0.05 \mu m^2/s$ obtained over 100simulations. The top line (blue shade) reports the average error (see eq.1 of Data 1) between the reconstructed ground truth field while the bottom line (brown shade) shows the average percentage of the field area recovered. **F.** Same as E but for the non-uniform field presented in B. Black squares correspond to conditions where not enough data could be recovered.

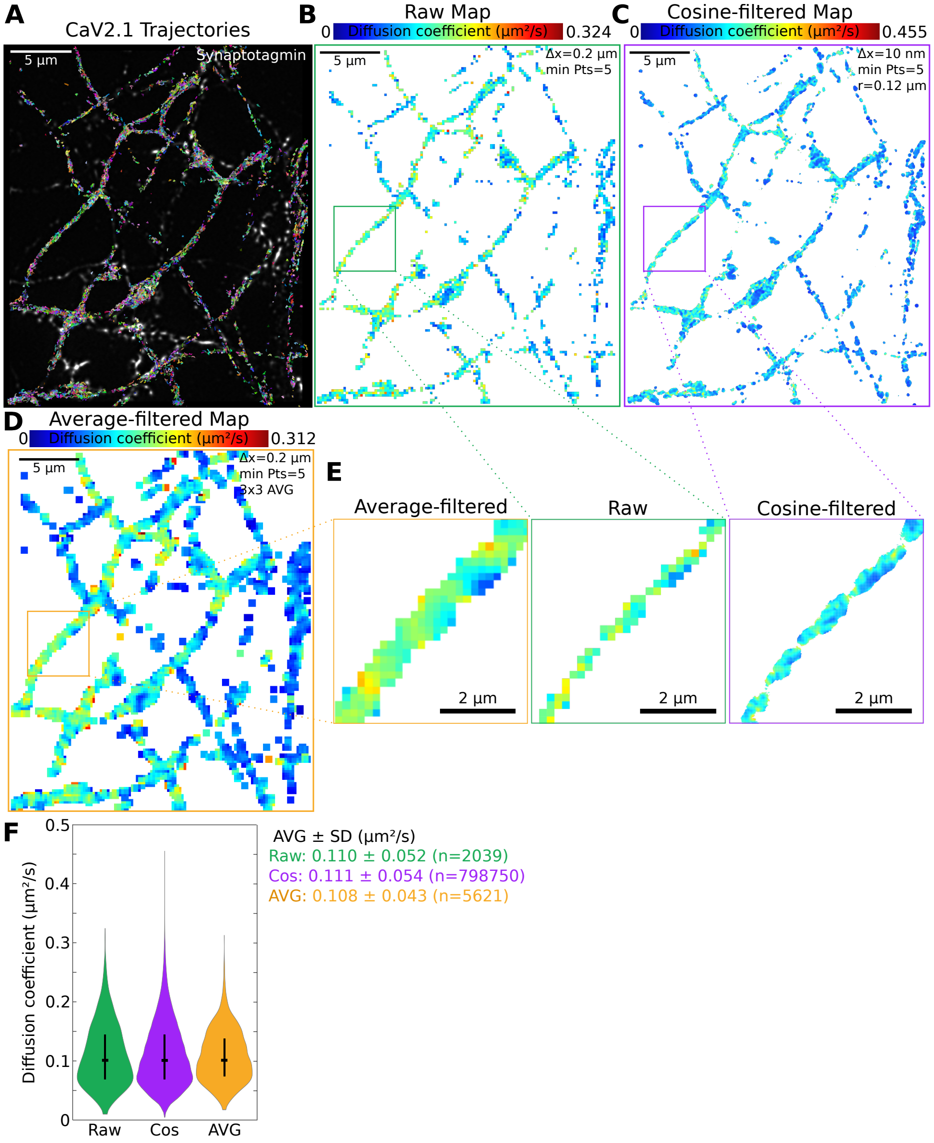

Figure S2: **Comparing the diffusion maps for CaV2.1 SPTs from three estimators**, related to STAR Methods. **A.** Individual CaV2.1+47 trajectories overlaid on top of the synaptotagmin pre-synaptic terminal marker's signal. **B.** Diffusion map computed from the classical estimator (Eq. 7 main text). **C.** Diffusion map computed with the cosine-estimator (Eq. 9 Main text). The cosine estimator is computed over a sliding window with a moving disk of radius $r_{filt} = 0.12$ μm and a grid size $\Delta x = 0.01$ μm. **D**. Map computed using a local average filter (using a 3×3 window around each bin). **E.** Comparison of the tree approaches from a representative regions. **F.** Violin plots of the diffusion coefficients computed in each bin for the three methods (n represents the number of bins).

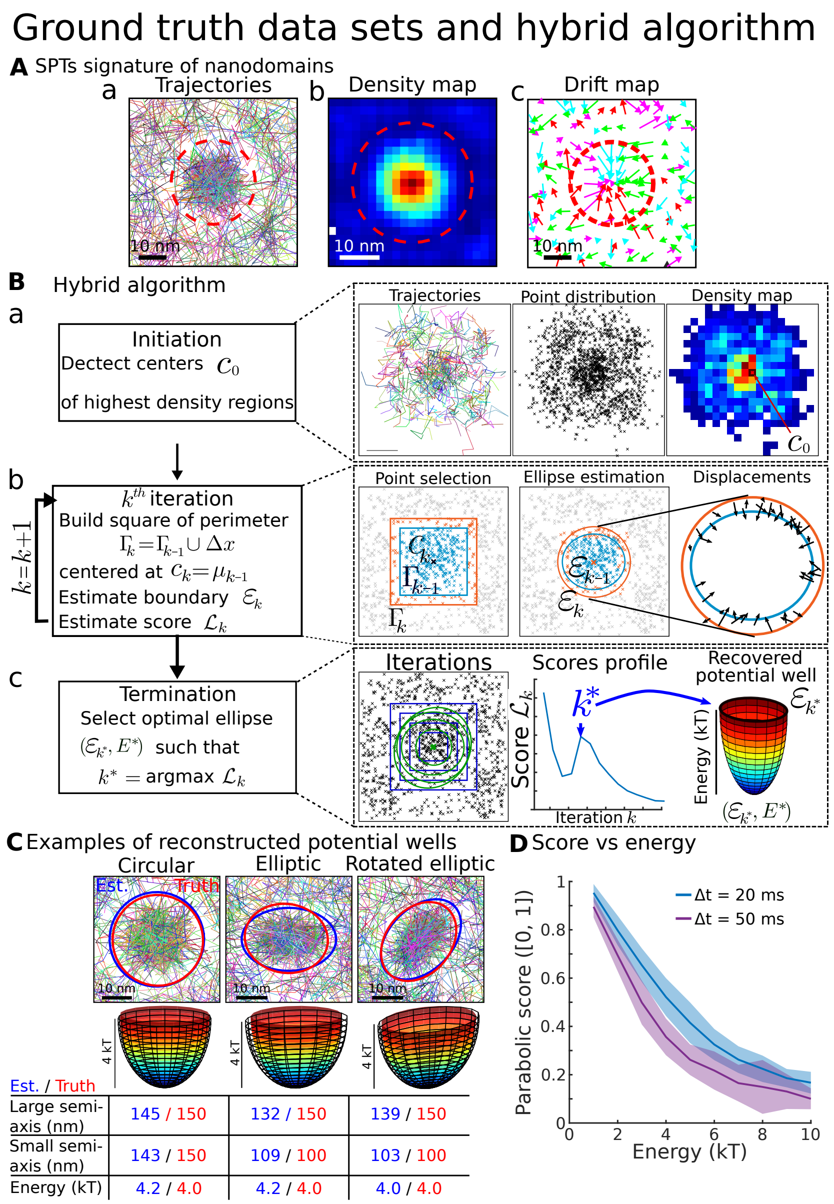

Figure S3: **Reconstruction algorithm of nanodomains from super-resolution SPTs**, related to Figures 2 and 3. **A.** a- Simulated trajectories attracted in a well of known boundary. b- density map c- drift field. **B.** Algorithm to reconstruct the center, boundary and energy of the nanodomain characterized as a potential well. Initiation of the center c_0 with an ellipse γ_0 and the center of mass μ_0. The error between the vector field and the reconstructed field is measured by the score S_0. The spatial resolution is fixed by Δx. k-iteration step: the domain is enlarged to Γ_k, the center is revaluated at point c_k and the error S_k is recomputed. In the termination step, the optimal value k^* is selected that minimizes the error S_{k^*}, leading to the optimal center and ellipse γ_{k^*}, for which the energy is computed as the ratio A/D of the field to the diffusion coefficient. **C**. Three examples of potential wells reconstructed from trajectories. **D** as a function of the energy of the well and depending on the acquisition time. The simulation, well detection algorithm and its parameters are the same as in Table S3.

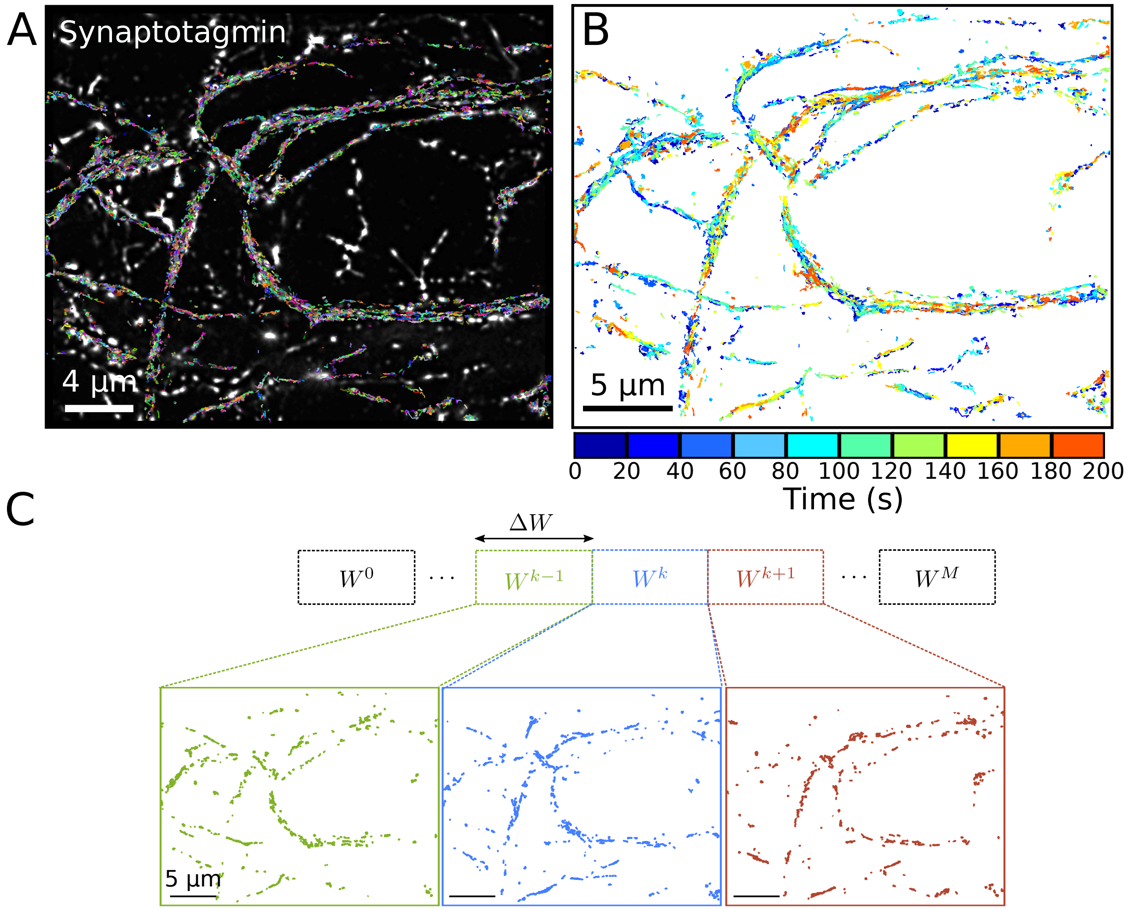

Figure S4: **Time Lapse analysis based on a sliding window**, related to Figures 2 and 3. **A.** Example of CaV2.1Δ47 SPTs plotted over the entire acquisition time and overlaid on top of the synaptotagmin pre-synaptic terminal marker's signal. **B.** Time-splitting of the dataset into 20 s time windows with no overlap. Trajectories are colored by the time window in they appear. **C.** Time-splitting principle: the entire duration spanned by the dataset is divided into windows of duration $\Delta W = 20$ s. In the case of overlapping windows, a trajectory belonging to two consecutive time windows will serve for the statistical estimations for each period.

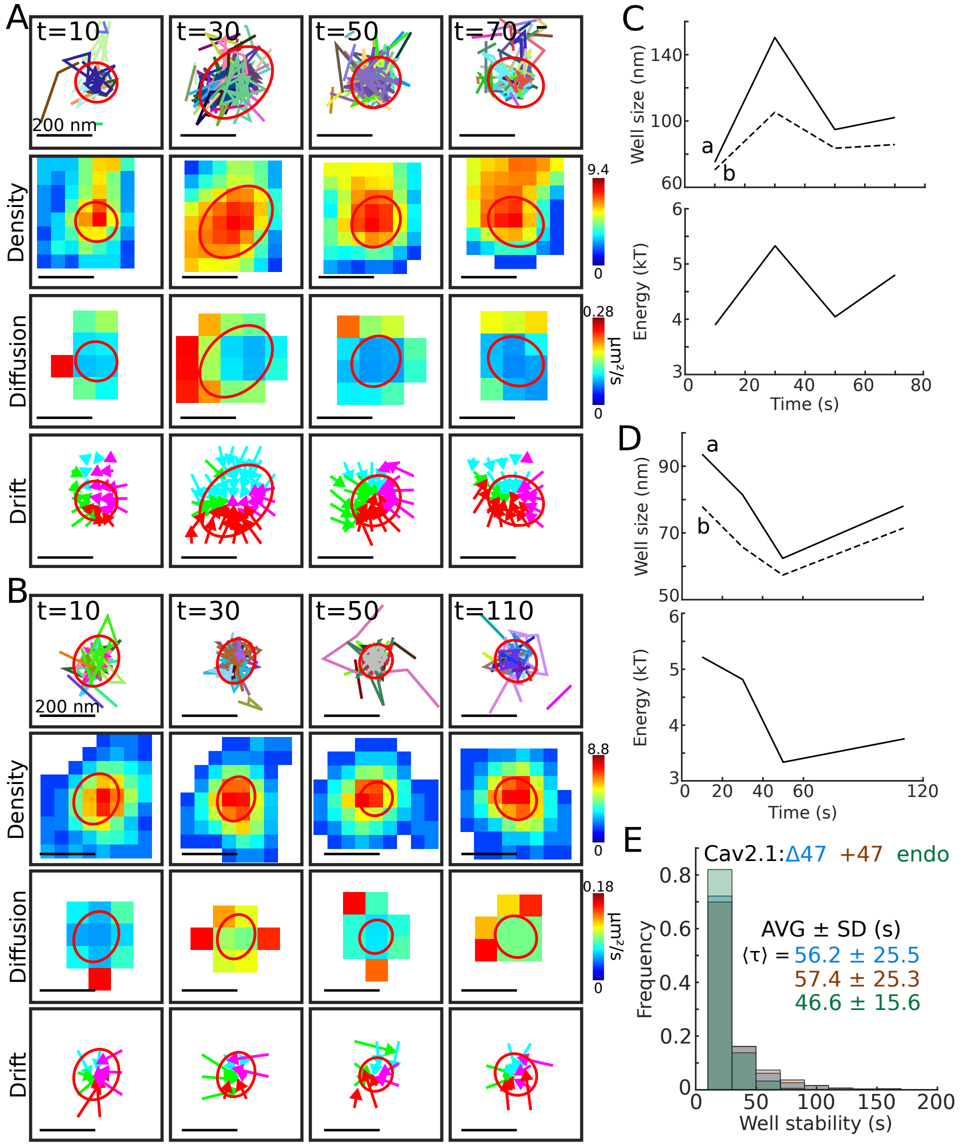

Figure S5: **Two examples of endogenous CaV2.1 nanodomains automatically identified during a time lapse analysis**, related to STAR Methods. **A.** A high density region identified as potential well in successive 20 s time windows. From top to bottom: individual trajectories, associated density, diffusion and drift maps. **B.** Another example of the temporal evolution of a potential well, similar to A. **C-D.** Temporal evolution of size and energy characteristics associated to the wells presented in A and B respectively. **E.** Population characteristics for three experimental conditions: CaV2.1Δ47, CaV2.1+47 or CaV2.1 showing the mean life time of potential wells extracted by an exponential fit (n values are given in Table S5).

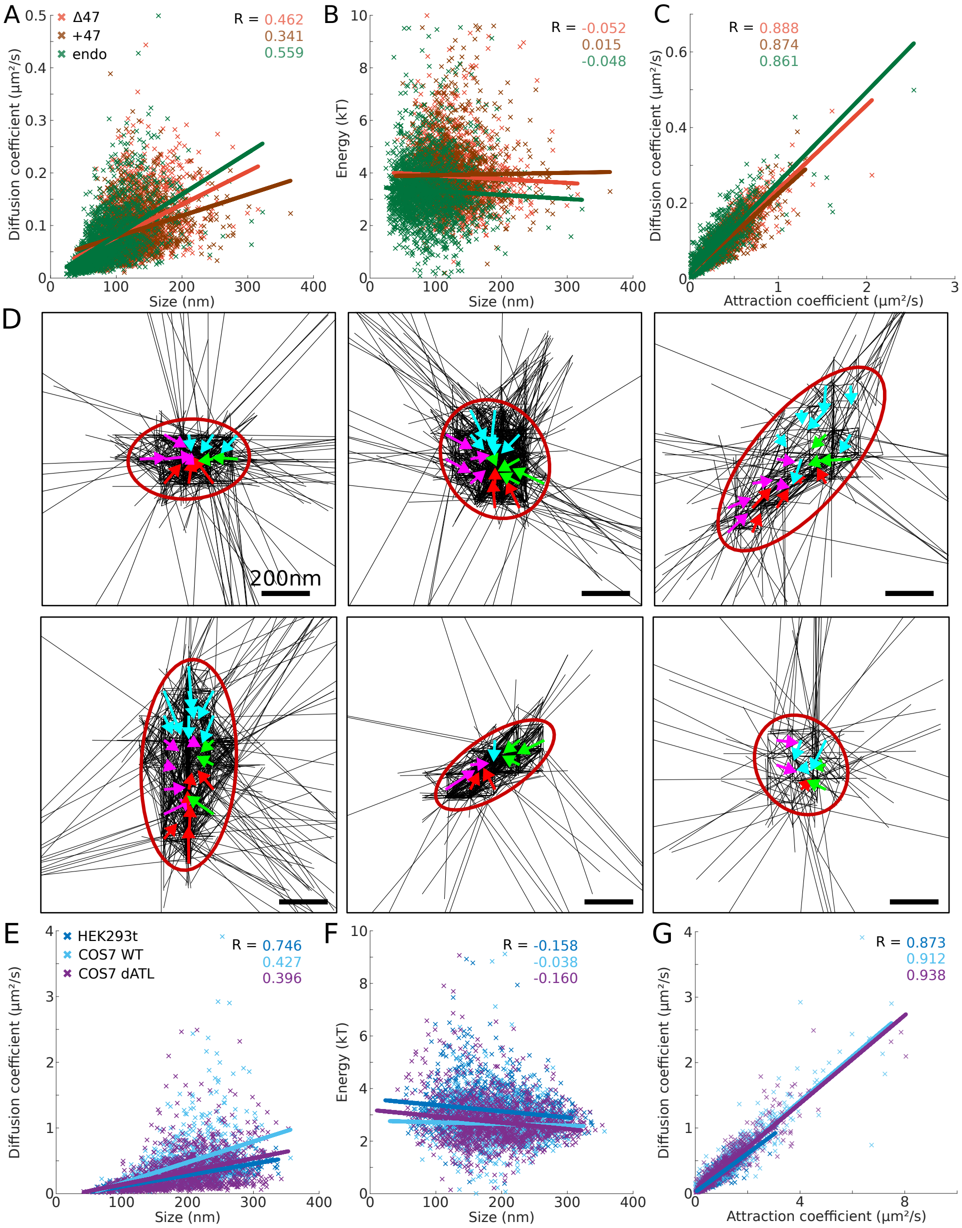

Figure S6: **Potential wells' characteristics**, related to Figures 2 and 3. **A.** Diffusion vs size (high correlation) **B.** energy vs size (no correlation). **C.** Diffusion vs attraction coefficient (high correlation). **D.** Six examples of converging arrows in potential wells associated to ER nodes. **E.** Distribution of the energy of the wells vs the size and the correlation coefficients. **F.** Energy vs size (no correlation). **G.** Diffusion coefficient vs attraction coefficient showing strong correlations between the two parameters. For A-C: $n = 1587, 1713, 1878$ for the $\Delta 47$, $+47$ and endogenous cases respectively; For E-G: $n = 1057, 859, 1250$ for the HEK293t, COS7WT and COS7dATL cases respectively.

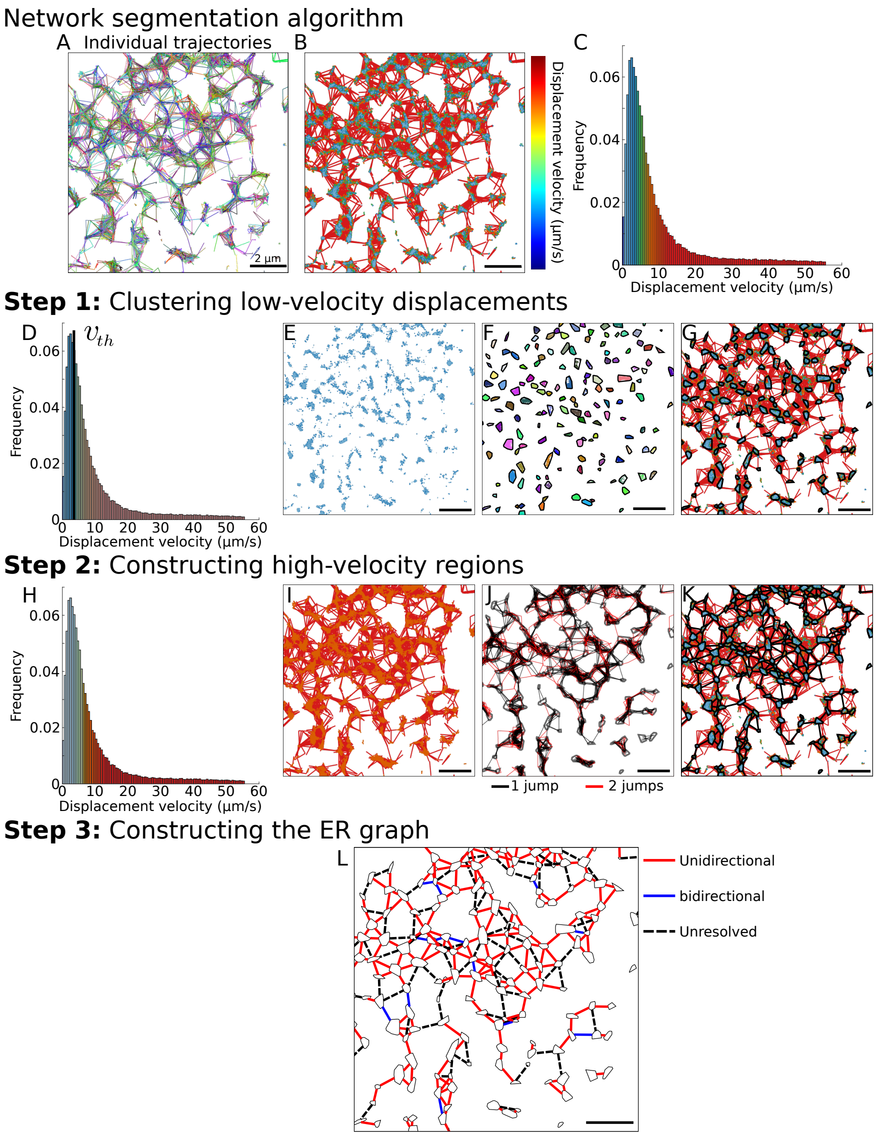

Figure S7: **Automatic network reconstruction from super-resolution SPTs**, related to Figures 4 and 7. **A.** Individually color-coded ER-luminal trajectories. **B.** the same trajectories as in A but where each displacement is color-coded by its instantaneous velocity. **C.** Distribution of instantaneous velocities color-coded according to their amplitude as in B. **D-G.** Extraction of the network nodes from low-velocity regions: a threshold v_{th} is selected on the distribution of instantaneous velocities (D) and the corresponding displacements are shown (E), these points are clustered using the dbscan algorithm (or a recursive version of it, see Method) and the convex hull of each cluster is computed to obtain its boundary (F). Finally, the corresponding boundaries are overlaid on top of the original trajectories (G). **H-K.** Extraction of the graph links from high-velocity jumps: we now select only the displacements with velocities above the threshold used in step 1 (H) shown in (I). We then filter them to keep only those that either: i. have their endpoints that fall into two separate ellipses (single-jump) extracted from step 1 or ii. successive displacements where the first point falls in an ellipse, the middle point is outside and the third point fall in a different ellipse (double-jump) (J) from which we obtained the reconstructed graph (K). **L.** A directionality analysis applied to the graph recovered from step 2 allowing to recover an oriented graph of the ER network.

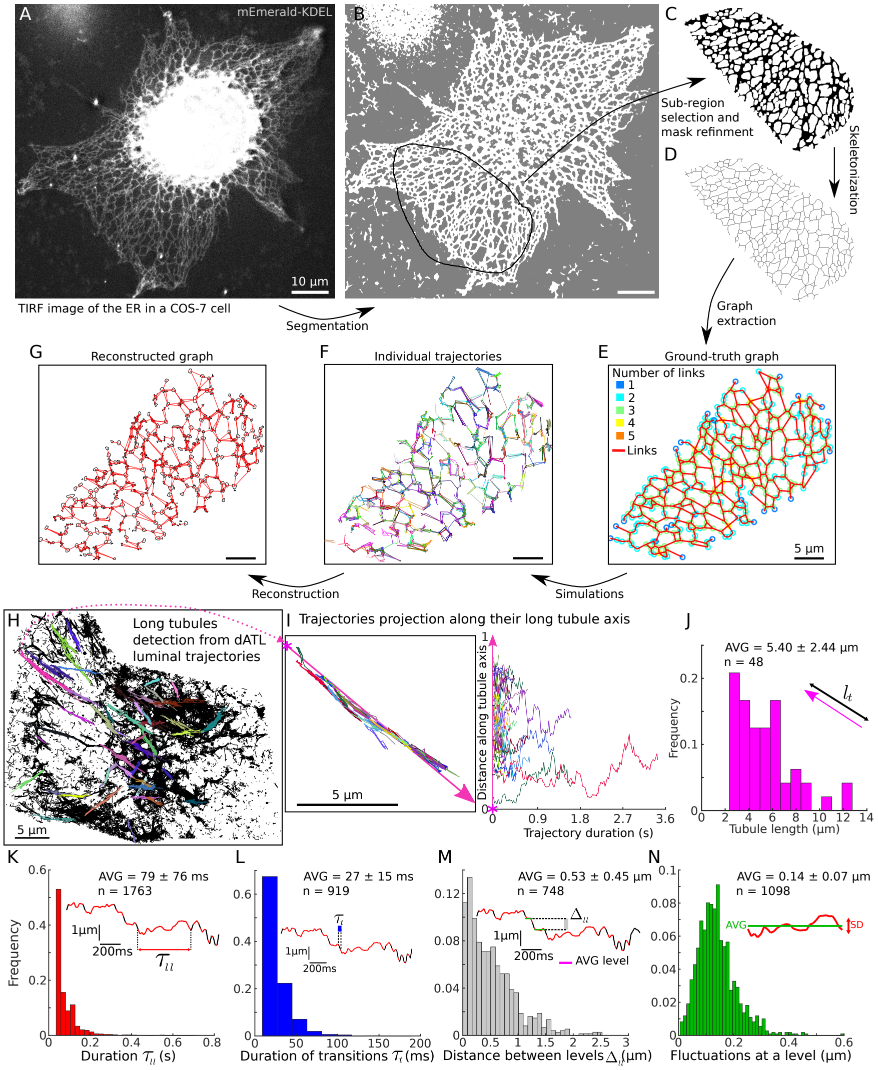

Figure S8: **Simulation of trajectories in an empirical ER network and dATL long tubule analysis**, related to STAR Methods. **A.** TIRF microscopy image of the ER network of a COS-7 cell transfected with an mEmerald-KDEL marker. **B.** Segmented image obtained with Ilastik where the ER network appears in white. **C.** Magnification on a well segmented sub-region of the ER after applying dilation filters. **D.** Skeletonization of the region from C. **E.** Graph extracted from the skeleton from D. where intersection are replaced by disks of radius $r = 400$ nm. The nodes are color-coded by the number of links they have. **F.** Individual trajectories generated from the geometry presented in E. **G.** Reconstructed graph obtain by applying the DBSCANRec algorithm to the trajectories presented in F. **H** SPTs (black) recorded in the ER of a COS-7 dATL cell with detected long-tubules (colored). **I** Magnification of a single detected long tubule (left) and projection of its trajectories along the tubule axis (right). **J** Length distribution for the long tubules detected in A (n: number of detected long tubules). **K-N** Decomposition of the projected motion of trajectories in long tubules showing stalling and transition between different levels. D: Duration distribution at a given level (n: number of detected levels), E: Distribution of transitions between two successive levels (n: number of transition events). F: Distribution of distances between two successive levels (n: number of distances between successive levels). G: Fluctuation amplitude of the positions inside a level (n: number of levels with enough data).

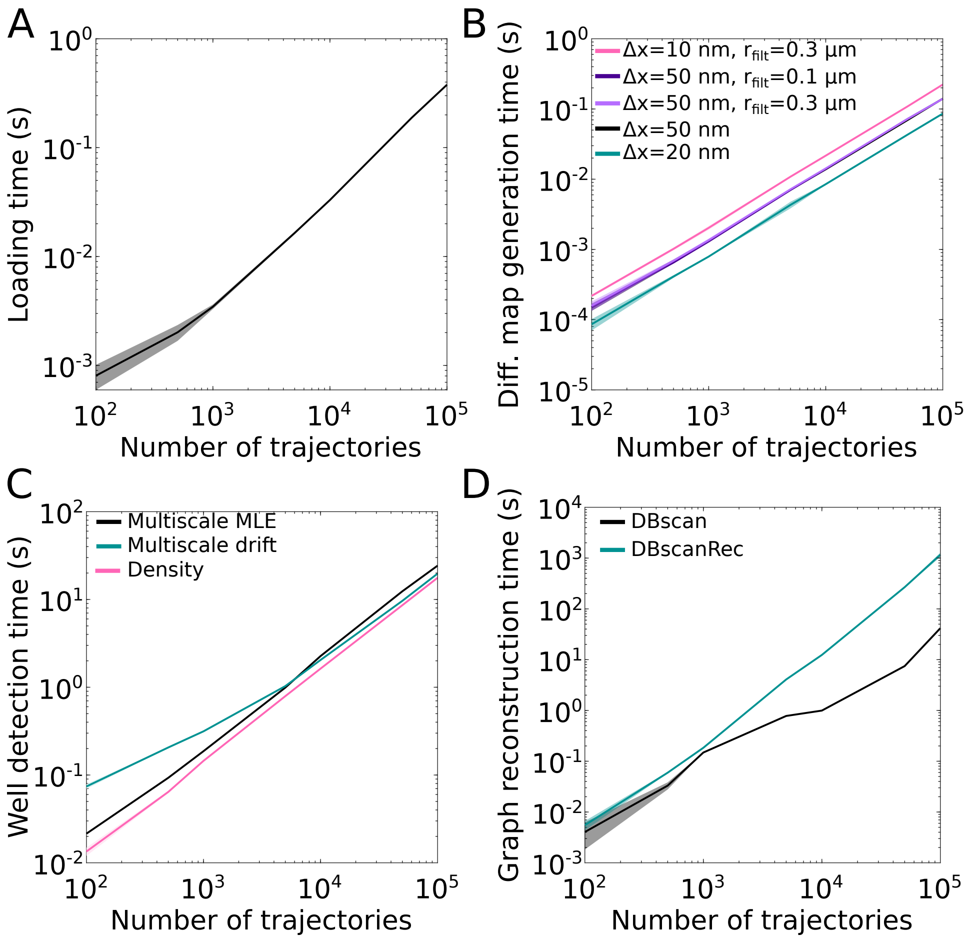

Figure S9: **Time benchmarks of the main plugin procedures**, related to STAR Methods. **A.** Loading time as a function of the number of trajectories. **B.** Diffusion maps reconstruction as a function of the number of trajectories and different set of parameters (with $minPts = 10$ in every case). **C.** Well detection time as a function of the number of trajectories for the three different methods implemented in the plugin. **D.** Graph reconstruction time as a function of the number of trajectories for the two different reconstruction algorithms implemented in the plugin. All values are given as average (solid line) ± std (shade) over 100 different sets of trajectories.

	Algo	Δt=20 ms		Δt=50 ms	
		E=4 kT	E=6 kT	E=4 kT	E=6 kT
$\|\|\mu - \mu_{true}\|\|$ (nm)	MLE	8 ± 6	5 ± 3	5 ± 4	4 ± 2
	Drift	26 ± 10	12 ± 5	19 ± 9	7 ± 3
	Dens	32 ± 5	24 ± 4	20 ± 4	14 ± 3
a (nm)	MLE	159 ± 15	146 ± 17	162 ± 17	140 ± 22
	Drift	136 ± 12	135 ± 10	142 ± 12	142 ± 11
	Dens	145 ± 24	145 ± 11	153 ± 14	148 ± 8
b (nm)	MLE	152 ± 14	140 ± 16	156 ± 17	134 ± 21
	Drift	126 ± 12	126 ± 10	132 ± 12	134 ± 11
	Dens	139 ± 23	139 ± 13	149 ± 14	144 ± 9
A (μm^2/s)	MLE	0.24 ± 0.03	0.29 ± 0.05	0.25 ± 0.04	0.27 ± 0.08
	Drift	0.12 ± 0.03	0.17 ± 0.03	0.09 ± 0.02	0.13 ± 0.02
	Dens	0.16 ± 0.02	0.22 ± 0.02	0.19 ± 0.02	0.26 ± 0.02
D (μm^2/s)	MLE	0.050 ± 0.002	0.051 ± 0.002	0.050 ± 0.003	0.050 ± 0.004
	Drift	0.043 ± 0.001	0.041 ± 0.001	0.034 ± 0.001	0.029 ± 0.001
	Dens	0.049 ± 0.003	0.051 ± 0.002	0.050 ± 0.003	0.051 ± 0.003
E (kT)	MLE	4.78 ± 0.52	5.72 ± 1.03	4.88 ± 0.67	5.28 ± 1.38
	Drift	2.71 ± 0.57	4.07 ± 0.63	2.75 ± 0.46	4.41 ± 0.62
	Dens	3.35 ± 0.27	4.40 ± 0.29	3.80 ± 0.23	5.04 ± 0.31
Error (%)	MLE	7.23 ± 3.89	6.43 ± 4.56	9.10 ± 4.59	10.05 ± 5.15
	Drift	13.82 ± 5.13	15.13 ± 3.98	17.45 ± 4.15	19.56 ± 3.61
	Dens	7.41 ± 6.42	7.32 ± 3.46	4.42 ± 3.84	4.79 ± 3.54

Table S1: **Estimation of the potential well characteristics from simulations**, related to STAR Methods. Comparing the hybrid MLE algorithm with the Density and the Drift algorithms for an circular well with $a = b = 150$ nm, $E = 4$ (resp. 6) kT corresponds to $A = 0.02$ (resp. $A = 0.03$) μm^2/s.

	Algo	Δt=20 ms		Δt=50 ms	
		E=4 kT	E=6 kT	E=4 kT	E=6 kT
$\|\|\mu - \mu_{true}\|\|$ (nm)	MLE	6 ± 3	3 ± 2	3 ± 2	3 ± 2
	Drift	19 ± 7	11 ± 5	15 ± 7	8 ± 4
	Dens	23 ± 5	16 ± 4	15 ± 3	10 ± 2
a (nm)	MLE	148 ± 6	139 ± 9	156 ± 7	145 ± 24
	Drift	124 ± 11	125 ± 11	129 ± 9	127 ± 11
	Dens	136 ± 20	128 ± 14	142 ± 22	129 ± 16
b (nm)	MLE	121 ± 6	113 ± 9	129 ± 8	124 ± 23
	Drift	104 ± 8	100 ± 8	108 ± 7	104 ± 10
	Dens	116 ± 17	111 ± 14	123 ± 20	112 ± 15
A ($\mu m^2/s$)	MLE	0.26 ± 0.02	0.34 ± 0.04	0.27 ± 0.02	0.34 ± 0.07
	Drift	0.12 ± 0.02	0.16 ± 0.03	0.08 ± 0.01	0.10 ± 0.02
	Dens	0.17 ± 0.02	0.23 ± 0.02	0.18 ± 0.02	0.25 ± 0.04
D ($\mu m^2/s$)	MLE	0.051 ± 0.002	0.052 ± 0.003	0.050 ± 0.003	0.049 ± 0.006
	Drift	0.040 ± 0.002	0.036 ± 0.001	0.030 ± 0.001	0.023 ± 0.001
	Dens	0.050 ± 0.002	0.051 ± 0.003	0.048 ± 0.004	0.051 ± 0.007
E (kT)	MLE	5.04 ± 0.30	6.48 ± 0.75	5.45 ± 0.33	6.92 ± 1.24
	Drift	3.00 ± 0.44	4.34 ± 0.64	2.83 ± 0.40	4.29 ± 0.76
	Dens	3.50 ± 0.22	4.54 ± 0.30	3.75 ± 0.23	4.82 ± 0.36
Error (%)	MLE	9.38 ± 3.48	7.14 ± 4.46	12.31 ± 4.53	11.38 ± 9.57
	Drift	14.21 ± 4.01	16.59 ± 3.73	20.12 ± 3.47	23.79 ± 3.87
	Dens	8.40 ± 5.45	9.62 ± 4.93	9.37 ± 6.79	9.89 ± 6.73

Table S2: **Estimation of the potential well characteristics from simulations**, related to STAR Methods. Comparing the hybrid MLE algorithm with the Density and the Drift algorithms for an elliptic well. The parameters are the same as for Table. S1 except that we use an elliptic boundary with $a = 150$ nm and $b = 100$ nm, $E = 4$ (resp. 6) kT corresponds to $A = 0.02$ (resp. $A = 0.03$) $\mu m^2/s$.

E (kT)	$\|\|\mu - \mu_{true}\|\|$ (nm)	a (nm)	b (nm)	A (μm^2/s)	D (μm^2/s)	E (kT)	Error (%)
1	21 ± 9	150 ± 23	145 ± 23	0.12 ± 0.01	0.047 ± 0.002	2.63 ± 0.11	29.49 ± 7.18
2	12 ± 7	144 ± 19	139 ± 19	0.15 ± 0.02	0.047 ± 0.003	3.06 ± 0.30	12.54 ± 6.88
3	7 ± 4	145 ± 24	140 ± 23	0.18 ± 0.03	0.049 ± 0.003	3.59 ± 0.60	9.27 ± 6.22
4	6 ± 4	148 ± 20	143 ± 19	0.22 ± 0.05	0.050 ± 0.003	4.31 ± 0.77	8.13 ± 5.15
5	4 ± 3	157 ± 17	151 ± 16	0.28 ± 0.05	0.050 ± 0.003	5.54 ± 0.90	7.01 ± 4.65
6	3 ± 2	163 ± 12	155 ± 11	0.34 ± 0.05	0.051 ± 0.003	6.69 ± 0.80	6.66 ± 4.15
7	3 ± 2	159 ± 14	152 ± 13	0.38 ± 0.06	0.051 ± 0.003	7.52 ± 1.11	6.48 ± 4.35
8	4 ± 14	157 ± 33	149 ± 24	0.41 ± 0.09	0.051 ± 0.004	8.00 ± 1.70	7.68 ± 8.65
9	3 ± 1	156 ± 18	149 ± 17	0.47 ± 0.10	0.051 ± 0.004	9.26 ± 1.80	7.32 ± 5.24
10	3 ± 1	155 ± 17	146 ± 17	0.51 ± 0.11	0.051 ± 0.004	9.97 ± 1.91	6.61 ± 5.86

Table S3: **Estimation of the potential well characteristics as a function of the energy**, related to STAR Methods. Based on a circular well ($a = b = 150$ nm) and using hybrid MLE algorithm and an acquisition time $\Delta t = 50$ ms.

Exp.	a (nm)	b (nm)	D (μm^2/s)	A (μm^2/s)	E (kT)	τ_e (ms)	n
Cav2.1 Δ47	143 ± 51	104 ± 33	0.091 ± 0.052	0.345 ± 0.209	3.9 ± 1.1	174 ± 134	1587
Cav2.1+47	145 ± 52	103 ± 33	0.087 ± 0.045	0.337 ± 0.189	3.9 ± 1.1	181 ± 141	1713
Cav2.1 endo	100 ± 47	73 ± 31	0.069 ± 0.051	0.224 ± 0.184	3.3 ± 1.2	94 ± 92	1878

Table S4: **Potential wells parameters extracted from CaV SPTs** related to Figure 2, n is the number of potential wells.

Exp.	Stability (s)	n
Cav2.1Δ47	56.2 ± 25.5	308
Cav2.1+47	57.4 ± 25.3	351
Cav2.1 endo	46.6 ± 15.6	284

Table S5: **CaV2.1 potential wells stability** related to Figures 2 and S5, n: number of families with as least two wells.

Exp.	a (nm)	b (nm)	D (μm^2/s)	A (μm^2/s)	E (kT)	τ_e (ms)	n
HEK293t	219 ± 71	155 ± 56	0.252 ± 0.136	0.787 ± 0.401	3.3 ± 0.9	101 ± 64	1057
COS-7 WT	230 ± 68	167 ± 45	0.461 ± 0.369	1.227 ± 0.984	2.7 ± 0.8	63 ± 46	859
COS-7 dATL	225 ± 70	163 ± 54	0.321 ± 0.292	0.868 ± 0.814	2.8 ± 0.9	106 ± 89	1250

Table S6: **Recovered potential wells parameters for ER datasets for HEK-293t, COS-7 WT and COS-7 dATL** related to Figure 3, n is the number of potential wells.

Algo.	# nodes	# links	# matched nodes	# matched links	Error (%)
DBSCAN	667 ± 22	687 ± 33	374 ± 4	336 ± 12	28.4 ± 2.2
DBSCANRec	638 ± 16	928 ± 38	391 ± 1	450 ± 8	8.1 ± 1.8

Table S7: **Estimation of the quality of the graph reconstruction algorithms**, related to Figures 5, 7, S7 and S8. Based on simulated trajectories and obtained from an optimised set of parameters (see Methods S1). The ground truth graph has 393 nodes and 550 links.
