# Supplementary material for: High-throughput super-resolution single-particle trajectory analysis reconstructs organelle dynamics and membrane reorganization
Source: Cell Rep Methods. 2022 Aug 22;2(8):100277. doi: 10.1016/j.crmeth.2022.100277 (PMC9421586; doi:10.1016/j.crmeth.2022.100277)
Supplement: Data S1. Methods S1 [file mmc2.pdf]

Data S1/Methods S1: Modeling, Ground Truth Simulations, Algorithms, Network Reconstruction from Trajectories

P. Parutto[1], J. Heck[2], M. Lu, C. Kaminski
E. Avezov, M. Heine[2] and D. Holcman[13] *

August 13, 2022

The Data S1/Methods S1 is organized as follows: first we present the evaluation of the new cosine-filtering method for reconstructing diffusion maps. Second, we present the numerical validations of the reconstruction algorithm using a ground truth data set generated by an Ornstein-Ulhenbeck process. Third, we present complementary procedures to extract potential wells from calcium voltage channel trajectories in neurons. Fourth, we present the validation procedure for the graph reconstruction method. Fifth, we discuss the application of the graph reconstruction and potential wells to the dATL-mutant of the endoplasmic reticulum. The figures are presented in the supplementary figure section (see also STAR Methods for technical details). Finally, we discuss the SPT acquisition procedures, performance evaluation of the plugin and give some guidelines on how to choose the algorithms parameters.

1 Diffusion map evaluation

1.1 Ground truth generation

To evaluate the capacity of our diffusion map algorithm to recover a diffusion field, we generated an artificial diffusion field based on a 2D Perlin Noise image (Fig. S1A). We then normalize the generated image by dividing it by

*[1] Group of Data Modeling and Computational Biology, IBENS, Ecole Normale Supérieure,75005 Paris, France. [2] Research Group Functional Neurobiology at the Institute of Developmental Biology and Neurobiology, Johannes Gutenberg University Mainz, Mainz, Germany and [3] DAMPT, University Of Cambridge, DAMPT and Churchill College CB30DS, United Kingdom.

its maximum value and rescale it in a selected range of diffusion coefficients $D_{min} = 0.0001\ \mu m^2/s$ and $D_{max} = 0.1\ \mu m^2/s$ to obtain the ground truth diffusion field D_G (Fig. S1B). We further constructed a set of 100 Brownian simulations (from eq. 5), composed of 1000 trajectories of 40 points each with a time step $\Delta t_{num} = 0.1$ ms and an acquisition time of $\Delta t = 20$ ms (Fig. S1C). We set the pixel size for the diffusion map at 20 nm leading to a field of view of $10.24 \times 10.24\ \mu m^2$. The first point of each trajectory is randomly (uniform distribution) generated inside the field of view and a trajectory is killed if it goes out of the field of view. The diffusion coefficient at each point of a trajectory is determined by the value of the pixel from the diffusion map in which the point falls. We also generated similar trajectories for a constant field with diffusion $D = 0.05\ \mu m^2/s$.

1.2 Evaluating the diffusion field

For each simulation, we generated diffusion maps with different parameters and compared the results with the ground truth field (Fig. S1D). We used two measures for comparison: the percentage of area recovered by the recovered map as well as the average error computed over each recovered map's bin. Given a ground truth field D_G and a recovered field D_R, the average error in $\mu m^2/s$ is given by:

$$err(D_G, D_R) = \frac{1}{N} \sum_i |D_G(\boldsymbol{X}_i) - D_R(\boldsymbol{X}_i)|, \tag{1}$$

where $|.|$ is the absolute value and i ranges over the N bins of center $\boldsymbol{X}_i$ contained in the recovered field D_R. When the bin centers in D_G and D_R do not align, we compute $D_G(\boldsymbol{X}_i)$ using a bilinear interpolation from its neighbor values. The results are presented in Fig.S1E for a constant diffusion field: the filtered and the non-filtered maps are producing similar error for a filter radius of 50 nm, but increasing the filtering radius to 100 or even 500 nm decreases the error, irrespective of the grid size Δx. Interestingly, increasing the filter radius leads to a reduced average error as well as an increase coverage while increasing the minimum number of points required in each bin has only a small effect on the error. The results are similar for the non-uniform field, as presented in Fig. S1F, although the best estimations (non-filtered with $\Delta x = 500$ nm and filtered with $r = 500$ nm) have errors higher than in the uniform case.

1.3 Increased accuracy of map reconstruction with cosine-filter estimators

We compare here the diffusion map computed with the classical super-resolution estimators (Eq. 7 Main text) against the estimators based on the cosine filter (Eq. 9 Main text) on acquired data of CaV2.1+47. The results are shown in Fig. S2, where the cosine estimators give a much higher spatial resolution, as it is based on two scales: a radius $r = 0.12\ \mu m$ for the moving disk, and a grid bin width $\Delta x = 0.01\ \mu m$. In this procedure, we keep the bins that contain at least 5 displacements.

2 Validation of the hybrid algorithm on synthetic trajectories

To validate the hybrid algorithm (STAR Method, Main text), we define a ground-truth data set of trajectories. This ensemble is generated by numerical simulations of a stochastic equation considering a single potential well.

2.1 Ground-truth data generated by a stochastic process and the associated numerical simulations

We define the ground-truth data as the ensemble of trajectories obtained by a truncated Ornstein-Uhlenbeck process, where the drift term is the gradient of a potential energy function given in eq. 12 (Main text). For a well of center $\boldsymbol{\mu}$ and boundary $\mathcal{B}$, the position $\boldsymbol{X}(t)$ of the process at time t satisfies the stochastic equation

$$\dot{\boldsymbol{X}} = \begin{cases} -\lambda(\boldsymbol{X}(t) - \boldsymbol{\mu}) + \sqrt{2D}\dot{W} & X \in \mathcal{B} \\ \sqrt{2D}\dot{W} & \text{otherwise.} \end{cases}, \tag{2}$$

where the diffusion coefficient is D (in $\mu m^2/s$) and the attraction coefficient is λ (s^{-1}). We use the Euler's scheme to discretize the equation:

$$\boldsymbol{X}(t + \Delta t_{num}) = \begin{cases} -\lambda(\boldsymbol{X}(t) - \boldsymbol{\mu})\Delta t_{num} + \sqrt{2D\Delta t_{num}}\boldsymbol{\eta} & X \in \mathcal{B} \\ \boldsymbol{X}(t) + \sqrt{2D\Delta t_{num}}\boldsymbol{\eta} & \text{otherwise.} \end{cases} \tag{3}$$

The random vector $\boldsymbol{\eta} = [\eta_1, \eta_2]$ is Gaussian with $\eta_1, \eta_2 \sim \mathcal{N}(0, 1)$ and Δt_{num} is the elementary time step. Because experimental trajectories are obtained

with a much coarser time step $\Delta t > 10$ ms, we first generated trajectories with a smaller time step $\Delta t_{num} = 1$ ms and then sub-sample the simulated trajectories, retaining one point every $\Delta t / \Delta t_{num}$.

2.2 Instantiation of the numerical simulations and estimation results

The procedure to generate ground truth trajectories for a well with boundary $\varepsilon = (\boldsymbol{\mu}, a, b, \varphi)$ is as follows:

1. We define the simulation region as a box B, centered at $\boldsymbol{\mu}$ and of size $3a \times 3b$.

2. Next, we simulate trajectories surrounding the well, following eq. 3, and choosing their initial points uniformly distributed in the $B - \varepsilon$ region (outside the well). Each trajectory is composed of 20 points and new trajectories are generated until reaching 2000 displacements in the $B - \varepsilon$ region.

3. Finally, we generate trajectories inside the well according to eq. 3 with their initial point uniformly distributed in ε. As for step. 2, each trajectory is 20 points long and new trajectories are generated until reaching 1000 displacements inside ε.

The result of such simulation is presented in Fig.S3A-a for individual trajectories. Step 2 of the simulation is important in order to provide a clear difference in dynamics and density between inside and outside the well, as shown in the corresponding density and drift maps in Fig.S3A-bc.

2.3 Parameters optimisation

In order to compare the different well detection algorithms over different datasets, we perform a grid search optimization to identify the set of parameters giving the most accurate reconstructed well for each simulation scenario. The quality of a reconstructed well $w_R = (\mu_{R,x}, \mu_{R,y}, a_R, b_R, A_R, D_R)$ against a ground truth well $w_G = (\mu_{G,x}, \mu_{G,y}, a_G, b_G, A_G, D_G)$ is given by the error function (expressed in percent):

$$err(w_R, w_G) = \frac{1}{6}\left(\frac{|\mu_{R,x} - \mu_{G,x}|}{\mu_{G,x}} + \frac{|\mu_{R,y} - \mu_{G,y}|}{\mu_{G,y}} + \frac{|a_R - a_G|}{a_G} + \frac{|b_R - b_G|}{b_G} + \frac{|A_R - A_G|}{A_G} + \frac{|D_R - D_G|}{D_G}\right) \times 100, \quad (4)$$

where $|.|$ is the absolute value. The smaller the error the better the reconstructed well. Based on this error, we run a grid search method on the

parameter space of each algorithm and for each simulation scenario and we keep the ensemble of parameters minimising eq. 4. We use the following ranges of parameters for the different detection algorithms:

MLE Parameters ranges:

> dx_min: $\{0.004, 0.006, 0.008, 0.01, 0.012, 0.014\}$,
> dx_max: $\{0.014, 0.016, 0.018, 0.02, 0.022, 0.024, 0.026\}$,
> dx_step: $\{0.001, 0.002\}$,
> ell_perc: $\{90, 95, 99\}$,
> ring_minPts: $\{3, 7, 11\}$.

Drift Parameters ranges:

> dx_min: $\{0.008, 0.012, 0.016, 0.02, 0.024, 0.028, 0.032\}$,
> dx_max: $\{0.028, 0.032, 0.036, 0.04, 0.044, 0.048, 0.042\}$,
> dx_step: $\{0.0025, 0.005\}$,
> ell_perc: $\{90, 95, 99\}$.

Dens Parameters ranges:

> loc_grid_dx: $\{0.025, 0.05, 0.1\}$,
> dr: $\{0.005, 0.01, 0.02\}$,
> r_min: $\{0.02, 0.04, 0.06\}$,
> r_max: $\{0.3, 0.4, 0.5\}$,
> rat_max_dist: $\{0.4, 0.5, 0.6\}$.

For a given ground truth well, each combination of parameters was assessed over 100 different simulations and we kept the combination with the smallest average error.

2.4 Comparison of different algorithms

We generated simulations for different sets of parameters: either circular (radius $r = 150$ nm) or elliptic boundaries (semi-axes lengths $a = 150$ nm, $b = 100$ nm); two energy levels $E = A/D = 4\ kT$ or $6\ kT$ by setting the attraction coefficient $A = 0.2\ \mu\text{m}^2/\text{s}$ or A $= 0.3\ \mu\text{m}^2/\text{s}$ and keeping the diffusion coefficient $D = 0.05\ \mu m^2/\text{s}$ constant; And two different acquisition times $\Delta t = 20$ ms and $\Delta t = 50$ ms. For each condition, we generated 100

repeats of the simulations presented in paragraph 2.2. We then used these datasets to test the capacity of our three algorithms (see Method in Main Text): hybrid algorithm (MLE), the algorithm based on the drift estimation (Drift) and the one based on the density of points (Dens) to estimate from the trajectories the geometry of the well (semi-axes), the diffusion coefficient, the associated energy and the attraction coefficient A.
The results are shown in Tables S1 and S2 and the corresponding parameters of the algorithms in the table "Optimised parameters for the well detection algorithms". First, we found that all estimation methods allowed to recover the parameters of the well within a 20% error range for the circular case and 25% for the elliptic case (as defined by eq. 4). In both cases, the MLE and Dens algorithm performed significantly better than the Drift (errors around 9% for MLE, 6% for Dens and 16% for Drift). The geometry of the well is overall well reconstructed with centers located around 30 nm of the true center. For the boundary, in the circular case the error was around 10 nm for the MLE and Dens algorithm whereas the Drift algorithm has a tendency to underestimate the well size.
In the elliptical case, the algorithms slightly underestimated the large semi-axis length and overestimated the small semi-axis length, the recovered ratio a/b being around 1.2 instead of 1.33 (ground truth). For the dynamical parameters A, D and E, the diffusion coefficient was well estimated (except for the Drift algorithm that lead to underestimations). The errors found in the energy originates from the fluctuation in the estimation of A which is harder to estimate as it relies on a precise estimation of the boundary. Note that D is constant inside the well.
Overall, although the Dens algorithm is the most precise, its performances are mostly driven by a good estimation of the boundary and it performs worse than the MLE algorithm to estimate the energy at $6kT$. Interestingly, the density algorithm even performed better in the circular case for $\Delta t = 50$ ms than when $\Delta t = 20$ ms, which can be explained as it does not rely on any drift estimation and so is almost independent on the choice of Δt. Finally, the present results show that elliptical wells are harder to reconstruct compared to circular ones.

2.5 Well detection for increasing energy levels

To test the ability of the multiscale MLE approach to detect wells with different energy level, we generated 100 sets of simulations following the procedure described in paragraph 2.2, for a circular well of size $r = 150$ nm, $D = 0.05$ $\mu\text{m}^2/s$, $\Delta t = 50$ ms and increasing values of $A = 0.05, 0.1, 0.15, ..., 0.5$ $\mu m^2/s$

b (nm)	Δt (ms)	E (kT)	Algo	Parameters
150	20	4	MLE	Δx_{min}:14, ringMinPts:3, Δx_{step}:2, ellPerc:99, Δx_{max}:20
			Drift	Δx_{min}:8, Δx_{step}:2.5, ellPerc:99, Δx_{max}:32
			Dens	ratMaxDist:500, r_{max}:500, Δr:10, locGridDx:10, r_{min}:60
		6	MLE	Δx_{min}:4, ringMinPts:11, Δx_{step}:2, ellPerc:99, Δx_{max}:26
			Drift	Δx_{min}:24, Δx_{step}:5, ellPerc:99, Δx_{max}:32
			Dens	ratMaxDist:400, r_{max}:500, Δr:10, locGridDx:10, r_{min}:40
	50	4	MLE	Δx_{min}:4, ringMinPts:3, Δx_{step}:1, ellPerc:99, Δx_{max}:14
			Drift	Δx_{min}:8, Δx_{step}:2.5, ellPerc:99, Δx_{max}:32
			Dens	ratMaxDist:400, r_{max}:300, Δr:10, locGridDx:10, r_{min}:60
		6	MLE	Δx_{min}:4, ringMinPts:11, Δx_{step}:1, ellPerc:99, Δx_{max}:26
			Drift	Δx_{min}:28, Δx_{step}:2.5, ellPerc:99, Δx_{max}:32
			Dens	ratMaxDist:400, r_{max}:500, Δr:10, locGridDx:10, r_{min}:20
100	20	4	MLE	Δx_{min}:14, ringMinPts:3, Δx_{step}:2, ellPerc:99, Δx_{max}:16
			Drift	Δx_{min}:16, Δx_{step}:2.5, ellPerc:99, Δx_{max}:28
			Dens	ratMaxDist:400, r_{max}:300, Δr:10, locGridDx:50, r_{min}:60
		6	MLE	Δx_{min}:14, ringMinPts:3, Δx_{step}:2 ,ellPerc:99, Δx_{max}:20
			Drift	Δx_{min}:16, Δx_{step}:2.5, ellPerc:99, Δx_{max}:32
			Dens	ratMaxDist:400, r_{max}:300, Δr:10, locGridDx:10, r_{min}:40
	50	4	MLE	Δx_{min}:14, ringMinPts:3, Δx_{step}:2, ellPerc:99, Δx_{max}:16
			Drift	Δx_{min}:8, Δx_{step}:2.5, ellPerc:99, Δx_{max}:28
			Dens	ratMaxDist:400, r_{max}:400, Δr:10, locGridDx:10, r_{min}:60
		6	MLE	Δx_{min}:4, ringMinPts:3, Δx_{step}:2, ellPerc:99, Δx_{max}:22
			Drift	Δx_{min}:20, Δx_{step}:2.5, ellPerc:99, Δx_{max}:48
			Dens	ratMaxDist:400, r_{max}:300, Δr:20, locGridDx:50, r_{min}:20

Table 1: **Optimised parameters for the well detection algorithms** associated to Tables S1 and S2. The parameters $\Delta x_{min}, \Delta x_{max}, \Delta x_{step}, \Delta r, r_{min}, r_{max}$, ratMaxDist and locGridDx are given in nm.

leading to energies $E = 1, 2, 3, ...10$ kT.
For each energy level, we ran the parameter optimisation procedure to identify the best parameters for the algorithm. The results are summarized in Table S3: in all cases, the relative error with the ground truth is < 30 % and < 10 % for $E > 2$ kT (last column). For all energy levels, the centers, radii and diffusion coefficients are correctly estimated but the attraction coefficients are systematically overestimated. For $E > 2$ kT the algorithm performs better. The optimized parameters found for each energy levels are shown in the table "Optimised parameters for different energy levels for the hybrid MLE algorithm" with the associated parabolic scores in Fig.S3D, allowing a quantification of the well detection for different energy levels. Note that increasing the acquisition time Δt tends to improve the parabolic score of the detected wells for all energy levels.

E (kT)	Δx_{min} (nm)	Δx_{max} (nm)	Δx_{step} (nm)	ringMinPts	ellPerc
1	4	14	1	3	90
2	4	14	1	11	95
3	6	16	1	3	95
4	4	14	1	3	99
5	6	22	2	11	99
6	4	26	1	11	99
7	6	26	1	7	99
8	10	26	1	7	99
9	6	26	1	3	99
10	6	26	1	3	99

Table 2: **Optimised parameters for different energy levels for the hybrid MLE algorithm** associated to Table S3.

2.6 Well detection for Brownian motion

To test the false positive rates of our algorithm, we applied the optimisation procedure described in sub-section 2.3 for simulation scenarii when there is no potential well, thus the attraction field constant is $A = 0$, with energy $E = 0kT$. For each well detection algorithm, we report in the table "Estimation of the potential well characteristics for $E = 0$ kT wells", the detection percentage (number of times the algorithm returned a potential well out of 100 simulations) as well as the average well parabolic score. Even though there is no well, the algorithms report energies ~ 2 kT for the MLE and

Density and $\sim 0.5\ kT$ for the drift: indeed the MLE and Density algorithms do not directly use the drift field to compute the attraction coefficient A whereas the Drift algorithm does. Although the MLE and Dens algorithms return a result in most cases (Detected wells line), the parabolic score associated to the results are in all cases > 0.9 which clearly indicate a lack of attraction field and can be used as a criteria to filter out those wells. The parameters used for the different detection algorithms in these cases are reported in the table "Optimised parameters for the well detection for $E = 0$ kT".

		Δt=20 ms		Δt=50 ms	
	Algo	$b = 150$ nm	$b = 100$ nm	$b = 150$ nm	$b = 100$ nm
$\|\|\mu - \mu_{true}\|\|$ (nm)	MLE	151 ± 43	108 ± 45	100 ± 39	56 ± 28
	Drift	162 ± 85	70 ± 0	72 ± 2	69 ± 0
	Dens	24 ± 9	13 ± 7	11 ± 6	10 ± 5
a (nm)	MLE	158 ± 37	198 ± 49	162 ± 32	226 ± 45
	Drift	115 ± 1	110 ± 0	92 ± 9	93 ± 0
	Dens	117 ± 29	116 ± 29	128 ± 46	79 ± 9
b (nm)	MLE	150 ± 36	191 ± 48	155 ± 30	216 ± 40
	Drift	106 ± 3	108 ± 0	87 ± 7	92 ± 0
	Dens	114 ± 28	114 ± 29	124 ± 44	78 ± 8
A ($\mu m^2/s$)	MLE	0.10 ± 0.01	0.10 ± 0.01	0.10 ± 0.01	0.10 ± 0.01
	Drift	0.02 ± 0.00	0.00 ± 0.00	0.01 ± 0.00	0.01 ± 0.00
	Dens	0.09 ± 0.01	0.09 ± 0.01	0.09 ± 0.01	0.07 ± 0.01
D ($\mu m^2/s$)	MLE	0.047 ± 0.002	0.046 ± 0.002	0.046 ± 0.001	0.046 ± 0.001
	Drift	0.049 ± 0.000	0.048 ± 0.000	0.048 ± 0.002	0.049 ± 0.000
	Dens	0.046 ± 0.003	0.043 ± 0.005	0.046 ± 0.002	0.037 ± 0.005
E (kT)	MLE	2.21 ± 0.14	2.20 ± 0.12	2.14 ± 0.16	2.07 ± 0.15
	Drift	0.44 ± 0.04	0.08 ± 0.00	0.24 ± 0.05	0.16 ± 0.00
	Dens	2.05 ± 0.06	2.03 ± 0.05	2.06 ± 0.08	2.02 ± 0.04
Parabolic score	MLE	0.99 ± 0.01	1.00 ± 0.00	1.00 ± 0.00	1.00 ± 0.00
	Drift	0.97 ± 0.03	1.00 ± 0.00	0.98 ± 0.01	0.93 ± 0.00
	Dens	0.99 ± 0.01	0.99 ± 0.01	0.99 ± 0.01	0.99 ± 0.01
Detected wells (%)	MLE	86	100	100	100
	Drift	3	1	5	1
	Dens	100	100	100	100

Table 3: **Estimation of the potential well characteristics for $E = 0$ kT wells** for a well with $a = 150$

b (nm)	Δt (ms)	Algo	Parameters
150	20	MLE	Δx_{min}:4, ringMinPts:11, Δx_{step}:2, ellPerc:90, Δx_{max}:14
		Drift	Δx_{min}:8, Δx_{step}:5, ellPerc:99, Δx_{max}:44
		Dens	ratMaxDist:400, r_{max}:500, Δr:10, locGridDx:50, r_{min}:60
	50	MLE	Δx_{min}:12, ringMinPts:3, Δx_{step}:1, ellPerc:90, Δx_{max}:14
		Drift	Δx_{min}:8, Δx_{step}:2.5, ellPerc:99, Δx_{max}:44
		Dens	ratMaxDist:400, r_{max}:300, Δr:10, locGridDx:100, r_{min}:60
100	20	MLE	Δx_{min}:12, ringMinPts:3, Δx_{step}:1, ellPerc:90, Δx_{max}:14
		Drift	Δx_{min}:28, Δx_{step}:5, ellPerc:95, Δx_{max}:48
		Dens	ratMaxDist:400, r_{max}:300, Δr:10, locGridDx:100, r_{min}:60
	50	MLE	Δx_{min}:12, ringMinPts:3, Δx_{step}:1, ellPerc:90, Δx_{max}:14
		Drift	Δx_{min}:28, Δx_{step}:2.5, ellPerc:99, Δx_{max}:36
		Dens	ratMaxDist:400, r_{max}:300, Δr:5, locGridDx:100, r_{min}:60

Table 4: **Optimised parameters for the well detection for $E = 0$ kT** associated to the previous table "Estimation of the potential well characteristics". The parameters $\Delta x_{min}, \Delta x_{max}, \Delta x_{step}, \Delta r, r_{min}, r_{max}$, ratMaxDist and locGridDx are given in nm.

2.7 Confined Brownian motion

Here, we tested the ability of our algorithm to differentiate between confined Brownian motion versus potential well. Indeed, stochastic discretization can induce an artificial drift field for trajectories bouncing on the boundary wall. To that end, we assessed the parabolic score of the drift field generated by confined Brownian motion for different confinement radii. We simulated trajectories of a confined Brownian motion in a circle of radius r as follows:

1. We generate each displacement of a trajectory using Euler's scheme

$$\boldsymbol{X}(t + \Delta t_{num}) = \boldsymbol{X}(t) + \sqrt{2D\Delta t_{num}}\boldsymbol{\eta}, \quad (5)$$

   where similarly to eq. (3), Δt_{num} is the simulation timestep (usually 1 ms), D is the diffusion coefficient and $\boldsymbol{\eta}$ is a vector of i.i.d Gaussian variables.

2. We then tested whether the generated point is located inside the disk : $||\boldsymbol{X}(t + \Delta t_{num})|| < r$, where $||.||$ is the Euclidean norm. If it was

inside we retained the point, otherwise we used a reflection procedure, as described below:

3. When $X(t + \Delta t_{num})$ is outside the boundary, then the segment s : $[\boldsymbol{X}(t), \boldsymbol{X}(t + \Delta t_{num})]$ intersects the circle C_r of radius r. The point resulting from the collision $\boldsymbol{P}_c$ corresponds to the reflection of $\boldsymbol{X}(t + \Delta t_{num})$ with respect to the tangent line T_i at the intersection point $\boldsymbol{P}_i$ between s and C_r. The intersection point $\boldsymbol{P}_i$ lies along the segment s and thus $\boldsymbol{P}_i = \boldsymbol{X}(t) + t^*(\boldsymbol{X}(t + \Delta t_{num}) - \boldsymbol{X}(t))$ with $t^* \in [0, 1]$ is solution to

$$||\boldsymbol{X}(t) + t^*(\boldsymbol{X}(t + \Delta t_{num}) - \boldsymbol{X}(t))|| = r. \tag{6}$$

We solve the previous equation to find $\boldsymbol{P}_i$ and then compute the outward pointing normal $\boldsymbol{n}_i$ to C_r at point $\boldsymbol{P}_i$,

$$\boldsymbol{n}_i = \frac{\boldsymbol{P}_i}{||\boldsymbol{P}_i||}, \tag{7}$$

from which we derive the minimal distance d between $\boldsymbol{X}(t + \Delta t_{num})$ and T_i as

$$d = \boldsymbol{n}_i \cdot (\boldsymbol{X}(t + \Delta t_{num}) - \boldsymbol{P}_i), \tag{8}$$

with $\cdot$ the dot product. Finally, the reflected point is obtained by the classical Descartes-Snell reflection:

$$\boldsymbol{P}_c = \boldsymbol{X}(t + \Delta t_{num}) - 2d\boldsymbol{n}_i. \tag{9}$$

Finally, we set $\boldsymbol{X}(t + \Delta t_{num}) = \boldsymbol{P}_c$ and proceed to the next timestep by going back to step 1.

Following this procedure, we generated simulations for different confinement sizes $r = 50, 100, 150, 200, 250, 300$ nm. For each confinement value, we generated 100 independent repeats, with the following parameters: $D = 0.05$ $\mu m^2/s$, $\Delta t = 20$ ms, $\Delta t_{num} = 0.1$ ms, $N_{pts} = 50$ points per trajectory, points density $dens = 50000$ μm^{-2}, $N_{trajs} = \lceil \frac{\pi r^2 \times dens}{N_{pts}} \rceil$. For each simulation, we computed the drift map centered at the center of the confinement region for different bin sizes $\Delta x = 20, 50, 70$ nm and the parabolic score associated to each map (eq. 38 Main text). The results are presented in the table "Parabolic score generated by drift maps from confined Brownian motion" where $\emptyset$ values mean that there were not enough bins falling inside the well

to compute the parabolic score (it happens when the bin size is to large compared to the confinement size). To conclude, the bin and confinement sizes influence the score of the drift field. To prevent bouncing trajectories to influence the parabolic field, the grid bin needs to be much smaller than the confinement radius. Note that the parabolic score can be greatly refined by using the cosine-filtering approach (presented for Diffusion maps in section 2.1 Main text) to generate the drift field as presented in the table "Parabolic score generated by cosine-filetered drift maps from confined Brownian motion". This method allows to use much smaller bins (1, 5 or 10 nm) and produces a smoother field, allowing to distinguish confinement and potential wells for regions as small as 100 nm radius.

r (nm)	$\Delta x = 20$ (nm)	$\Delta x = 50$ (nm)	$\Delta x = 70$ (nm)
50	0.113 ± 0.031	$\emptyset$	$\emptyset$
100	0.275 ± 0.027	0.076 ± 0.021	$\emptyset$
150	0.500 ± 0.030	0.174 ± 0.024	0.105 ± 0.024
200	0.618 ± 0.020	0.322 ± 0.027	0.289 ± 0.036
250	0.731 ± 0.019	0.430 ± 0.027	0.321 ± 0.025
300	0.768 ± 0.014	0.543 ± 0.029	0.529 ± 0.046

Table 5: **Parabolic score generated by drift maps from confined Brownian motion** for different confinement radii and grid sizes. $\emptyset$ indicates cells were not enough data could be obtained. Red entries have values < 0.5 and are considered as potential wells while blue entries have values > 0.5.

r (nm)	$\Delta x = 1$ (nm)	$\Delta x = 5$ (nm)	$\Delta x = 10$ (nm)
50	0.160 ± 0.072	0.053 ± 0.012	0.053 ± 0.012
100	0.892 ± 0.116	0.162 ± 0.020	0.163 ± 0.020
150	0.930 ± 0.092	0.659 ± 0.058	0.328 ± 0.027
200	0.917 ± 0.103	0.989 ± 0.014	0.476 ± 0.022
250	0.944 ± 0.073	0.991 ± 0.011	0.756 ± 0.027
300	0.912 ± 0.118	0.992 ± 0.011	0.977 ± 0.012

Table 6: **Parabolic score generated by cosine-filetered drift maps from confined Brownian motion** (with disk radius $r_{filt} = 20$ nm) for different confinement radii and grid sizes. Only drift bins with more than 5 values were considered and the parabolic score was computed only for ellipses with at least 5 drift values. Red entries have values < 0.5 and are considered as potential wells while blue entries have values > 0.5.

3 SPTs analysis

3.1 Time lapse analysis of SPT trajectories

We define the time lapse analysis as a sequence of possibly overlapping time windows $W_1, \dots, W_N$ of duration ΔW. We then split the ensemble of trajectories into these time windows and independently analyze them. A schematic example is presented Fig. S4, where we show trajectories slitted in 20s time windows with no overlap. We applied this procedure when mentioned in the text.

3.2 CaV2.1 potential wells characteristics

Table S4 summarizes the geometrical properties of the wells found in CaV variants while the table "Ratio of trajectories appearing inside potential wells" shows the proportion of trajectories appearing in wells.

Exp.	Ratio of trajs. in wells	n
Cav2.1Δ47	0.402 ± 0.100	19
Cav2.1+47	0.396 ± 0.129	19
Cav2.1 endo	0.316 ± 0.066	11

Table 7: **Ratio of trajectories appearing inside potential wells.** Results are given as AVG ± STD, n is the number of datasets.

The results for mean residence time τ_e of channels inside a well (see Main Text) is presented in Table S4, showing the long-time stability of the wells.

CaV nanodomain are automatically identified during a time lapse analysis, as shown in Fig. S5 and the correlations between different well parameters are presented for CaV2.1 datasets in Fig.S6.

4 Graph Reconstruction Algorithm

The GRA consists of three steps: 1-identifying the HDRs formed by low-velocity (blue regions) trajectory displacements forming the nodes of the graph (Fig. S7D-G), 2- determining the high-velocity trajectory displacements connecting the previously detected nodes (Fig. S7H-K) and 3- constructing the associated graph (Fig. S7L), as described in Method. The first step of the method relies on the density-based clustering algorithm (dbscan), which requires that the distribution of trajectories be quite heterogeneous

so that small regions of high density are well-separated. In an additional step, a direction can be attributed to the links (i.e. tubules) based on the percentage of trajectories going in the same direction between two nodes: when the percentage is around 50%, the node is bidirectional, however if the percentage is much lower, the node is defined as unidirectional.

5 Validation of the graph reconstruction algorithms on synthetic trajectories

5.1 Network extraction

To evaluate the graph reconstruction algorithms, we used a procedure to simulate jump-diffusion motion from a graph extracted from a ER network. Starting from an image of a fluorescently marked ER in a COS-7 cell (Fig. S8A), we segmented the image using the ilastik program. This procedure segments the ER network (white regions in Fig. S8B) over the background. Then, we binarized the resulting image and applied twice a 3×3 binary dilation filter (Fig. S8C) from which we computed the skeleton image S using the ImageJ skeletonize plugin (Fig. S8D). Next, we used a custom Python code to transform the skeleton into a graph data structure implementing the following procedure:

1. We created an image C where for each white pixel of the skeleton image S, we computed the number of pixels in a 3x3 neighborhood around it

$$C = (S * 1_{3,3}) \times S, \tag{10}$$

   where $*$ is the convolution, $1_{3,3}$ is the 3×3 unity kernel and $\times$ is the elementwise matrix multiplication.

2. To find the nodes of the network, we binarized C by thresholding $C > 2$ (all the pixels with more than two neighbors) and extracted the resulting connected components, the center of each component form the nodes of the network;

3. To find the tubules, we applied a similar procedure but thresholding with $C \leq 2$ (all the pixels with at most 2 neighbors), this time the different connected components correspond to the tubules of the network.

4. Finally, we formed the reconstructed graph by attaching each tubule (from step 3) to its connecting nodes (from step 2). To this end, we determined the endpoints of each tubule connected component extracted in step 3 and associated each endpoint to a node. To this end, we find the label (there must be at most one) appearing in a 3×3 neighborhood of each endpoint in the labelled $C > 2$ image from step 2. We discarded tubules possessing less than two connecting nodes.

The result of such procedure is presented in Fig. S8E, where we defined nodes as circles of radius $r = 400$ nm. As can be seen, the extracted network recapitulates the empirical ER structure from Fig.S8D.

5.2 Trajectories simulations

To generate trajectories inside the reconstructed network, we used a custom Matlab code. The parameters for these simulations are: r (in nm) the radius of the circular nodes and $p_{jump} \in [0, 1]$ the probability of a trajectory to jump from a node to one of its neighbor. The simulation proceeds as follows:

1. For each trajectory, we randomly selected a starting node N and assigned the first point of the trajectory as the center of the node;

2. Then, at every time step, we generated a random number $p \sim U([0, 1])$:

   (a) if $p < p_{jump}$: we performed a jump by randomly selecting a neighbor of N as the destination. Then we draw a random integer $k \sim U(\{1, 2\})$, when $k = 1$ the jump is made in one step and the destination is a random point inside the destination node's circle, if $k = 2$ the jump is made in two steps in which case we split the jump by adding a randomly selected point along the [start, destination] segment.

   (b) Otherwise, the trajectory undergoes a confined Brownian motion inside the node circular domain (see paragraph 2.7 for the details of the simulation of confined Brownian motion).

The generated trajectories recapitulated the diffusion model and followed the network structure as shown in Fig. S8 for 50000 trajectories each possessing 10 points, $\Delta t = 20$ ms and $p_{jump} = 0.33$. An example of reconstructed graph obtained from these trajectories with our recursive DBScan algorithm is presented in Fig. S8F.

5.3 Graph reconstruction evaluation

In order to evaluate the quality of a reconstructed graph with respect to a ground truth network (Fig.S8F versus Fig.S8D), we followed a procedure similar to the evaluation of potential wells, described in section 2.3). To compare the ground truth G_G and the recovered G_R graphs, we first define a mapping $G_R \to G_G$ of the nodes and links. The node mapping is obtained by computing for each node n of G_R, the set s_n of all nodes from G_G with an intersection:

$$s_n(G_G) = \{n' \in G_G | n \cap n' \neq \emptyset\} \tag{11}$$

Once s_n is evaluated, we derive the number of matched nodes $\#_n$ by counting the number of nodes from G_R associated with exactly one node from G_G:

$$\#_n(G_R \to G_G) = \sum_{n \in G_R} 1_{|s_n(G_G)|=1}, \tag{12}$$

where $1_{a=b}$ equals 1 when $a = b$ and 0 otherwise and $|s|$ is the size of the set s. The link mapping m is obtained by considering for each pair of connected nodes n, n' from G_R, whether any pair of their matched nodes are connected in G_G:

$$m_{n,n'}(G_G) = \begin{cases} 1 \text{ if } \exists(\eta \in s_n(G_G), \eta' \in s_{n'}(G_G) | C_{G_G}(\eta, \eta') > 0) \\ 0 \text{ otherwise} \end{cases}, \tag{13}$$

where C_G is the connectivity matrix of the graph G with $C_G(n, n') > 0$ if nodes n and n' are connected in G. Using this measure, we derive the number of matched links $\#_l$ from G_R to G_G as

$$\#_l(G_R \to G_G) = \sum_{n,n' \in G_R | C_{G_R}(n,n')>0} m_{n,n'}(G_G). \tag{14}$$

Finally, we define the error associated to the reconstruction between the reconstructed graph G_R and the ground truth graph G_G (in percent) as:

$$err(G_R, G_G) = \frac{1}{2}\left(\frac{|\#_n(G_R \to G_G) - \#_n(G_G)|}{\#_n(G_G)} + \frac{|\#_l(G_R \to G_G) - \#_l(G_G)}{\#_l(G_G)}\right) \times 100, \tag{15}$$

where $\#_n(G)$ (resp. $\#_l(G)$) is the number of nodes (reps. links) in graph G. We used this error to find the set of parameters from each reconstruction algorithm that gives the best reconstructed graph based on a grid search of the parameters with the following ranges:

DBScan Parameters ranges:

v_{th}: {18, 20, 22},
R: {0.1, 0.15, 0.2, 0.25, 0.3},
N: {5, 10, 15, 20, 25}.

DBScanRec Parameters ranges:

v_{th}: {16, 18, 20, 22, 24},
maxClustNpts: {50, 100, 150},
Rmax: {0.2, 0.3, 0.4},
Rstep: {0.025, 0.05},
Rmin: {0.05, 0.1, 0.15},
Nmin: {5, 10, 15},
Nstep: {5},
Nmax: {20, 25, 30}.

For both algorithm, we chose to reconstruct the nodes as polygons, but we did not filter on node size (min size = 0, max size = $+\infty$).
The graphs corresponding to the optimal set of parameters for each algorithm on 100 independent simulations generated by the procedure described in subsection 5.2 with the network from Fig.S8D are presented in Table S7. The recursive DBScan algorithm performs much better than the DBScan version (8.1% error vs 28.4%). This performance is associated with our error that measures the matched nodes and links only and not the amount of nodes and links in general. The parameters for the DBScan algorithm were: $v_{th} = 20$ μm/s, $R = 0.1$ μm, $N = 5$ and for the DBScanRec algoritfm: $v_{th} = 16$ μm/s, $R_{min} = 0.1$ μm, $R_{max} = 0.3$ μm, $R_{step} = 0.025$ μm, $N_{min} = 5$, $N_{max} = 20$, $N_{step} = 5$, $maxClustNpts = 50$. The other parameters were identical between the two algorithms: node type: polygon, min. area = 0 μm^2, max. area = 999999999 μm^2.

6 Dispersion index to measure the local exploration of the ER nodes by trajectories

To investigate how trajectories disperse when exiting an ER node, we synchronize them on their last point spent in node n. Trajectories are described as $X_i = \{X_i(t_j)\}$ where $j = 1 \ldots M_i$ is the number of points in trajectory X_i.

For each trajectory X_i passing through the node n, we define its synchronization time t_i^* to be its last point inside n: $X_i(t_i^*) \in n$ and $X_i(t_{i^*+1}) \notin n$. The dispersion index is computed for each node n on the ensemble of synchronized trajectories:

$$I_n(k) = \frac{1}{N_{n,k}} \sum_{i,j}^{i>j} ||X_i(t_{i^*+k}) - X_j(t_{j^*+k})||, \tag{16}$$

where $N_{n,k}$ is the number of unique pairs that can be formed from the remaining synchronised trajectories $k > 0$ frames after exiting node n and $||.||$ is the Euclidean norm.

7 Local trajectory exploration of the ER under dATL mutation

Analysis of SPTs obtained from lumen in mutant COS-7 dATL cells reveal a change in the network organization (Fig. S8H) characterized by an extended rectilinear motion, suggesting that trajectories are now restricted in tubules that are of ≈ 5 μm in length.

7.1 Long tubule detections from SPTs

We use the following method to detect long tubules from individual trajectories recorded inside the ER. This method is based on the assumption that these trajectories should be very elongated. Considering an ensemble of N trajectories $\{X_i, i = 1, .., N\}$ such that $X_i = \{X_i(t_j), j = 1, .., M_i\}$, the algorithm is as follows:

1. We remove trajectories with less that th_{traj_size} points.

2. To estimate the amount of elongation of trajectories, we fit an ellipse $\varepsilon_i = (\boldsymbol{\mu}_i, a_i, b_i, \varphi_i)$, where $\boldsymbol{\mu}$ is the ellipse center, a, b the large (resp. small) semi-axes lengths and φ the orientation, around each trajectory X_i, $i = 1..N$ using the minimum volume ellipsoid method.

3. We keep trajectories X_i such that the semi-axes ratio $th_{traj\_rat\_min} < \frac{a_i}{b_i} \leq th_{traj\_rat\_max}$.

4. We then compute the bounding square s_i of the ellipse ε_i of width $2a_i$, length $2b_i$, orientation φ_i and center $\boldsymbol{\mu}_i$.

5. We then seek to form groups of overlapping trajectories with similar orientations. Considering the set of already processed trajectories P, we form a new group of trajectories G as follows:

   (a) Start with the next unprocessed trajectory i, add it to P and G: $P = P \cup \{i\}$, $G = G \cup \{i\}$.

   (b) While G has changed since the last iteration, we do the following update:

      i. For each trajectory $j \in G$:

         A. Search any unprocessed trajectory $k \notin P$

         B. if the area of the overlap between the bounding square of the two trajectories $A_{j,k} = \text{area}(s_j \cap s_k)$ such that $A_{j,k} > th_{area\_rat} \times \min(\text{area}(s_j), \text{area}(s_k))$ and the angle between the two squares is $< th_{sq\_angle}$, then add k to G and P: $G = G \cup \{k\}$, $P = P \cup \{k\}$.

   (c) The resulting group is composed of overlapping trajectories with similar orientations that approximates well the observed long tubules.

6. Finally, we filter out groups with $< th_{group\_size}$ trajectories, and similarly to trajectories, we fit an ellipse $\varepsilon_g = (\boldsymbol{\mu}_g, a_g, b_g, \varphi_g)$ around all the trajectories in the group using the minimum volume ellipsoid method and filter out groups for which the ratio of semi-axes lengths $r_g = \frac{a_g}{b_g} < th_{group\_rat}$.

The parameters used for the above method are: $th_{traj\_size} = 5$, $th_{traj\_rat\_min} = 4$, $th_{traj\_rat\_max} = 40$, $th_{area\_rat} = 0.2$, $th_{sq\_angle} = \frac{\pi}{12}$, $th_{group\_size} = 5$ and $th_{group\_rat} = 11$.

8 SPT Acquisition: summary of experimental protocols

The acquisition setup for the trajectories presented in Fig.2 (Main Text) and Figs.S2, S4, S5 are described as follows: the CaV2.1 splice variants (+47 / Δ47) were N-terminally tagged with mEOS3.2 and the corresponding plasmids were transfected at 4 DIV in cultures of dissociated primary hippocampal neurons. The acquisition were performed at 15-19 DIV using an inverted TIRF setup on an eclipse Ti microscope (Nikon GmbH) equipped with a 100 x Apo TIRF oil objective (1.49 NA; Nikon). Fluorescence was

excited by oblique illumination of the probe with a combined laser system (Coherent; MPB communications Inc.) and image sequences (up to 9999 frames) were captured by an EMCCD camera (iXon+ 897, Andor Technology) controlled by NIS-Elements Advanced Research acquisition software (Nikon) at a frame rate of 50 Hz.

The trajectories presented in Figs. 3,4,7 (Main Text) and Fig.S7,S8H were acquired in a similar setup. Briefly, vectors encoding ER-targeted N-terminal preprotrypsin signal sequence and a C-terminal retrieval signal HaloTag (used for COS-7 dATL experiments), or HaloTag calreticulin (used for COS-7 WT and HEK293 experiments) were labelled 24 h after transfection with 0.5 nM cell-permeant HaloTag-TMR ligand (Promega G8251) for 10 min, followed by 3 washes with label-free medium; and imaged with 18 ms exposure on an Elyra Super Resolution microscope (Zeiss) using an α-Plan Apochromat x100 oil, 1.46 NA objective.

Finally, the trajectories presented in Fig. 5,6 (Main Text) were acquired in COS-7 cells expressing EGFP-VAPA (ER marker) and were stained with SiR-Lysosome and imaged every 1.5 sec on a custom-built SIM microscope using a 60x/1.2 numerical aperture water immersion lens (UPLSAPO 60XW, Olympus).

9 Performance evaluation

In this section, we asses the performances of our algorithms as implemented in the ImageJ plugin. For evaluating the loading time and diffusion map generation, we generated increasingly larger sets of 100, 500, 1000, 5000, 10000, 50000 and 100000 trajectories each possessing 10 points. The computation time is evaluated in a Jython script ran through ImageJ by subtracting the time after and before N applications of corresponding procedure and dividing the resulting value by N.

For trajectories loading and diffusion maps $N = 100$, while for the well detection and network reconstruction $N = 10$. Finally the values reported in Fig. S9 are the average $\pm$ standard deviation over 10 repeats of this procedure and the results are presented as a log-log plot. We can see that our algorithm have overall exponential complexities. The trajectory loading (Fig. S9A) is quite fast, taking less than one second in the worst case. The diffusion map reconstruction is also relatively fast (Fig. S9B) and is not much affected by the application of filtering or the choice of parameters. The well detection time (Fig.S9C) can get much longer for larger datasets, of the order of minutes, and we can see small differences between the algorithms,

the density being the fastest and the drift being much slower than the other two for small datasets. Finally, the graph reconstruction time (Fig. S9D) is the longest process that can take up to dozens of minutes for the largest datasets with the recursive DBScan procedure being much slower than the BDScan one.

10 Practical guidelines for parameters selection

As a first general principle, many parameters used in the Plugin have physical units (e.g. length) and can be directly related to the acquired data.

10.1 Map construction

All maps rely on an underlying grid where the only parameter is the bin size Δx (in nanometer). Δx should be chosen large enough to maximise the number of points in each bin but small enough to still be able to resolve the local variations. The drift and diffusion maps also posses another parameter, minPts the minimum number of displacements in a bin ensureing that enough displacements are considered for the local averages. We advise to use minPts=10 when possible, but if data are sparse, Ndisps=5 or Ndisps=3 can eventually be used but this will increase the variability in the estimations.

10.2 Well detection

Our well detection algorithms rely on the construction of a density map to find the high-density regions. We found that in practice using a filter size $k > 1$ (proposed options are k=1,3,5,7) in combination with a small grid bin (around 50 nm) and small threshold value ($\sim$0.05%) gives the best results. The threshold directly influences the computation time of the method. The other parameters are specific to the different types of detection algorithms:

Hybrid and multiscale hybrid algorithms: A grid is required, this grid influences the size of a region increased at each iteration. Here we advise using the multiscale algorithm that tries multiple different bin sizes. Another key parameter in these methods is the confidence ellipse percentage where we advise to use 95% by default, but 99% can be used for deep wells and 90% for shallow ones. Finally, maxSize controls the number of iterations done by the algorithms, and prevents detecting wells that are too big and should be set to the maximal size acceptable for the wells. The other parameters such

as “minPtsTh”, “angSimTh”, “sampledRatioTh”, “minSlicePtsTh” filter out elements with not enough data points and should be set more or less conservatively depending on the amount of trajectories and size of the wells.

Density algorithm: Another grid is constructed at each high-density region in order to refine the position of the well center. It is controlled by the parameters “Local grid bins width” and “Local grid size”. We advise to choose the bin width to be smaller than the one used for the density grid and the grid size small enough so that it does not overlap with another local maximum. The ring parameters (min, max, width) should be chosen to obtain a good approximation of the local point density distribution, we advise the width to be around or smaller than the local bin width and the maximum value depends on the approximate wells sizes. Finally, the “Max. cov. ratio dist” parameter controls where to stop when searching for the circle with the highest covariance ratio, for this parameters you should make sure that this distance will lie inside your well otherwise it can lead to some artifacts.

10.3 Graph reconstruction

The velocity threshold v_{th} influences the runtime of the algorithm as it directly modifies the number of points considered in the clustering. To set this parameter, we advise to consider the spatial distribution of the instantaneous velocities: for that goal, in the plugin interface, the user should go to the “Display option” menu, making sure “Trajectories” is ticked, then in the color category of the “Trajectory Option” section choose “InstVel”, that will color the trajectories’s displacements depending on their instantaneous velocity. From here, the distribution of velocities can be obtained by clicking on the “Hist” button next to the “Trajectories” tickbox and combine it with the “Max. Inst. vel” field to filter out displacements with instantaneous velocities above the selected value.
For the other parameters, we advise to first start in “DBScan” mode and play with “R” and “N” until finding values that correctly define your clusters. Then if there are some clusters that need to be splitted, switch to “DBScanRecursive” where you can enter ranges centered around the values for R and N found with DBScan. The parameter “Max. cluster pts.” is used to control when to stop splitting a cluster, and depends on the amount of values needed in the clusters. For the node shape, we advise keeping the “POLY” option which computes the cluster boundary as the convex hull polygon.
