# Supplementary material for: High-throughput super-resolution single-particle trajectory analysis reconstructs organelle dynamics and membrane reorganization
Source: Cell Rep Methods. 2022 Aug 22;2(8):100277. doi: 10.1016/j.crmeth.2022.100277 (PMC9421586; doi:10.1016/j.crmeth.2022.100277)
Supplement: Document S2. Article plus supplemental information [file mmc4.pdf]

Cell Reports
Methods

Article

High-throughput super-resolution single-particle trajectory analysis reconstructs organelle dynamics and membrane reorganization

Graphical abstract

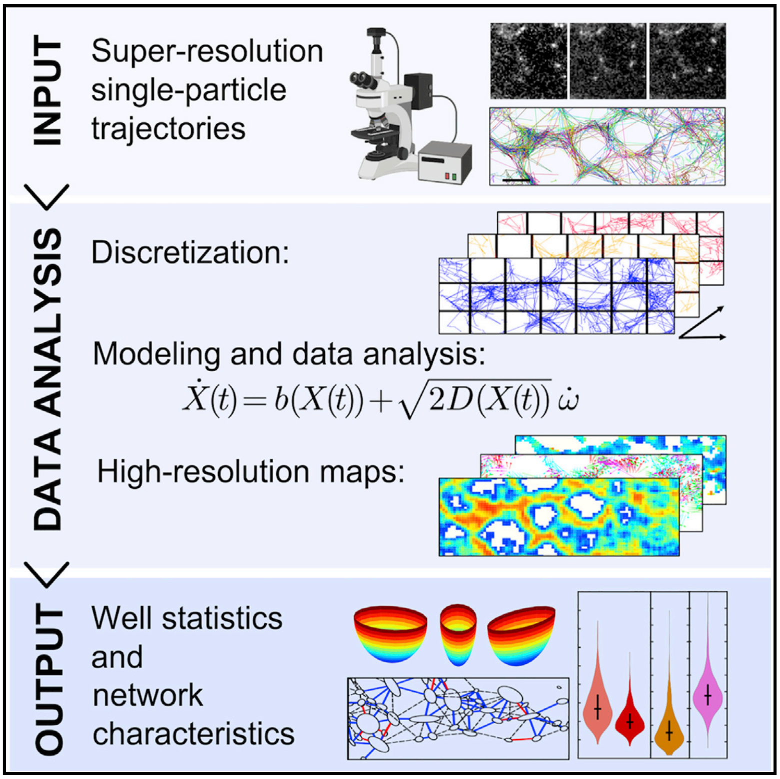

Highlights

- Algorithms to extract statistics from super-resolution single-particle trajectories
- Extracting local binding, trafficking interactions and membrane organization in neurons
- Reconstructing the endoplasmic reticulum (ER) network from trajectories
- Diffusion maps and quantifying the interactions between lysosomes and the ER

Authors

Pierre Parutto, Jennifer Heck, Meng Lu, Clemens Kaminski, Edward Avezov, Martin Heine, David Holcman

Correspondence

david.holcman@ens.fr

In brief

The rapid development of super-resolution single-particle trajectories microscopy requires high-throughput analysis techniques. Parutto et al. propose methods to reconstruct and quantify organelle networks and membrane reorganization from trajectories of any molecules, receptors, or channels of interest that can be tagged. The methods are available as an ImageJ plugin (SPTAnalysis).

Parutto et al., 2022, Cell Reports Methods 2, 100277
August 22, 2022 © 2022 The Author(s).
https://doi.org/10.1016/j.crmeth.2022.100277

CellPress

Cell Reports Methods

CellPress
OPEN ACCESS

Article

High-throughput super-resolution single-particle trajectory analysis reconstructs organelle dynamics and membrane reorganization

Pierre Parutto,[1,6] Jennifer Heck,[2,7] Meng Lu,[3] Clemens Kaminski,[3] Edward Avezov,[4] Martin Heine,[2] and David Holcman[1,5,8,*]

[1]Group of Data Modeling and Computational Biology, IBENS, Ecole Normale Supérieure, 75005 Paris, France
[2]Research Group Functional Neurobiology at the Institute of Developmental Biology and Neurobiology, Johannes Gutenberg University Mainz, Mainz, Germany
[3]Department of Chemical Engineering and Biotechnology, University of Cambridge, Cambridge CB3 0AS, UK
[4]UK Dementia Research Institute at the University of Cambridge and Department of Clinical Neurosciences, University of Cambridge, Cambridge CB2 0AH, UK
[5]DAMPT, University of Cambridge, DAMPT and Churchill College, Cambridge CB30DS, UK
[6]Present address: UK Dementia Research Institute at the University of Cambridge and Department of Clinical Neurosciences, University of Cambridge, Cambridge CB2 0AH, UK
[7]Present address: Genome Biology Unit, European Molecular Biology Laboratory (EMBL), Heidelberg, Germany
[8]Lead contact
*Correspondence: david.holcman@ens.fr

https://doi.org/10.1016/j.crmeth.2022.100277

MOTIVATION Classifying the type of molecular motion and extracting the underlying bio-physical and geometrical parameters from large amount single-particle trajectories remains a daunting task. We present methods and algorithms available as an ImageJ plugin called "TrajectoryAnalysis" that allow users to automatically detect regions of high densities, compute the diffusion and density maps with high precision, find the boundary and energy of the nanodomains defined as potential wells, and reconstruct a network from trajectories.

SUMMARY

Super-resolution imaging can generate thousands of single-particle trajectories. These data can potentially reconstruct subcellular organization and dynamics, as well as measure disease-linked changes. However, computational methods that can derive quantitative information from such massive datasets are currently lacking. We present data analysis and algorithms that are broadly applicable to reveal local binding and trafficking interactions and organization of dynamic subcellular sites. We applied this analysis to the endoplasmic reticulum and neuronal membrane. The method is based on spatiotemporal segmentation that explores data at multiple levels and detects the architecture and boundaries of high-density regions in areas measuring hundreds of nanometers. By connecting dense regions, we reconstructed the network topology of the endoplasmic reticulum (ER), as well as molecular flow redistribution and the local space explored by trajectories. The presented methods are available as an ImageJ plugin that can be applied to large datasets of overlapping trajectories offering a standard of single-particle trajectory (SPT) metrics.

INTRODUCTION

Subcellular compartments are focused sites where large numbers of molecules dynamically interact to support cellular function (Cole et al., 1996). The trajectories of ions and proteins as they move between the cytoplasm, plasma membrane, and organelles are critical to cellular function (Betzig et al., 2006; Cohen et al., 2018). These dynamics occur at the endoplasmic reticulum (ER) (Wu et al., 2017, 2018; Petkovic et al., 2014), the mitochondrial network, endosomes and lysosomes, and microtubules and are impacted by the local properties of these different environments (Lu et al., 2020). Several experimental paradigms measure these constitutive molecular motions at subcellular sites, including fluorescence recovery after photobleaching (FRAP) (Axelrod et al., 1976; Saxton and Jacobson, 1997), which locally depletes fluorescence and measures the timescale and fraction of recovery. Analysis of these data can reveal trafficking at a population level. In contrast,

This is an open access article under the CC BY license (http://creativecommons.org/licenses/by/4.0/).

photoactivation (Wang et al., 2014) consists of activating molecules in a local region of the cell and reveals their spread over a transient time frame. Combined with diffusion modeling and stochastic simulations, various biophysical properties can be measured, including diffusion coefficients and the fraction and timescale of recovery (Lippincott-Schwartz et al., 2003). These methods provide information on the dynamic function of organelles but are insufficient to identify and reconstruct high-density regions. These approaches also cannot examine phase separation stability or the local spaces explored by molecules at a nanoscale resolution. Statistical analysis (Manley et al., 2008; Hoze et al., 2012) of a large ensemble of super-resolution single-particle trajectories (SPTs) (Figures 1A and 1B) has the potential to reveal local molecular interactions. Molecules are not uniformly distributed inside a cell but instead form heterogeneous aggregates, possibly in phase-separated nanodomains, characterized by high-density regions (HDRs). Such regions are characterized by reduced velocity movement of molecules and confinement. These local areas can be enriched with calcium channels at neuronal synapses and store-operated calcium entry receptors such as STIM1 on spine apparatus and can also be found at ER nodes (Heck et al., 2019; Wu et al., 2014; Heine and Holcman, 2020; Holcman et al., 2018). Interestingly, these ubiquitous structures are transient yet persist, with a timescale that is longer than that associated with molecular trafficking. In short, many interactions that are critical to the cell are regulated and controlled by subcellular mechanisms that currently cannot be easily captured by quantitative analysis.

To determine the underlying physical properties of molecular trafficking, various computational modeling methods have been developed to analyze SPTs. These models include those based on classical free (Crank, 1979) and confined (Kusumi et al., 1993; Saxton, 1995; Saxton and Jacobson, 1997; Holcman and Schuss, 2004) diffusion, active deterministic motion, or a mixture of deterministic and stochastic models (Hozé and Holcman, 2017). Based on this theoretical framework, analysis of SPTs has revealed the dynamics of local chromatin organization in the nucleus (Gasser, 2016; Amitai and Holcman, 2017; Hauer et al., 2017), synaptic receptor trafficking at neuronal synapses (Dupuis and Groc, 2020), and vesicular stomatitis virus G protein (VSVG) virus assembly (Manley et al., 2008). A significant recent advance is the analysis of massive numbers of overlapping SPTs. This statistical analyses can reveal the properties of molecular trajectories. However, analyzing these data can potentially also allow quantification of membrane dynamics (Holcman et al., 2015) and may give insight into organelle organization.

Software developed to analyze SPTs generally fall into two categories: (1) those where parameters are extracted along individual trajectories, and (2) those based on spatially combining these trajectories to recover intrinsic properties. The first category includes SpotOn (Hansen et al., 2018), used to fit multi-states diffusion models from the distribution of displacements, the 4P-parameter algorithm, used to reconstruct chromatin dynamic (Amitai et al., 2017; Shukron et al., 2019), and, more recently, the 4P-Gaussian-Mixture (Basu et al., 2021), used to subsegment trajectories into confined and unconfined regions. For the second category, common methods include diffusion/drift maps and potential well extractions pioneered in Hoze et al. (2012) (https://bionewmetrics.org/super-resolution-single-particle-trajectories-using-stochastic-analysis/) or the SR-Tesseler algorithm (Levet et al., 2015), used to reconstruct and extract biophysical parameters from the local density of points. Another package is TRamWAy (Beheiry et al., 2015), used to reconstruct diffusion and energy maps, but this approach does not reconstruct the potential well boundaries.

Here, we have developed a method based on hybrid algorithms and automated an analysis pipeline for SPTs (Figure 1C) based on the Langevin (Schuss, 2009) equation of motion to provide statistical analysis of these data. This method estimates biophysical characteristics and is capable of reconstructing nanodomain sizes and boundaries using the classical physical model of a potential well (Kramers, 1940; Schuss, 2009), well known since Kramer's work in 1940 (Kramers, 1940). This method allowed us to characterize calcium channel organization in the membranes of hippocampal neurons. We also present an algorithm that reconstructs a network from SPTs based on the clustering of low-velocity trajectory fragments and use it to reconstruct ER network topology as well as estimate the timescale of lysosome trafficking and ER network interactions. Finally, these methods allowed us to extract the motion of trajectories relative to the reconstructed network, thereby revealing the redistribution of trajectories inside the ER of normal and atlastin-null mutant cells (Niu et al., 2019). The diversity of datasets used here demonstrate the broad applicability of these methods to cell biological processes. The algorithms we developed here are available in an ImageJ plugin with a graphical interface for processing individual experiments and a programming interface for batch processing.

RESULTS

Cosine filtering improves diffusion map accuracy

The diffusion coefficient is extracted from SPTs locally and is used to construct the diffusion map. Such maps have been previously constructed from a square grid overlaid on top of the trajectories (Hoze et al., 2012; Heck et al., 2019), but this estimation is strongly affected by the number of displacements falling in each bin. To improve the accuracy of the diffusion map, we propose to use a cosine filter that computes for each bin of a square grid the diffusion coefficient based on all the displacements starting within some radius of the bin center (larger than the bin size). Each displacement is weighted by a cosine function depending on its distance to the center of the bin (see Method details).

We tested the capacity of the method to reconstruct a synthetic diffusion field, either with a constant or with local variations using numerical simulations (see Methods S1). We find that the cosine-filter method has a smaller overall error and much greater coverage than the classical diffusion map (Figure S1). We then compared the results obtained by raw diffusion map, cosine filtering, and local averaging on empirical data (Figure S2; Data S1) and found that they all give similar results, but cosine filtering recovers more local details compared with the other methods.

Algorithm to reconstruct nanodomains in HDRs

In order to extract subcellular regions where a high density of molecular interactions are occurring, we developed a novel

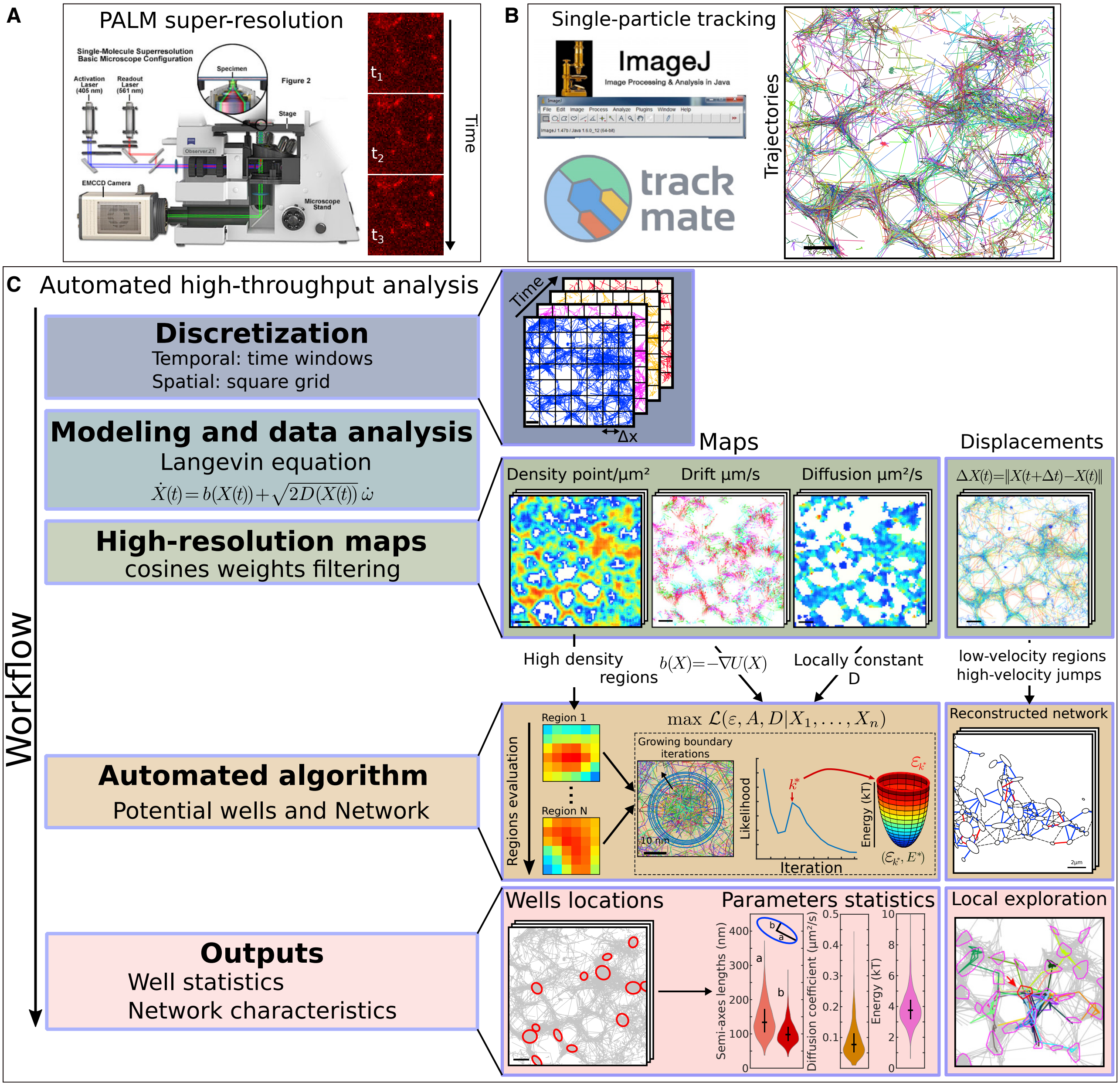

Figure 1. High-throughput SPT analysis pipeline

Figure360▷ For a Figure360 author presentation of this figure, see https://doi.org/10.1016/j.crmeth.2022.100277.

High-throughput automated trajectory analysis workflow.

(A) Acquisition device and raw data of a single-particle experiment.

(B) Raw data from (A) are transformed into trajectories using classical software such as Trackmate, available as an ImageJ plugin.

(C) Schematic description of the high-throughput analysis implemented here: trajectories are first discretized both spatially, using a square grid, and temporally, using temporal binning (time-windows analysis). We then interpret the trajectories based on the Langevin equation, allowing us to generate high-resolution maps of the local trajectories motion. High-density/low-velocity regions of the maps are extracted by automated algorithms to detect potential wells/reconstruct the network. Finally, the outputs consist in statitstics associated with well locations and with reconstructed network. These characteristics allow to analyze how trajectories locally explore their environment at the nanometer scale.

computational approach and automated pipeline (Figure 1) based on stochastic equations, multi-scale analysis, optimal estimators, and maximum likelihood statistics. In contrast to methods used to extract density of points (Sibarita, 2014; Levet et al., 2015) or to compute the maximum likelihood estimator (MLE) (Parutto et al., 2019; Briane et al., 2020), the present approach combines density of points with local dynamics associated with the elementary displacement $\Delta \boldsymbol{X} = \boldsymbol{X}(t+\Delta t) - \boldsymbol{X}(t)$, where $\boldsymbol{X}(t)$ is the

position of the particle at time t (Hoze et al., 2012; Heck et al., 2019) to extract the field of forces. Indeed, trajectories following the stochastic Equation (2), where the forces defining the nanodomain given by Equation (12) are characterized by a local accumulation approximated by a Gaussian density of point, and converging arrows for the local drift field (Figures S3Aa–S3Ac; Data S1). We could thereby assess the nature, organization, and stability of a large amount of HDRs by collecting statistics that can reveal hidden cellular organization. HDRs could previously only be characterized by manual curation based on extracting parameters of potential wells, a concept in classical physics (Chandrasekhar, 1943; Schuss, 2009) that describes the stability of dynamic systems, such as the motion of a bead attached to a spring. In contrast, using combined optimization procedures, the present method allows users to automatically extract local diffusion coefficients, energetics, local field potential, and, most importantly, local boundaries.

The method relies on an interesting observation that nanodomains of high density tend to have an elliptic shape. Thus, the first step to detect them is to recover their center and boundaries (see Method details). Non-automated classification algorithms have used the density of points (Parutto et al., 2019) or displacements (ΔX) separately, but these procedures often lead to parameter estimations that are not completely satisfactory due to a shallow minimum of the associated error function that leads to a large variability and possible mistakes in the estimation of most of the nanodomain parameters such as the boundary and the energy of nanodomains.

To overcome these difficulties, we developed a hybrid algorithm (Figure 1), described in the Method details, which combines two independent procedures starting with a principal-component analysis to recover the elliptic boundary and followed by a maximum likelihood estimation of the effective diffusion coefficient and drift properties. More precisely, the new algorithm comprises three steps:

1. Automatic determination of bins with the highest density of points (Figure S3Ba).
2. For each such bin, we iterate over square regions of increasing width w_k around the bin center. For each iteration k, we apply a principal-component analysis to estimate the semi-axis a_k, b_k, the center c_k, and a score $\mathcal{L}$ (Equation 24) based on the points falling in the square of size w_k (Figures S3Bb and S3Bc). We iterate until we reach the maximum size of the well set by the user (Figure S3Bc).
3. In the termination step, we select the optimal value of the iteration providing the optimal parameter (see Method details).

The reconstruction is illustrated in Figure S3C for three wells. To evaluate the performances of this hybrid algorithm, we constructed ground-truth datasets consisting of trajectories following Equation (2), with the energy field given by 12 for different types of potential wells (see Methods S1 for the detail of the procedures). In order to be able to compare different algorithms in a fair manner, we developed a parameter optimization procedure where a grid search is used to find the parameters of each algorithm that gives most accurate reconstruction of a known potential well. Based on this procedure, we showed that the hybrid algorithm performs better than two previous algorithms, the drift-based algorithm from Heck et al. (2019) and the density-based algorithm from Parutto et al. (2019), in estimating all parameters, especially at higher well energies (see Tables S1 and S2). The algorithm is effective at reconstructing wells over a large range of energies, from 1 to 10 kT (Table S3; Figure S3D), and can effectively discriminate the presence or absence of a parabolic field (see also Methods S1 for detection on wells with $E = 0$ kT). Moreover, for sufficiently large regions, the algorithm can distinguish between a parabolic field and a Brownian motion confined by impenetrable walls (see Methods S1 and Data S1).

At this stage, we have thus validated the hybrid algorithm by using ground-truth datasets to identify and automatically reconstruct nanodomains. This hybrid algorithm outperformed the others when reconstructing the exact shape of the nanodomain and its energy.

Analysis of SPTs for the endogeneous voltage-gated calcium channels reveals their organization and weaker stability in nanodomains

We applied the new hybrid algorithm to reanalyze trajectories of calcium voltage channels (CaV2.1) on the surface of neuronal cells for two overexpressed splice variants, CaV2.1 Δ47 and CaV2.1 +47, previously shown to shape synaptic short-term plasticity (Heck et al., 2019), as well a new set of endogenously tagged CaV2.1 channels. Using a large number of redundant SPTs, we were able to automatically detect the nanodomains defined as HDRs. Representative examples of such trajectories and their associated density and diffusion maps are presented in Figures 2A–2C. The algorithm allowed us to identify the characteristics of the nanodomains (reported in Table S4) approximated as ellipses with semi-axis lengths $a = 143 \pm 51$ and $b = 104 \pm 33$ nm for Cav2.1 Δ47. These parameters are similar for Cav2.1 +47, while they were smaller for endogenous Cav2.1 with $a = 100 \pm 47$ and $b = 73 \pm 31$ nm (Figure 2D). The diffusion coefficient was $D = 0.091 \pm 0.052\ \mu m^2/s$ for the Δ47 variant and $D = 0.087 \pm 0.045\ \mu m^2/s$ for the +47 variant and was smaller for endogenous CaV2.1, with $D = 0.069 \pm 0.051\ \mu m^2/s$ (Figure 2E). Interestingly although the associated energy was $\sim 3.9\ kT$ for the two variants and 3.3 kT for endogenous CaV2.1 (Figures 2F and 2G), we found that the associated residence times of a receptor in a nanodomain was around 174–181 and 94 ms, respectively (Figure 2H). The estimations of these residence times are larger than the ones reported in Heck et al. (2019), which could be due in part to differences in the boundary estimated by the present algorithm and in part due to the different estimators used for computing the attraction and diffusion coefficients of potential wells. Indeed, the estimators constructed from the statistical moments used in Heck et al. (2019) have a tendency to underestimate these parameters, whereas the MLEs used here give values closer to the ground truth (see Tables S1–S3 and Methods S1). Interestingly, we found a positive correlation between the diffusion coefficients and the sizes as well as the attraction coefficients A but no correlation with the energy of the potential wells (Figures S6A–S6C; Methods S1). Finally, we conclude that the present approach captures the differences between the variant forms of the calcium channels,

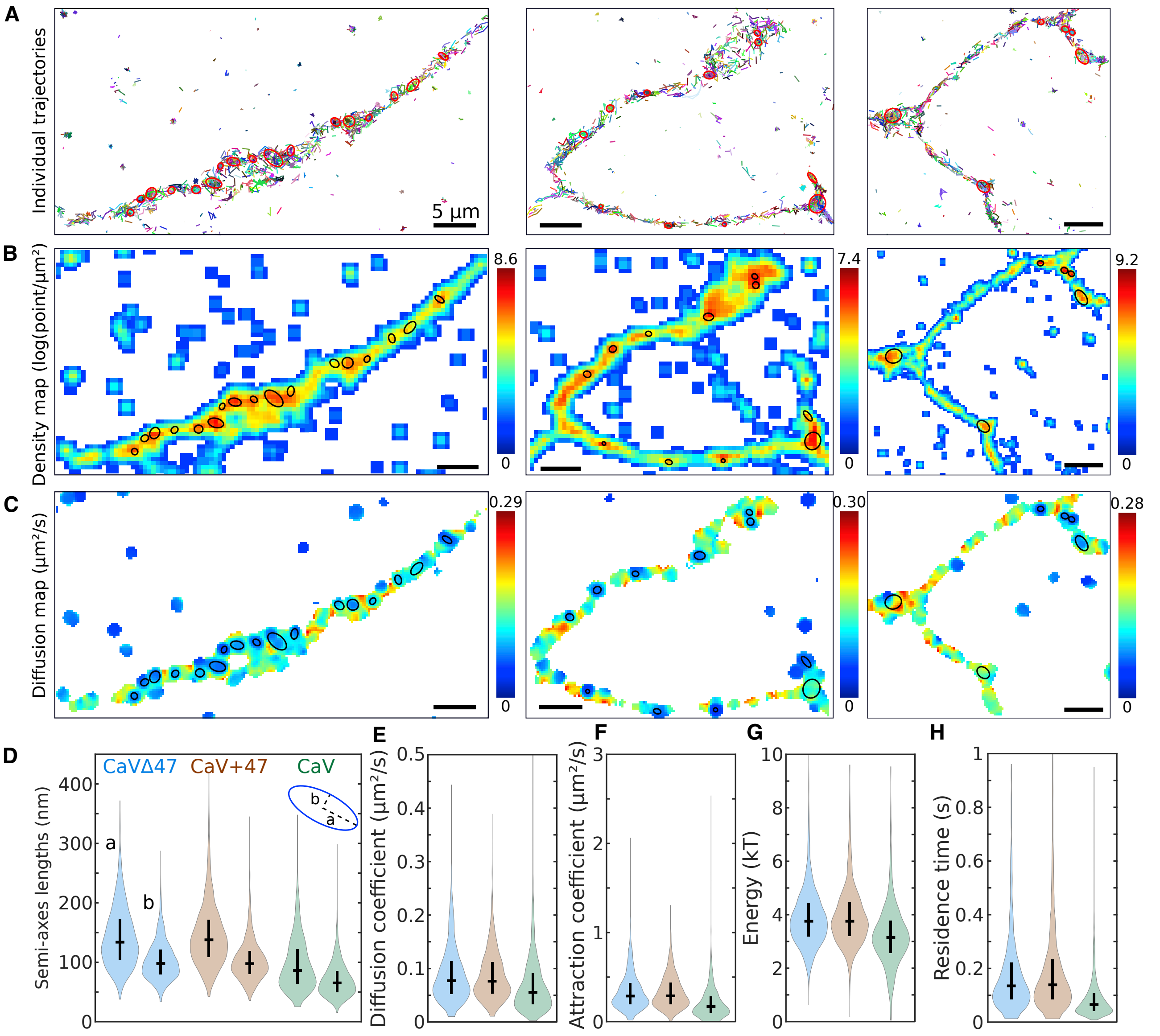

Figure 2. Automated large statistics of CaV nanodomains located on dendrites

Super-resolution SPTs automated analysis reveals CaV nanodomain organization.

(A) Examples of endogenous CaV2.1 trajectories together with the detected potential well regions (red ellipses).(B) Associated density map, presenting the local point density (in log (points/μm^2)) and computed over a grid with bin sizes of 50 nm and locally averaged over a 3 × 3 Gaussian kernel.

(C) Associated cosine-filtered diffusion maps (see cosine filtering improves diffusion map accuracy), displaying the local diffusion coefficients (in $\mu m^2/$ s) computed over a grid with bin sizes Δx = 80 nm, for bins possessing at least 15 displacements and using a disk radius r_{filt} = 100 nm.

(D–H) Violin plots representing the distribution, median, and interquartile range of the well characteristics recovered using the hybrid algorithm from CaV2.1 Δ 47, CaV2.1 +47, or endogenous CaV2.1 trajectories. (D) Distribution of semi-axes lengths of elliptic well boundaries, (E) diffusion coefficients inside wells, (F) attraction coefficients *A*, (G) energies of the well (kT), and (H) residence time distribution of a trajectory inside a well.

See also Tables S4 and S5.

revealing a significant reduction in interactions for the endogenous dataset (see Methods S1).

Time-lapse analysis reveals the stability of CaV nanodomains over time

To investigate the stability of nanodomains across time, we used a time-lapse analysis (Figure S4; Data S1) with sliding windows of 20 s and no overlap. This analysis allows determination of the lifetime of a nanodomain, which is given by the number of successive windows where it is detected (Figure S5A; Data S1). For example, the trajectories obtained during a 250 s experiment are split into 13 20 s windows (0–20, 20–40, …, 240–260 s). We searched for the presence of potential wells in each window (Figures S5A and S5B). To follow

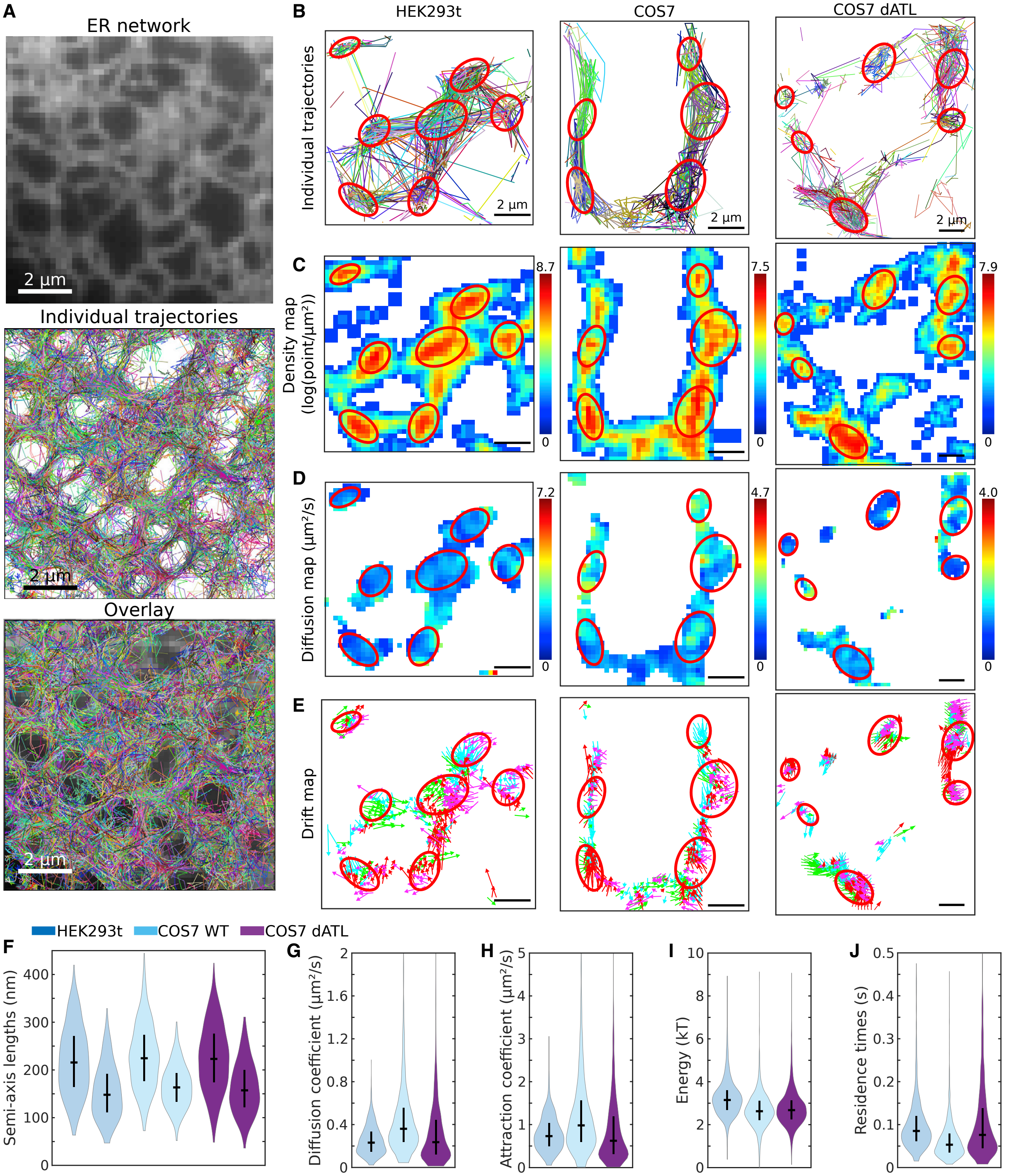

Figure 3. HDR present in organelle such as the ER network characterized as potential wells

(A) ER network geometry (top), associated luminal trajectories (middle), and overlay (bottom).

(B) Individually color-coded trajectories recorded inside the ER for three different cells (HEK293t, COS-7, COS7-dATL). Red ellipses represent detected potential wells.

(legend continued on next page)

a well across successive time windows, we consider that two wells identified at times t_k and t_{k+1} are the same if the distance between their centers is less than 200 nm. The ensemble of consecutive times $(t_q, \ldots, t_r)$, where a well is first detected at time t_q and disappears at time t_{r+1}, is used to define the stability duration $\tau = t_r - t_q$. This analysis allows us to follow the size of the small and large axes of the wells and the associated energy over time (Figures S5C and S5D). Finally, we found that ~55% of the wells were present for more than 20 s (one time window) and that their average duration is ~56 s for both Δ47 and +47 variants (Figure S5E; Table S5). However, nanodomain stability is reduced to 46 s for endogenous CaV2.1. All of these durations are longer than the ~30 s that we previously reported (Heck et al., 2019), indicating that the present algorithm advances our ability to capture the dynamics of these high-density enigmatic subcellular domains.

The hybrid algorithm reveals that some ER nodes are nanodomains defined by an attracting field of force

To further explore the range of applicability of the present hybrid algorithm, we analyzed SPTs recorded from an ER luminal probe in COS-7 wild-type (WT) and HEK-293T cells, both presented in Holcman et al. (2018) and in COS-7 atlastin knockout (dATL) cells obtained as in Holcman et al. (2018) (see Methods S1). The ER networks of these cells generally form a flat tubular monolayer at their periphery (Schroeder et al., 2019), allowing the two-dimensional tracking of individual molecules as shown in Holcman et al. (2018). In the dATL mutant, the morphology of peripheral ER tubules is altered, but it is unclear how the ER flow is affected. Since nodes have been previously characterized as HDRs (Holcman et al., 2018), we asked here whether these nanodomains could further be defined as potential wells. Applying the hybrid algorithm reveals several potential wells (Figures 3A and 3B, red ellipses) precisely located at nodes forming HDRs. We further estimated the density, diffusion and drift maps, observing converging arrows patterns in these regions (Figures 3D and 3E, and S6D), a classical feature of potential wells (Hozé and Holcman, 2017). Interestingly, these HDRs were characterized by ellipses with large semi-axis lengths ~220 nm, , small semi-axis lengths ~160 nm, diffusion coefficients $\sim 0.3\ \mu m^2/s$, and attraction coefficients $\sim 1\ \mu m^2/s$, corresponding to energies $\sim 2.9\ kT$ and residence times ~ 90 ms (Figures 3F–3J; Table S6). Interestingly, although the elliptic parameters are not much different in the case of COS-7 WT and COS-7 dATL, a difference can be observed in the dynamical parameters characterizing the transport of the material across the ER network. To conclude, the present hybrid algorithm reveals that some ER nodes concentrate trafficking of luminal molecules by a spring-force type mechanism, the origin of which should be further explored.

Organelle network reconstruction from a large number of SPTs

We next wanted to check whether our approach can be used to define the structure of the ER and be generalized to other organelles. For these goals, we developed a novel method and algorithm to reconstruct the network from SPTs.

Graph reconstruction algorithm (GRA) to unravel the ER network

Although SPTs can be used to explore the ER network architecture (Holcman et al., 2018), we still lacked a method for the automated reconstruction of the ER, especially in cases where the local density is variable. We therefore developed an algorithm to see if the dynamic architecture of this complex organelle can be recovered from SPTs. We illustrate the principle of the automatic ER-reconstruction procedure in Figure S7 and (Methods S1) for ER-luminal trajectories. The starting point is to color trajectory displacements depending on their instantaneous velocity, which reveals a dynamical segregation of the ER into nodes and tubules (Figures S7A–S7C). Based on this segregation, we developed the GRA to recover the ER structure from SPTs (see Methods S1).

This procedure allows us to reconstruct a two-dimensional graph for the organelle network that can be used to study further statistical properties. Finally, we tested the GRA on a ground-truth dataset exacted from a live-cell image (Figure S8; Methods S1). We simulated trajectories on the extracted graph and applied our algorithm on these trajectories to reconstruct the topology of the underlying ER network. This procedure gave a satisfactory reconstruction result (8.1% error), thus validating the present algorithm (Table S7).

GRA of the ER network of COS-7 dATL cells reveals aberrant organization and trafficking

Next, we examined whether disruptions to organelle structure and dynamics can be captured using our algorithm. We analyzed SPTs recorded in the ER of COS-7 dATL cells (acquired as in Holcman et al. [2018]; see Methods S1), which lack the ER membrane-shaping protein atlastin (double knockout of the ATL2/3 genes [Niu et al., 2019]) and exhibit a disrupted peripheral ER morphology with elongated tubules (Niu et al., 2019). We present color-coded trajectories based on their instantaneous velocity (Figures 4A and 4B) and the density and diffusion maps (Figures 4C and 4D), as well as the histogram of the apparent diffusion coefficients (Figure 4E), revealing an average $D_{app} = 1.56 \pm 0.83\ \mu m^2/s$.

We applied the GRA to obtain a network from these trajectories (Figure 4F) where the HDRs (brown) are connected by blue segments. This approach allows the quantification of the distribution of distances between nodes with a mean $d_{nodes} = 0.92 \pm 0.32\ \mu m$ (Figure 4G) and the nodes area $A_{nodes} = 0.15 \pm 0.12\ \mu m^2$ (Figure 4H). Note that the GRA could miss non-explored ER regions or regions that are underrepresented in the trajectories. To conclude, our algorithm allows us

(C–E) Density, diffusion, and drift maps for the regions shown in (A). Arrows in the drift maps are colored according to the direction: West (purple), East (green), South (blue), and North (red).
(F–J) Violin plots representing the distribution, median, and interquartile range of the characteristics of the detected potential wells. (F) Semi-axis lengths (large a and small b), (G) diffusion coefficient D, (H) attraction coefficient A, (I) energy of the well E, and (J) estimated residence time.
See also Table S6.

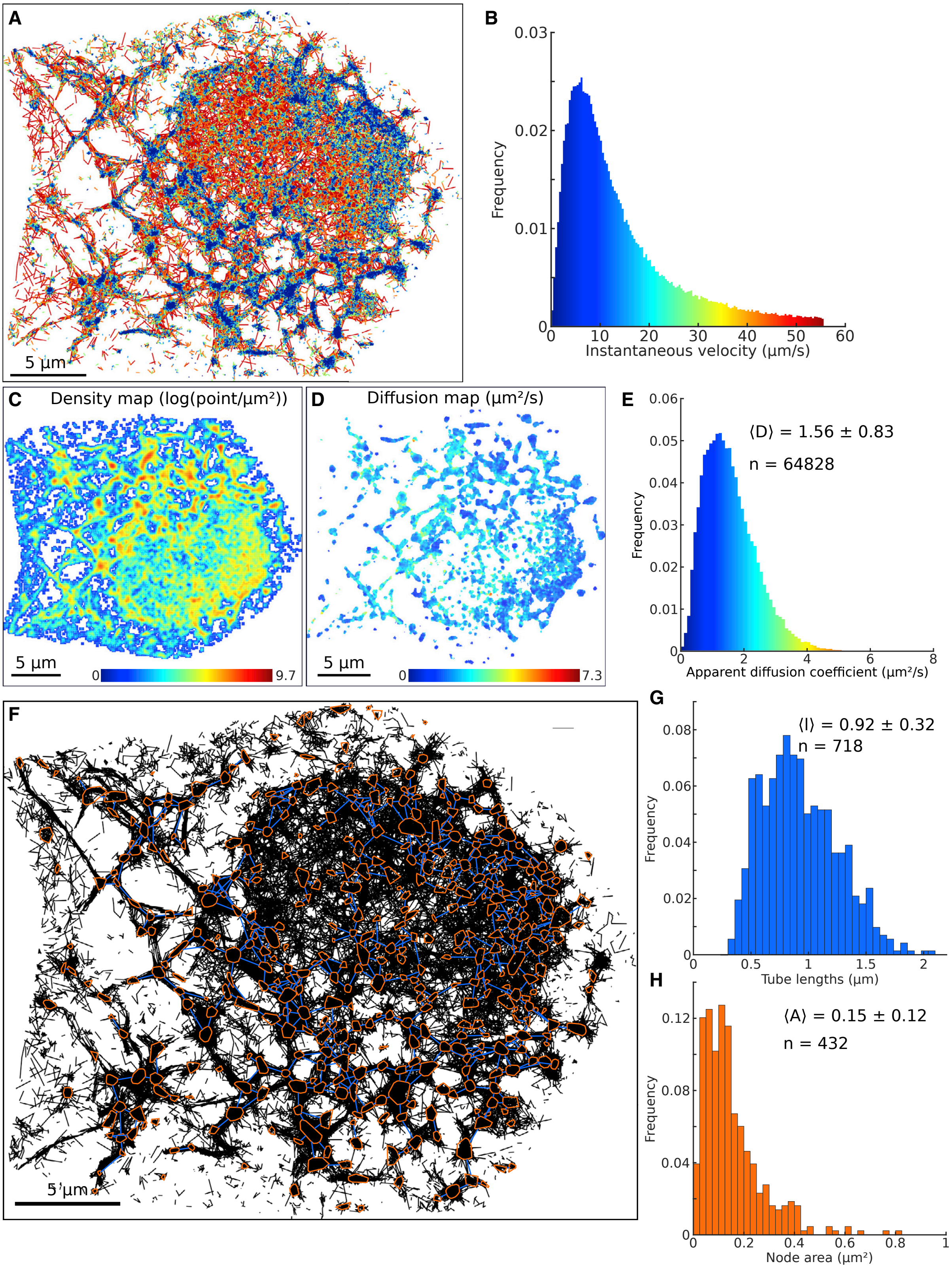

(legend on next page)

to automatically reconstruct organelle networks based on SPT exploration once there are enough datapoints.

GRA and SPT segmentation reveal the duration of ER-lysosome interactions

To demonstrate the broad applicability of the algorithms, we analyzed an ensemble of trajectories of individual lysosomes obtained in COS-7 cells from Lu et al. (2020) (Figure 5A). These trajectories are characterized by a distribution of instantaneous velocities in the range $[0-3.5]$ $\mu m/s$ (Figure 5B). However, low and high velocities are not segregated, as was the case for ER luminal trajectories, but are instead found in similar regions (Figure 5A, left and right). The distribution $f(v)$ of velocities (Figure 5B) can be fitted by a sum of two exponentials:

$$f(v) = A\exp\left(-\frac{v}{v_0}\right) + B\exp\left(-\frac{v}{v_1}\right), \qquad \text{(Equation 1)}$$

where a best fit approximation gives $v_0 = 0.043$ $\mu m/s$ (95% confidence interval $[0.041, 0.044]$) and $v_1 = 0.388$ $\mu m/s$ (95% confidence interval $[0.370, 0.407]$) (coefficient of determination $R^2 = 0.999$), with $A = 0.780$ and $B = 0.075$. This fit suggests that the distribution of lysosomes is largely driven by low-velocity components. The rare appearance of high-velocity components suggests a possible switch between slow and fast motions. Finally, note that 86.1% of displacements are associated with a velocity of less than 0.5 $\mu m/s$, and 12.7% are in the range of [0.5–1.5] $\mu m/s$.

To further study how lysosomes move in the cytoplasm, we computed relevant density and diffusion maps (Figures 5C and 5D) and found that the motion had a diffusion component (Figure 5E), with an average apparent diffusion coefficient of $D_{app} = 0.062 \pm 0.040$ $\mu m^2/s$. Interestingly, regions of low-diffusion coefficients colocalized with regions of high density in the density map (Hoze and Holcman, 2014; Holcman et al., 2015) (Figures 5A–5C).

We then isolated regions of high density using a method based on the density of points (Method details), revealing an ensemble of n = 95 HDR subdomains, approximated by ellipses (black in Figure 5F) of semi-axis lengths $a = 516 \pm 196$ (large) and $b = 278 \pm 143$ nm (small) (Figure 5G). By considering the displacements connecting different regions, we reconstructed (Method details) a network explored by the lysosomes (Figure 5H), where HDRs (red circles) are connected by direct lines (yellow). Interestingly, the histogram of average velocities between these regions is not symmetric (Figure 5I), with a mean velocity $v = 1.03 \pm 0.32$ $\mu m/s$, which clearly deviates from diffusion, as computed from the Rayleigh distribution. This deviation suggests that the transitions between these regions are driven by an active motion. Moreover, the overlay between ER (white) and the lysosome reconstructed network (Figure 5H) suggests that the lysosome trajectories follow the topology of the ER network (Lu et al., 2020). To conclude, our analysis reveals that lysosomes travel along a network that strongly colocalizes with the ER. However, high and low velocities occur in similar regions. Since lysosomes move along microtubules, this present statistical analysis suggests that the ER-microtubule network shapes lysosome trafficking.

Trajectory resynchronization approach reveals single local molecular dynamic exploration inside an ensemble

In the previous result sections, we reconstructed the networks hidden inside SPT data. We shall now introduce a last step in our method, which resynchronizes trajectories that fall inside the same subcellular area but that were acquired at random times. This approach allows us to study the dynamics of trajectories with respect to the ensemble of trajectories that visit the same spatial region. The approach also enables determination of the local spatiotemporal properties that trajectories explore at the single-unit level.

Trajectory segmentation reveals ER-lysosomes interaction timescale

To study the possible interactions between lysosomes and the ER (Figure 6A), we focused on the confined portion found along individual lysosome trajectories (see Method details and Figure 6A). We hypothesize that the lysosome motion can switch between a directed and confined motion (Figure 6B). We first show that the lysosome can indeed switch between directed and confined motion (Figure 6C).

To recover the size of the confinement areas, we fitted ellipses over these regions and obtained average semi-axes lengths (Figure 6D) of $a = 232 \pm 77$ (large) and $b = 94 \pm 47$ nm (small). Furthermore, this approach allowed us to estimate the confinement strength λ by considering that the confined motion could be generated by a spring force, modeled by an Ornstein-Uhlenbeck process (Schuss, 2009). We found that the spring force is $\lambda = 0.123 \pm 0.025$ s^{-1} (Figure 6E), associated with an average local diffusion coefficient of $D = 0.032 \pm 0.020$ $\mu m^2/s$ (Figure 6F) for a total of n = 818 confinement regions. Finally, the distribution of times in confined regions could be well approximated by a single exponential with a time constant $\tau = 5.35$ s (Figure 6G). The average residence time of lysosomes in these regions is $\bar{\tau} = 30 \pm 12$ s, which can be interpreted as a time where lysosomes could interact with the ER. These confinement events are quite common, with trajectories spending ~30% of their lifetime confined (Figure 6H) in one or multiple confinement events (Figure 6I). To conclude, the present algorithm reveals that as lysosomes travel along a network that strongly colocalizes with the

Figure 4. ER network reconstructed in a COS-7 dATL cell
(A and B) Individual trajectories color coded according to their instantaneous velocity shown in (B).
(C and D) Corresponding density (C) and drift (D) maps.
(E) Distribution of diffusion coefficients obtained from the individual bins of the diffusion map presented in (D), with the average ± SD (n is the number of bins).
(F) Reconstructed network, showing the nodes in red and links in blue overlaid on top of the individual trajectories (black), with the average ± SD (n is the number of links).
(G) Distribution of distances (i.e., tubule lengths) between connected nodes, with the average ± SD (n is the number of links) .
(H) Distribution of the areas covered by nodes, with the average ± SD (n is the number of nodes).

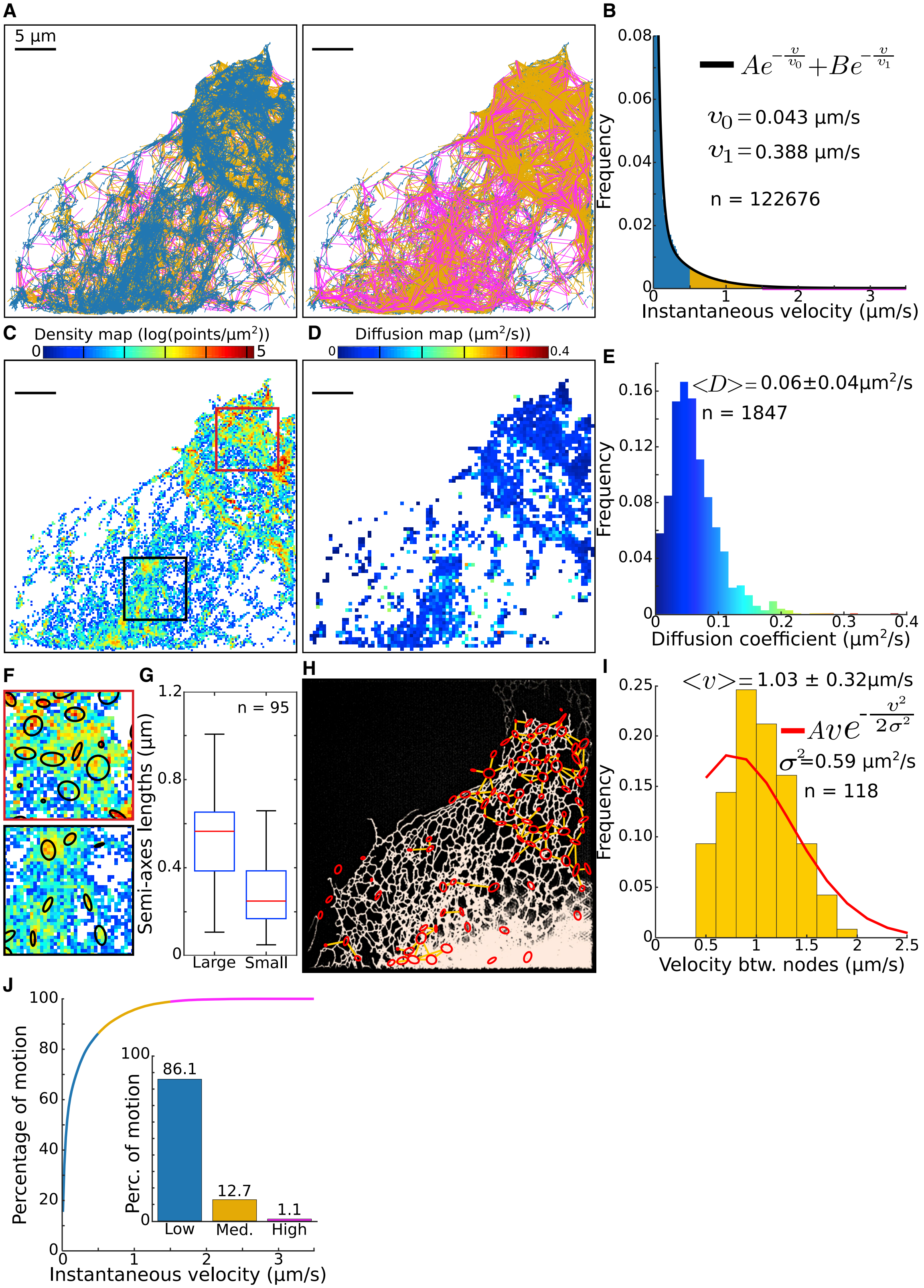

(legend on next page)

ER, the velocity can switch from large to small displacements, and the trajectories can become restricted into regions of size ~200 nm, on a timescale of 5 s. This could correspond to a change in directionality of movement or a direct interaction with the ER. Our approach can therefore extract changes in lysosome dynamics that may reflect functional interactions from complex data.

Trajectory resynchronization approach shows how a single trajectory explores single nodes

After an ER network is reconstructed from SPTs by the GRA (Figure 7A), the node-tubule topology emerges. Thus, it becomes possible to study how trajectories locally explore the network by synchronizing them upon exit from a chosen node (Figure 7B). Interestingly, we found that the mean instantaneous velocity at exit is $v_e = 30.2 \pm 10.2$ μm/s and keeps decreasing during the next 200 to 300 ms (Figure 7B). Escape occurs in equal directions (Figures 7C and 7D), as shown in four examples where we followed their dispersal. To characterize this dispersion, we plotted the dispersion index (as defined in Methods S1), revealing two phases (Figure 7B): one below 100 ms, showing a rapid dispersion, followed by a second phase with less expansion. These two phases can be interpreted as follows: for the first one, trajectories escape from a well, then in the second phase, trajectories tend to be recaptured for a certain time in nodes, thus preventing a fast exploration of the network.

Trajectory resynchronization reveals novel local dynamics within dATL ER-tubules

We next analyzed how the local space exploration of trajectories is modified in COS-7 dATL cells (Figure S8H). Under normal conditions, trajectories are mostly located in nodes (Holcman et al., 2018), while here trajectories predominantly explore long tubules (Figure S8I) with average lengths of 5.4 ± 2.44 μm (Figure S8J), much longer than the ~ 1 μm found for the tubules of WT cells. Inside these tubules, we found that trajectories exhibit a "stuttering" behavior around different positions that lasts for seconds. To characterize this behavior, we estimated several parameters such as the duration that a trajectory spent around a given position $\tau_{//} = 79 \pm 76$ ms (Figure S8K), the transition time between different positions $\tau_t = 27 \pm 15$ ms (Figure S8L), the length of a transition step $\Delta_{//} = 0.53 \pm 0.45$ μm (Figure S8M) and finally the SD around the stable positions SD = 0.14 ± 0.07 μm (Figure S8N).

To conclude, following the reconstruction of networks using our algorithm, we were able to reposition and resynchronize SPTs. Using analyses of the ER lysosome, ER in normal COS-7 cells, and ER in COS-7 dATL cells, the algorithm revealed trajectories explored by the local space and the associated time scales.

DISCUSSION AND LIMITATIONS OF THE METHODS

We present here a general method and the associated algorithms that can automatically characterize nanodomains where trajectories accumulate. Our approach generates graph representations of organelle networks from SPTs. Automatically finding nanodomains is useful to extract large statistics (size, energy of potential wells, mean residence time of particles) and compare their properties. Further, by quantifying the trajectories inside and outside these nanodomains, we could recover membrane organization, as well as determine the local redistribution dynamics of organelles and proteins. By reconstructing a graph of ER or lysosome networks from SPTs, we can recover molecular flow at the nanoscale level. We found here that nanodomains could be characterized as an attractive region (potential wells), and this generic representation suggests a universal mechanism of molecular stabilization that probably requires further investigation. Interestingly, these structures can be transiently remodeled in time, as revealed by the present time-lapse analysis.

Universality of high-density nanoregions characterized as potential wells

High-density nanoregions have now been associated with potential wells for several receptors and channels such as CaV (Heck et al., 2019), AMPAR (Hoze et al., 2012), glycine receptors (Masson et al., 2014), or GAGs (Hoze and Holcman, 2017; Floderer et al., 2018). Interestingly, some nodes of the ER can also be characterized as potential wells, which may reflect retention of luminal content or could be interpreted as a nanoregion allowing protein maturation. This representation suggests a generic membrane organization to retain particles (receptors, channels, proteins, etc.) in a field of force with long-range interactions.

Interestingly, the geometry of these regions and their energy profiles are independent of the experimental conditions, further confirming again their stability. Note that the physical nature of the potential well remains unclear (Holcman, 2013). The present method could be applied to analyze molecular crowding and the dynamics of nanodomains, thus clarifying processes relevant for phase separation at synapses (Feng et al., 2019). With the

Figure 5. Lysosome trajectories analysis

(A and B) Individual lysosome trajectories displacements color coded according to their instantaneous velocity shown in (B). The black line in (B) corresponds to a fit to a two-exponentials function (n is the number of displacements).

(C and D) Corresponding density (C) and diffusion (D) maps.

(E) Histogram of diffusion coefficients obtained from the individual bins presented in (D), with the average ± SD (n is the number of bins).

(F) Magnification of the density map of two regions of interest presented in (C), showing high-density regions, approximated by ellipses.

(G) Boxplot showing the median, interquartile range, and extreme data points of the distributions of semi-axis lengths of the high-density regions approximated as ellipses.

(H) Reconstruction of a lysosome graph, where nodes (red ellipses) correspond to high-density regions and a link (in yellow) is added when at least one trajectory starts in one node and enter to the other one in one or two frames.

(I) Average instantaneous velocities between pairs of connected nodes presented in (H), with the average ± SD and a fit to a Rayleigh distribution (n is the number of velocities between nodes).

(J) Percentage of displacements with a specific instantaneous velocity. Inset, percentage of displacements for the velocity regimes defined in (A) and (B).

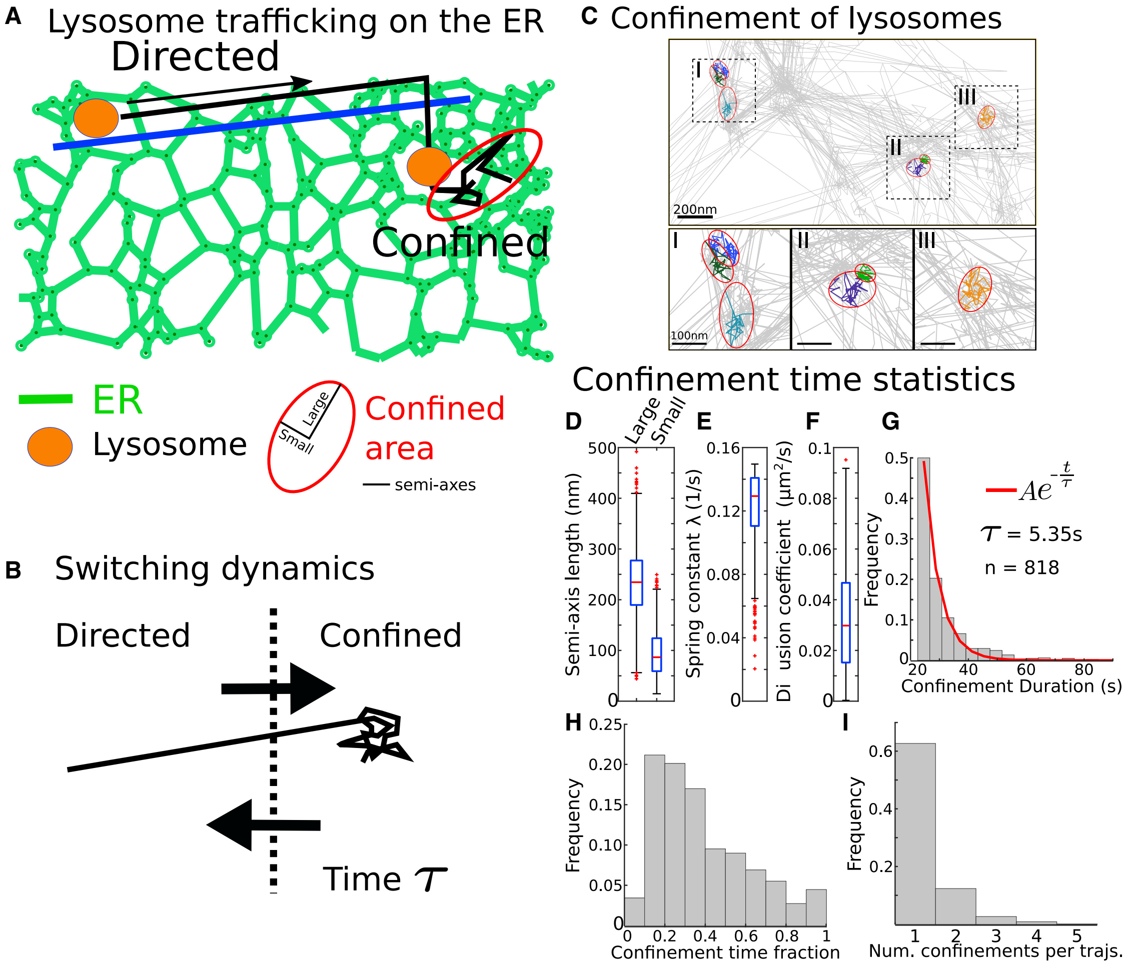

Figure 6. ER network-lysosome interaction revealed by confined trajectories

(A) Schematic representation of a lysosome switching between a directed motion along microtubule and confined motion at ER nodes.

(B) Switching dynamics representation: the confined state is characterized by a spring constant λ, a diffusion coefficient D, and a confined time constant τ.

(C) Three examples of confinement regions for different lysosome trajectories. Confined trajectories are colored, and unconfined trajectories are in gray.

(D–F) Boxplot showing the median, interquartile range, and extreme data points of the semi-axis lengths (small and large) of the ellipses fitted to the confinement regions (D), spring constants of the Ornstein-Ulenbeck process associated with the confinement regions (E), and diffusion coefficients estimated inside a confinement region (F). n = 818 confinement regions.

(G) Residence times inside a confinement region with a fit to a single exponential (n is the number of confinement regions).

(H) Fraction of time trajectories spend confined (relative to the trajectory length); n = 577 trajectories with at least one confinement event.

(I) Number of confinement events along individual trajectories; n = 521 trajectories with between 1 and 5 confinement events.

development of new labeling methods, improved fluorophores, and the ability to tag endogenous populations of molecules via CRISPR-Cas9, it will soon be possible to investigate phase separation at a population level and with SPT, to track endogenous dynamics, offering novel opportunities for the present approach.

Trafficking in networks

We show here that we can reconstruct a network from lysosome SPTs that resembles the ER network (Figure 5). This reconstructed network is further segregated into nodes and links, but low and high velocities are mixed (Figure 5A) compared with the reconstruction obtained from luminal proteins (Figure S7B). It is possible that lysosomes follow the cytoskeleton network, which is correlated with the ER (Lu et al., 2020). In addition, we find that the distribution of lysosome velocity follows a double exponential (Figure 5B) with fast ($\sim 0.388\ \mu m/s$) and slow ($\sim 0.043\ \mu m/s$) components. However, a more detailed analysis revealed that these velocities can be further subdivided into (1) confined motion (Figure 6) characterized by a residence time of ~ 5 s, and (2) deterministic motion between HDRs (Figures 6F and 6G), characterized by a distribution of velocity with an average of $1.03\ \mu m/s$. It would be interesting to better characterize the switch between confined and rectilinear motion. Regions of deterministic velocities and those where diffusion can be found are often not well separated, suggesting that lysosomes can use various modes of transport, independently of the subregions where there are located. We found,

CellPress
OPEN ACCESS

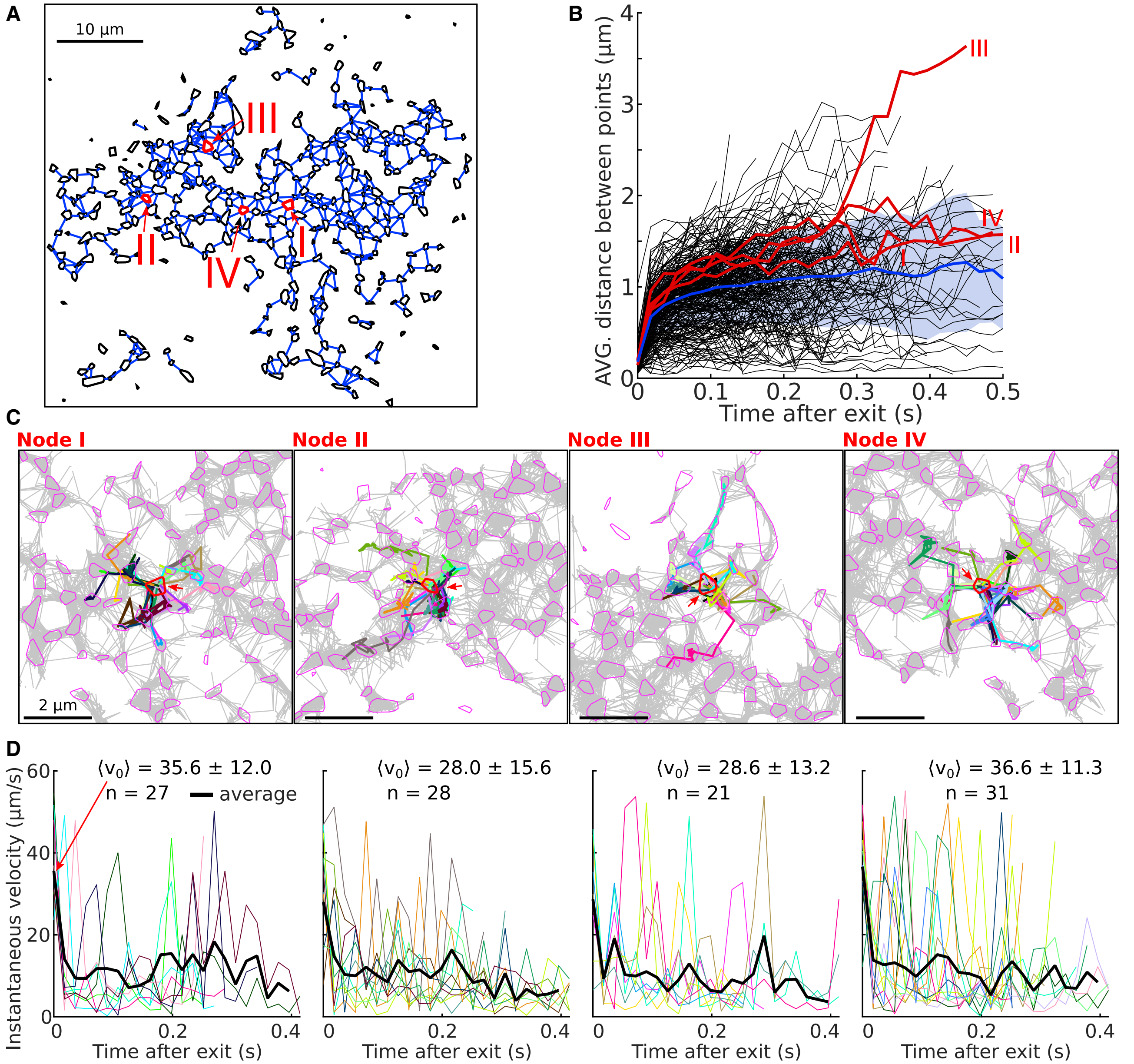

Figure 7. Local space exploration after trajectory resynchronization

Local network exploration revealed by spatial resynchronization of super-resolution SPTs.

(A) Reconstructed ER network using the GRA with nodes (black polygons) connected by segments (blue). Four nodes (I, II, III, and IV) are selected (red arrows).

(B) Average distance between the points of trajectories exiting a given node versus time after escape. The four nodes highlighted in (A) are also highlighted in red here, and the average line is presented in blue with its SD in blue shade (n = 258 nodes). This curve exhibits a fast (<50 ms) and a slow phase.

(C) Local exploration of neighboring nodes from trajectories (various colors) exiting from the chosen node for example nodes presented in (A).

(D) Distribution of velocities for trajectories exiting a node for the four nodes presented in (C). The rapid desynchronization of trajectories causes the mean velocity to rapidly decrease after exit (n is the number of trajectories exiting each node).

however, some regions characterized by a high density of trajectories, with a low velocity, suggesting that there are possible trapping mechanisms to retain lysosomes in specific subregions of the ER, possibly at exit sites (Manley et al., 2008). This mode of motion is quite different from the internal motion inside the ER lumen or on its membrane: in the first case, the node-tubule topology is associated with diffusion-drift while lysosomes may be trapped in interaction with the ER.

Future work should reveal interaction times between lysosomes and the ER. By applying our algorithms to different cells and organelles, we have shown that the boundaries and dynamics of subcellular interactions can be revealed from large

SPT datasets. The automated algorithms presented here can be applied to analyze hundreds of thousands of trajectories and to study nanodomains with almost no human interventions and are available as an ImageJ plugin.

STAR★METHODS

Detailed methods are provided in the online version of this paper and include the following:

- KEY RESOURCES TABLE
- RESOURCE AVAILABILITY
  - Lead contact
  - Materials availability
  - Data and code availability
- METHOD DETAILS
  - Diffusion model, velocity, vector fields and empirical estimators
  - Cosine-filtered estimation of the diffusion coefficient and drift
  - Local point density estimation
  - Estimating potential well parameters
  - Residence time of particles inside a well
  - Parabolic potential well representation
  - Recovering the center μ
  - Covariance matrix Σ
  - Confidence ellipse estimation $\varepsilon = (\mu, a, b, \varphi)$
  - Maximum likelihood estimators (MLE) based on an Ornstein-Uhlenbeck model
  - Hybrid density-drift algorithm
  - Fixed spatial scale hybrid density-drift algorithm
  - Multiscale hybrid algorithm
  - Density-based algorithm
  - Drift-based algorithm
  - Sliding window analysis to study the stability of the wells over time
  - Reconstructing the graph of the network explored by SPTs
  - Recursive velocity based graph reconstruction algorithm of a network explored by SPTs
  - Lysosome analysis
- QUANTIFICATION AND STATISTICAL ANALYSIS

SUPPLEMENTAL INFORMATION

Supplemental information can be found online at https://doi.org/10.1016/j.crmeth.2022.100277.

ACKNOWLEDGMENTS

D.H.'s research has received funding from the European Research Council (ERC) under the European Union's Horizon 2020 Research and Innovation Programme (grant agreement no. 882673), Plan Cancer-INSERM Projet19CS145-00, and ANR NEUC-0001. E.A. and P.P. are supported by the UK Dementia Research Institute, which receives its funding from UK DRI, Ltd., funded by the UK Medical Research Council, Alzheimer's Society, and Alzheimer's Research UK. J.H. is currently funded by the EMBL Interdisciplinary Postdoc Program (EIPOD) supported by the European Union's Horizon 2020 Research and Innovation Programme under Marie Skłodowska-Curie Actions (grant agreement no. 847543).

AUTHOR CONTRIBUTIONS

P.P. and D.H. conceived and designed the research plan, computational methods, and algorithms and wrote the manuscript. P.P. wrote the algorithms. M.H., E.A. and J.H. designed the experiments and collected super-resolution microscopy data.

DECLARATION OF INTERESTS

The authors declare no competing interests.

Received: December 3, 2021
Revised: May 11, 2022
Accepted: July 25, 2022
Published: August 22, 2022

REFERENCES

Amitai, A., and Holcman, D. (2017). Polymer physics of nuclear organization and function. Phys. Rep. *678*, 1–83.

Amitai, A., Seeber, A., Gasser, S.M., and Holcman, D. (2017). Visualization of chromatin decompaction and break site extrusion as predicted by statistical polymer modeling of single-locus trajectories. Cell Rep. *18*, 1200–1214.

Axelrod, D., Koppel, D.E., Schlessinger, J., Elson, E., and Webb, W.W. (1976). Mobility measurement by analysis of fluorescence photobleaching recovery kinetics. Biophys. J. *16*, 1055–1069.

Basu, S., Shukron, O., Hall, D., Parutto, P., Ponjavic, A., Shah, D., Boucher, W., Lando, D., Zhang, W., Reynolds, N., et al. (2021). Live-cell 3d single-molecule tracking reveals how nurd modulates enhancer dynamics. Preprint at Biorxiv. https://doi.org/10.1101/2020.04.03.003178.

El Beheiry, M., Dahan, M., and Masson, J.-B. (2015). Inferencemap: mapping of single-molecule dynamics with bayesian inference. Nat. Methods *12*, 594–595.

Betzig, E., Patterson, G.H., Sougrat, R., Lindwasser, O.W., Olenych, S., Bonifacino, J.S., Davidson, M.W., Lippincott-Schwartz, J., and Hess, H.F. (2006). Imaging intracellular fluorescent proteins at nanometer resolution. Science *313*, 1642–1645.

Briane, V., Vimond, M., and Kervrann, C. (2020). An overview of diffusion models for intracellular dynamics analysis. Brief. Bioinform. *21*, 1136–1150.

Chandrasekhar, S. (1943). Stochastic problems in physics and astronomy. Rev. Mod. Phys. *15*, 1–89.

Cohen, S., Valm, A.M., and Lippincott-Schwartz, J. (2018). Interacting organelles. Curr. Opin. Cell Biol. *53*, 84–91.

Cole, N.B., Smith, C.L., Sciaky, N., Terasaki, M., Edidin, M., and Lippincott-Schwartz, J. (1996). Diffusional mobility of golgi proteins in membranes of living cells. Science *273*, 797–801.

Crank, J. (1979). The Mathematics of Diffusion (Oxford University Press).

Dupuis, J.P., and Groc, L. (2020). Surface trafficking of neurotransmitter receptors: from cultured neurons to intact brain preparations. Neuropharmacology *169*, 107642.

Ester, M., Kriegel, H.-P., Sander, J., and Xu, X. (1996). A density-based algorithm for discovering clusters in large spatial databases with noise. In Proceedings of the Second International Conference on Knowledge Discovery and Data Mining, KDD'96 (AAAI Press), pp. 226–231.

Feng, Z., Chen, X., Zeng, M., and Zhang, M. (2019). Phase separation as a mechanism for assembling dynamic postsynaptic density signalling complexes. Curr. Opin. Neurobiol. *57*, 1–8.

Floderer, C., Masson, J.-B., Boilley, E., Georgeault, S., Merida, P., El Beheiry, M., Dahan, M., Roingeard, P., Sibarita, J.-B., Favard, C., and Muriaux, D. (2018). Single molecule localisation microscopy reveals how hiv-1 gag proteins sense membrane virus assembly sites in living host cd4 t cells. Sci. Rep. *8*, 17426–17515.

Friedrich, R., and Peinke, J. (1997). Description of a turbulent cascade by a fokker-planck equation. Phys. Rev. Lett. *78*, 863–866.

Gasser, S.M. (2016). Nuclear architecture: past and future tense. Trends Cell Biol. *26*, 473–475.

Hansen, A.S., Woringer, M., Grimm, J.B., Lavis, L.D., Tjian, R., and Darzacq, X. (2018). Robust model-based analysis of single-particle tracking experiments with spot-on. Elife *7*, e33125.

Hauer, M.H., Seeber, A., Singh, V., Thierry, R., Sack, R., Amitai, A., Kryzhanovska, M., Eglinger, J., Holcman, D., Owen-Hughes, T., and Gasser, S.M. (2017). Histone degradation in response to dna damage enhances chromatin dynamics and recombination rates. Nat. Struct. Mol. Biol. *24*, 99–107.

Heck, J., Parutto, P., Ciuraszkiewicz, A., Bikbaev, A., Freund, R., Mitlöhner, J., Andres-Alonso, M., Fejtova, A., Holcman, D., and Heine, M. (2019). Transient confinement of cav2. 1 ca2+-channel splice variants shapes synaptic short-term plasticity. Neuron *103*, 66–79.e12.

Heine, M., and Holcman, D. (2020). Asymmetry between pre-and postsynaptic transient nanodomains shapes neuronal communication. Trends Neurosci. *43*, 182–196.

Holcman, D. (2013). Unraveling novel features hidden in superresolution microscopy data. Commun. Integr. Biol. *6*, e23893.

Holcman, D., Hoze, N., and Schuss, Z. (2015). Analysis and interpretation of superresolution single-particle trajectories. Biophys. J. *109*, 1761–1771.

Holcman, D., Parutto, P., Chambers, J.E., Fantham, M., Young, L.J., Marciniak, S.J., Kaminski, C.F., Ron, D., and Avezov, E. (2018). Single particle trajectories reveal active endoplasmic reticulum luminal flow. Nat. Cell Biol. *20*, 1118–1125.

Holcman, D., and Schuss, Z. (2004). Escape through a small opening: receptor trafficking in a synaptic membrane. J. Stat. Phys. *117*, 975–1014.

Hoze, N., and Holcman, D. (2014). Residence times of receptors in dendritic spines analyzed by stochastic simulations in empirical domains. Biophys. J. *107*, 3008–3017.

Hoze, N., and Holcman, D. (2017). Coagulation-fragmentation with a finite number of particles: models, stochastic analysis, and applications to telomere clustering and viral capsid assembly. In Stochastic Processes, Multiscale Modeling, and Numerical Methods for Computational Cellular Biology (Springer), pp. 205–239.

Hozé, N., and Holcman, D. (2017). Statistical methods for large ensembles of super-resolution stochastic single particle trajectories in cell biology. Annu. Rev. Stat. Appl. *4*, 189–223.

Hoze, N., Nair, D., Hosy, E., Sieben, C., Manley, S., Herrmann, A., Sibarita, J.-B., Choquet, D., and Holcman, D. (2012). Heterogeneity of ampa receptor trafficking and molecular interactions revealed by superresolution analysis of live cell imaging. Proc. Natl. Acad. Sci. USA *109*, 17052–17057.

Kramers, H.A. (1940). Brownian motion in a field of force and the diffusion model of chemical reactions. Physica *7*, 284–304.

Kusumi, A., Sako, Y., and Yamamoto, M. (1993). Confined lateral diffusion of membrane receptors as studied by single particle tracking (nanovid microscopy). effects of calcium-induced differentiation in cultured epithelial cells. Biophys. J. *65*, 2021–2040.

Levet, F., Hosy, E., Kechkar, A., Butler, C., Beghin, A., Choquet, D., and Sibarita, J.-B. (2015). Sr-tesseler: a method to segment and quantify localization-based super-resolution microscopy data. Nat. Methods *12*, 1065–1071.

Lippincott-Schwartz, J., Altan-Bonnet, N., and Patterson, G.H. (2003). Photobleaching and photoactivation: following protein dynamics in living cells. Nat. Cell Biol., S7–S14.

Lu, M., van Tartwijk, F.W., Lin, J.Q., Nijenhuis, W., Parutto, P., Fantham, M., Christensen, C.N., Avezov, E., Holt, C.E., Tunnacliffe, A., et al. (2020). The structure and global distribution of the endoplasmic reticulum network are actively regulated by lysosomes. Sci. Adv. *6*, eabc7209.

Manley, S., Gillette, J.M., Patterson, G.H., Shroff, H., Hess, H.F., Betzig, E., and Lippincott-Schwartz, J. (2008). High-density mapping of single-molecule trajectories with photoactivated localization microscopy. Nat. Methods *5*, 155–157.

Masson, J.-B., Dionne, P., Salvatico, C., Renner, M., Specht, C.G., Triller, A., and Dahan, M. (2014). Mapping the energy and diffusion landscapes of membrane proteins at the cell surface using high-density single-molecule imaging and bayesian inference: application to the multiscale dynamics of glycine receptors in the neuronal membrane. Biophys. J. *106*, 74–83.

Niu, L., Ma, T., Yang, F., Yan, B., Tang, X., Yin, H., Wu, Q., Huang, Y., Yao, Z.-P., Wang, J., et al. (2019). Atlastin-mediated membrane tethering is critical for cargo mobility and exit from the endoplasmic reticulum. Proc. Natl. Acad. Sci. USA *116*, 14029–14038.

Parutto, P., Heck, J., Heine, M., and Holcman, D. (2019). Biophysics of high density nanometer regions extracted from super-resolution single particle trajectories: application to voltage-gated calcium channels and phospholipids. Sci. Rep. *9*, 18818–18914.

Petkovic, M., Jemaiel, A., Daste, F., Specht, C.G., Izeddin, I., Vorkel, D., Verbavatz, J.-M., Darzacq, X., Triller, A., Pfenninger, K.H., et al. (2014). The snare sec22b has a non-fusogenic function in plasma membrane expansion. Nat. Cell Biol. *16*, 434–444.

Saxton, M.J. (1995). Single-particle tracking: effects of corrals. Biophys. J. *69*, 389–398.

Saxton, M.J., and Jacobson, K. (1997). Single-particle tracking: applications to membrane dynamics. Annu. Rev. Biophys. Biomol. Struct. *26*, 373–399.

Schroeder, L.K., Barentine, A.E.S., Merta, H., Schweighofer, S., Zhang, Y., Baddeley, D., Bewersdorf, J., and Bahmanyar, S. (2019). Dynamic nanoscale morphology of the er surveyed by sted microscopy. J. Cell Biol. *218*, 83–96.

Schuss, Z. (2009). In Theory and applications of stochastic processes: an analytical approach, Schuss., ed. (Springer Science & Business Media).

Schuss, Z. (2010). Diffusion and Stochastic Processes: An Analytical Approach (Springer).

Shukron, O., Seeber, A., Amitai, A., and Holcman, D. (2019). Advances using single-particle trajectories to reconstruct chromatin organization and dynamics. Trends Genet. *35*, 685–705.

Sibarita, J.-B. (2014). High-density single-particle tracking: quantifying molecule organization and dynamics at the nanoscale. Histochem. Cell Biol. *141*, 587–595.

Siegert, S., Friedrich, R., and Peinke, J. (1998). Analysis of data sets of stochastic systems. Phys. Lett. *243*, 275–280.

Tang, C.Y., and Chen, S.X. (2009). Parameter estimation and bias correction for diffusion processes. J. Econom. *149*, 65–81.

Todd, M.J. (2016). Minimum-volume Ellipsoids: Theory and Algorithms (SIAM).

Wang, S., Moffitt, J.R., Dempsey, G.T., Xie, X.S., and Zhuang, X. (2014). Characterization and development of photoactivatable fluorescent proteins for single-molecule–based superresolution imaging. Proc. Natl. Acad. Sci. USA *111*, 8452–8457.

Wu, H., Carvalho, P., and Voeltz, G.K. (2018). Here, there, and everywhere: the importance of er membrane contact sites. Science *361*, eaan5835.

Wu, M.M., Covington, E.D., and Lewis, R.S. (2014). Single-molecule analysis of diffusion and trapping of stim1 and orai1 at endoplasmic reticulum–plasma membrane junctions. Mol. Biol. Cell *25*, 3672–3685.

Wu, Y., Whiteus, C., Xu, C.S., Hayworth, K.J., Weinberg, R.J., Hess, H.F., and De Camilli, P. (2017). Contacts between the endoplasmic reticulum and other membranes in neurons. Proc. Natl. Acad. Sci. USA *114*, E4859–E4867.

STAR★METHODS

KEY RESOURCES TABLE

REAGENT or RESOURCE	SOURCE	IDENTIFIER
Software and algorithms		
MATLAB2019b	mathworks	https://uk.mathworks.com/products/matlab.html
Python3.8.3	Python Software Foundation	https://www.python.org/
Fiji2.1.0	Open Source	https://fiji.sc/
SPTAnalysis1.1	This Paper	https://doi.org/10.5281/zenodo.6862643

RESOURCE AVAILABILITY

Lead contact

Further information and requests for resources should be directed to and will be fulfilled by the lead contact, David Holcman (david.holcman@ens.fr).

Materials availability

This study did not generate new unique reagents.

Data and code availability

- All data reported in this paper will be shared by the lead contact upon request.
- All original code has been deposited at github.com/holcman-lab/SPTAnalysis and is publicly available as of the date of publication. DOIs are listed in the key resources table.
- Any additional information required to reanalyze the data reported in this paper is available from the lead contact upon request.

METHOD DETAILS

Diffusion model, velocity, vector fields and empirical estimators

In the Smoluchowski's limit of the Langevin equation (Schuss, 2009), the position $\boldsymbol{X}(t)$ of a molecule is described by

$$\dot{\boldsymbol{X}} = \frac{F(\boldsymbol{X}(t),t)}{\gamma} + \sqrt{2D}\dot{W}, \qquad \text{(Equation 2)}$$

where $F(\boldsymbol{X})$ is a field of force, W is a white noise, γ is the friction coefficient (Schuss, 2009) and D is the diffusion coefficient. At a coarser spatio-temporal scale, the motion can be coarse-grained as a stochastic process (Hoze et al., 2012; Hoze and Holcman, 2014)

$$\dot{X} = a(\mathbf{X}) + \sqrt{2}B(\mathbf{X})\dot{W}, \qquad \text{(Equation 3)}$$

where $a(\boldsymbol{X})$ is the drift field and $B(\boldsymbol{X})$ the diffusion matrix. The effective diffusion tensor is given by $D(\boldsymbol{X}) = \frac{1}{2}B(\boldsymbol{X})B^T(\boldsymbol{X})$ ($.^T$ denotes the transposition) (Schuss, 2009, 2010). The drift and diffusion fields from Equation 3 can be recovered from SPTs acquired at any infinitesimal time step Δt by estimating the conditional moments of the trajectory displacements $\Delta\boldsymbol{X} = \boldsymbol{X}(t+\Delta t) - \boldsymbol{X}(t)$ (Schuss, 2009; Friedrich and Peinke, 1997; Siegert et al., 1998; Hoze and Holcman, 2014; Hozé and Holcman, 2017)

$$a(x) = \lim_{\Delta t\to 0}\frac{\mathbb{E}[\Delta\boldsymbol{X}(t)|\boldsymbol{X}(t)=x]}{\Delta t}, \qquad \text{(Equation 4)}$$

$$D(x) = \lim_{\Delta t\to 0}\frac{\mathbb{E}\left[\Delta\boldsymbol{X}(t)^T\Delta\boldsymbol{X}(t)|\boldsymbol{X}(t)=x\right]}{2\Delta t}. \qquad \text{(Equation 5)}$$

The notation $\mathbb{E}[\cdot|\boldsymbol{X}(t) = x]$ represents averaging over all trajectories that are passing at point x at time t. To estimate the local drift $a(\boldsymbol{X})$ and diffusion coefficients $D(\boldsymbol{X})$ at each point $\boldsymbol{X}$ of the membrane and at a fixed time resolution Δt, we use a procedure based on a square grid.

The local estimators to recover the vector field and diffusion tensor (Parutto et al., 2019) consist in grouping points of trajectories within a lattice of square bins $S(x_k, \Delta x)$ centered at x_k and of width Δx. For an ensemble of N two-dimensional trajectories $\{\boldsymbol{X}_i(t_j) = (x_i^{(1)}(t_j), x_i^{(2)}(t_j)),\ i = 1...N, j = 1...M_i\}$ with M_i the number of points in trajectory $\boldsymbol{X}_i$ and successive points recorded with an acquisition time $t_{j+1} - t_j = \Delta t$.,the discretization of Equation 4 for the drift $a(x_k) = (a^{(1)}(x_k),\ a^{(2)}(x_k))$ in a bin centered at position x_k is

$$a^{(u)}(x_k) \approx \frac{1}{N_k} \sum_{i=1}^{N} \sum_{j=0, x_i(t_j) \in S(x_k, \Delta x)}^{M_i - 1} \left(\frac{x_i^{(u)}(t_{j+1}) - x_i^{(u)}(t_j)}{\Delta t} \right), \qquad \text{(Equation 6)}$$

where $u = 1..2$ and N_k is the number of points $x_i(t_j)$ falling in the square $S(x_k, r)$. Similarly, the components of the effective diffusion tensor $D(x_k)$ are approximated by the empirical sums

$$D^{(u,v)}(x_k) \approx \frac{1}{N_k} \sum_{i=1}^{N} \sum_{j=0, \mathbf{X}_i(t_j) \in S(x_k, \Delta x)}^{M_i - 1} \frac{\left[x_i^{(u)}(t_{j+1}) - x_i^{(u)}(t_j)\right]\left[x_i^{(v)}(t_{j+1}) - x_i^{(v)}(t_j)\right]}{2\Delta t}. \qquad \text{(Equation 7)}$$

The centers of the bin and their size Δx are free parameters that are optimized during the estimation procedure.

Cosine-filtered estimation of the diffusion coefficient and drift

To increase the accuracy of the diffusion and drift maps, we weighted the points in the moving windows with a cosine function (would also be possible to use wavelets). In that case, the new estimator for the drift field is now

$$a^{(u)}(x_k) \approx \frac{\sum_{i=1}^{N} \sum_{j=0, x_j(t_j) \in \mathcal{D}(x_k, r)}^{M_i - 1} \left(\frac{\left(x_i^{(u)}(t_{j+1}) - x_i^{(u)}(t_j)\right)}{\Delta t} w_{i,j}(x_k, r) \right)}{\sum_{i=1}^{N} \sum_{j=0, \mathbf{x}_i(t_j) \in \mathcal{D}(x_k, r)}^{N_s - 1} w_{i,j}(x_k, r)} \qquad \text{(Equation 8)}$$

with N_k the number of points of the trajectories falling in the disk $\mathcal{D}(x_k, r)$ of radius r and centered at x_k. The weight of a displacement starting at $X_i(t_j)$ with respect to the disk $\mathcal{D}(x_k, \Delta x)$ is given by

$$w_{i,j}(x_k, r) = \cos\left(\frac{\pi}{2} \frac{\|\boldsymbol{X}_i(t_j) - x_k\|}{r} \right), \qquad \text{(Equation 9)}$$

with $\|.\|$ the Euclidean norm. In that case, we can choose a refined grid $S(x_k, (\Delta x)')$ with bin size $(\Delta x)' = \Delta x / l_{sc}$, where l_{sc} is a scaling factor. The role of the cosine weights w is to decrease continuously the influence of the points falling near the boundary.

Similarly, the generalized formula for the effective diffusion tensor $D(x_k)$ are given by the weighted sums

$$D^{(u,v)}(x_k) \approx \frac{\sum_{i=1}^{N} \sum_{j=0, \mathbf{X}_i(t_j) \in \mathcal{D}(x_k, r)}^{M_i - 1} \frac{\left(\left(x_i^{(u)}(t_{j+1}) - x_i^{(u)}(t_j)\right)\left(x_i^{(v)}(t_{j+1}) - x_i^{(v)}(t_j)\right)\right) w_{i,j}(x_k, r)}{2\Delta t}}{\sum_{i=1}^{N} \sum_{j=0, \mathbf{X}_i(t_j) \in \mathcal{D}(x_k, r)}^{M_i - 1} w_{i,j}(x_k, r)},$$

where the weights w are given by Equation (9).

Local point density estimation

The local density of points ρ can be determined using a procedure similar to the drift or diffusion estimation, dividing the image plane into a square bin $S(x_k, \Delta x)$. We then compute for each square of s centered at x_k

$$\rho_{\Delta x}(x_k) = \frac{N_k}{(\Delta x)^2}, \qquad \text{(Equation 10)}$$

where N_k is the number of trajectory points falling into the bin centered at x_k. In practice, it usually helps to smooth this density estimation by applying a local average using a small $d \times d$ kernel with $d \sim 1, 3, 5$.

Estimating potential well parameters

In this subsection, we present the estimators for the two main parameters of potential wells: the extent of their boundary and their associated energy (Hoze et al., 2012; Masson et al., 2014; Parutto et al., 2019). We recall that the diffusion coefficient inside a well is considered to be constant and the energy of the well given by $E = A/D$ where A is the attraction coefficient and D the diffusion coefficient. The correlations between potential wells characteristics for CaV2.1 and ER luminal data are presented in Figures S6A–S6C, S6E–S6G. In practice, for the potential wells reported in Figures 2 and 3, we filtered out the wells with energies $E > 10\ kT$.

Residence time of particles inside a well

The potential well model allows to estimate the residence time using the classical escape time formula (Schuss, 2009; Hoze et al., 2012) for a circular well

$$\tau_e \approx \frac{Dr^2}{4A^2} e^{\frac{A}{D}}, \quad \text{(Equation 11)}$$

with r the radius of the well, A its attraction coefficient and D its diffusion coefficient. In the case of an elliptic well, we obtain an approximate circular boundary using the harmonic mean of the semi-axes $r = \sqrt{ab}$, where a and b are the large and the small-axes lengths respectively. This approximation holds for $a \approx b$.

Parabolic potential well representation

To extract the energy of a potential well, we consider the basin of attraction of a truncated elliptic parabola with the associated energy function

$$U(\boldsymbol{X}) = \begin{cases} A\left[\left(\frac{x^{(1)} - \boldsymbol{\mu}^{(1)}}{a}\right)^2 + \left(\frac{x^{(2)} - \boldsymbol{\mu}^{(2)}}{b}\right)^2 - 1\right], & \boldsymbol{X} \in \mathcal{B} \\ 0 & \text{otherwise} \end{cases} \quad \text{(Equation 12)}$$

where $\boldsymbol{X} = [x^{(1)}, x^{(2)}]$, $\boldsymbol{\mu} = [\boldsymbol{\mu}^{(1)}, \boldsymbol{\mu}^{(2)}]$ is the center of the well, a, b are the elliptic semi-axes lengths and the elliptic boundary is defined by

$$\mathcal{B} = \left\{\boldsymbol{X} \text{ such that } A\left[\left(\frac{x^{(1)} - \boldsymbol{\mu}^{(1)}}{a}\right)^2 + \left(\frac{x^{(2)} - \boldsymbol{\mu}^{(2)}}{b}\right)^2 - 1\right] = 0\right\}. \quad \text{(Equation 13)}$$

Recovering the center μ

The center of the nanodomain $\mathcal{B}$ is estimated as the center of mass of the cloud of points falling inside the HDR. We use the empirical averaging formula

$$\boldsymbol{\mu} = \frac{1}{N}\sum_{i=1}^{N} \boldsymbol{X}_i, \quad \text{(Equation 14)}$$

where N is the total number of points such that $X_i \in \mathcal{B}$.

Covariance matrix Σ

We use the sample estimator of the covariance matrix Σ defined for a cloud of N two-dimensional points $\boldsymbol{X}_i = (x_i^{(1)}, x_i^{(2)})$ as

$$\sigma^{(u,v)} = \frac{1}{N-1}\sum_{i=1}^{N}\left(x_i^{(u)} - \mu^{(u)}\right)\left(x_i^{(v)} - \mu^{(v)}\right). \quad \text{(Equation 15)}$$

Confidence ellipse estimation $\varepsilon = (\mu, \boldsymbol{a}, \boldsymbol{b}, \varphi)$

We define the boundary of the well as the X% confidence ellipse of the associated Gaussian density distribution of center $\boldsymbol{\mu}$ and covariance Σ. Using the singular value decomposition method, we decompose the covariance matrix Σ as

$$\Sigma = UDV \quad \text{(Equation 16)}$$

where U, V are unitary matrices and D is diagonal. The values in D represent the variance in each dimension along the principal components. For a Gaussian density, the values of D follow a Chi-Squared distribution with $n = 2$ degrees of freedom. Therefore the semi-axes lengths a, b can be obtained at the x% confidence level from D as

$$a = \sqrt{\psi_x D^{(1,1)}}, \quad b = \sqrt{\psi_x D^{(2,2)}}, \quad \text{(Equation 17)}$$

with ψ_x is given by $P(v < \psi_x) = x$ for a Chi-Squared distribution with two degrees of freedom (for example $\psi_{99} = 9.210$, $\psi_{95} = 5.991$ and $\psi_{90} = 4.605$). Finally, the orientation φ of the ellipse is defined by the angle

$$\varphi = \operatorname{atan}\left(\frac{U^{(2,1)}}{U^{(1,1)}}\right). \quad \text{(Equation 18)}$$

CellPress
OPEN ACCESS

Maximum likelihood estimators (MLE) based on an Ornstein-Uhlenbeck model

Using the potential well representation from Equation 12 in the stochastic model presented in Equation 2 leads to a truncated Ornstein-Uhlenbeck process, centered at μ, with an attraction coefficient λ and diffusion coefficient $\sigma = \sqrt{2D}$. The probability density function $p(\boldsymbol{X}(t_{j+1})|\boldsymbol{X}(t_j))$ for $j = 1..(M-1)$ of observing two successive positions of the same trajectory $\boldsymbol{X}(t_j)$ and $\boldsymbol{X}(t_{j+1})$, separated by a time step $t_{j+1} - t_j = \Delta t$ is given by

$$\boldsymbol{X}(t_{j+1})|X(t_j) \sim \mathcal{N}(m(X(t_j)), s), \qquad \text{(Equation 19)}$$

with

$$m(X(t_j)) = X(t_j)e^{-\lambda\Delta t} + \mu\left(1 - e^{-\lambda\Delta t}\right) \qquad \text{(Equation 20)}$$

and

$$s = \frac{\sigma^2\left(1 - e^{-2\lambda\Delta t}\right)}{2\lambda}, \qquad \text{(Equation 21)}$$

which we rewrite as

$$\begin{aligned} m(X(t_j)) &= \mu\lambda\beta + X(t_j)(1 - \lambda\beta) \\ s &= \sigma^2\left(\beta - \frac{1}{2}\lambda\beta^2\right), \end{aligned} \qquad \text{(Equation 22)}$$

and

$$\beta = \frac{1 - e^{-\lambda\Delta t}}{\lambda}. \qquad \text{(Equation 23)}$$

The log-likelihood function of observing the successive pairs $(\boldsymbol{X}_i(t_j), \boldsymbol{X}_i(t_{j+1})), i = 1\ldots N$, possibly from various trajectories, is given by

$$\begin{aligned} \mathcal{L}(\mu, \lambda, \sigma|X_1, .., X_n) &= \sum_{i=1}^{N} \log(p(X_i(t_{j+1}), X_i(t_j))) \\ = \sum_{i=1}^{N}&\left[\log\left(\frac{1}{\sqrt{2\pi s}}\right) - \frac{(X_i(t_{j+1}) - m(X_i(t_j)))^2}{2s}\right] \\ = -\frac{1}{2}\sum_{i=1}^{N}&\left[\log(2\pi s) + \frac{(X_i(t_{j+1}) - m(X_i(t_j)))^2}{s}\right]. \end{aligned} \qquad \text{(Equation 24)}$$

The corresponding maximum likelihood estimators for λ and D, $\tilde{\lambda}$ and $\tilde{D}$ are obtained by solving the system of equations

$$\begin{aligned} \frac{\partial\mathcal{L}}{\partial\mu} &= 0 \\ \frac{\partial\mathcal{L}}{\partial\lambda} &= 0 \\ \frac{\partial\mathcal{L}}{\partial D} &= 0, \end{aligned} \qquad \text{(Equation 25)}$$

from which we obtain the empirical estimators for the drift coefficient (Tang and Chen, 2009)

$$\tilde{\lambda}_N = -\frac{1}{\Delta t}\log\left(\frac{\left(\sum_{i=1}^{N} X_i(t_{j+1})X_i(t_j)\right) - \left(\frac{1}{N}\sum_{i=1}^{N} X_i(t_j)\right)\left(\sum_{i=1}^{N} X_i(t_{j+1})\right)}{\left(\sum_{i=1}^{N} X_i(t_j)^2\right) - \frac{1}{N}\left(\frac{1}{N}\sum_{i=1}^{N} X_i(t_j)\right)^2}\right), \qquad \text{(Equation 26)}$$

and the diffusion coefficient:

$$\tilde{D}_N = \frac{\lambda}{N(1 - e^{-2\lambda\Delta t})}\sum_{i=1}^{N}[X_i(t_{j+1}) - m(X_i(t_j))]^2. \qquad \text{(Equation 27)}$$

Note that the parameter λ in Equation 27 can be computed from the estimator $\tilde{\lambda}_N$.

Hybrid density-drift algorithm

In this sub-section, we present two variants of an algorithm to detect the main characteristics of a potential well from some observed trajectories: the center μ, the semi-axes lengths $a \geq b$, the orientation φ, the attraction coefficient A, the diffusion coefficient D and the energy E.

Fixed spatial scale hybrid density-drift algorithm

Initiation

Search for high-density regions: the image is partitioned by a grid $G_{\Delta x}$ with square bins of size Δx. from which we compute the density map $\rho_{\Delta x}(x)$ (Equation 10). We then select the bins from $\rho_{\Delta x}(x)$ with the highest d% density as possible "seed" regions containing a potential well.

Iterations

For each seed region obtained in the initiation step, we apply an iterative procedure that is going to consider increasingly larger square neighborhoods around this region. For each iteration $k = 1..K$, we keep only the trajectories contained inside the square $\Gamma_{k,\Delta x}$ of size $[(2k+1)\times(2k+1)](\Delta x)^2$ and centered at point μ_{k-1}. The point μ_k is the center of mass of points falling in the square Γ_k (Equation 14) and μ_0 is the center of the initial high-density bin. The elliptic semi-axes a_k, b_k are computed as the x% confidence ellipse (Equation 17, `ellPerc` parameter as defined in Methods S1) from the covariance matrix C_k (Equation 15) and the angle φ_k is the orientation of the ellipse (Equation 18). These parameters define the elliptic boundary of the well at iteration k

$$\varepsilon_k(\Delta x) = (\mu_k, a_k, b_k, \varphi_k). \quad \text{(Equation 28)}$$

The attraction coefficient A_k and diffusion coefficient D_k are computed from Equations 26 and 27 respectively, for the trajectories contained inside ε_k. Specifically, we obtain A_k from

$$\lambda_k^{(1)} = \frac{2A_k}{a_k^2} \text{ and } \lambda_k^{(2)} = \frac{2A_k}{b_k^2}. \quad \text{(Equation 29)}$$

We repeat this procedure K times, with $K = \left\lceil \frac{M_s}{\Delta x} \right\rceil$ for the spatial parameter Δx and the maximum region size M_s, defined by the user.

Termination

This step consists in selecting the best iteration among K: we evaluate for each iteration $k>1$ the likelihood score $\mathcal{L}_k = \mathcal{L}(\mu_k, \lambda_k, \sigma_k | X_1, ..X_p \in \varepsilon_k - \varepsilon_{k-1})$ defined by Equation 24 but computed for sub-trajectories falling in the ring formed by the ellipses ε_{k-1} and ε_k. In practice, we filter out rings with $<$ `ringMinPts` trajectories which is a value specified by the user (see Methods S1). The best iteration k^* is selected as the first local maximum of the curve $\mathcal{L}_k$.

Multiscale hybrid algorithm

We generalize the hybrid density-drift algorithm defined above for a fixed spatial scale, by now varying the grid size Δx, in the range $\Delta x_1 < \Delta x_2 < .. < \Delta x_N$ selected by the user. The purpose of this algorithm is to select the optimal size Δx_{i^*} that maximizes the likelihood

$$k^*_{\Delta x_i^*} = \max_{i=1..N} \mathcal{L}(\mu_{k^*}, \lambda_{k^*}, \sigma_{k^*} | X_1, ..X_p \in \varepsilon_{k^*}(\Delta x_i)), \quad \text{(Equation 30)}$$

where $k^*_{\Delta x_i^*}$ is the iteration that maximizes $\mathcal{L}$ across all the spatial scales $\Delta x_i, i = 1..N$. In practice the range of grid sizes is specified by a minimal value $\Delta x_{\min}$, a step Δx_{step} and a maximum value $\Delta x_{\max}$ as presented in Methods S1.

Density-based algorithm

The Density-Based Algorithm uses the density of points estimated around the local density maximum of a high-density region and was first presented in (Parutto et al., 2019). The algorithm uses the level set of a Gaussian distribution of points inside a potential well. We define the level set Γ_α with respect to a local maximum M^* as the ensemble of all trajectory points falling in bins with a density greater than αM^*:

$$\Gamma_\alpha = \{\boldsymbol{X}_i \text{ such that } \rho_e(\boldsymbol{x}) > \alpha M^*\}, \quad \text{(Equation 31)}$$

where ρ_e is the empirical point density, estimated over the bins of a square grid (Equation 10) and $\alpha \in [0,1]$ is a density threshold. For the points $\boldsymbol{X}_i = (x_i^{(1)}, x_i^{(2)})$ located in Γ_α, the center $\boldsymbol{\mu}$ of the distribution is approximated by the empirical estimators based on Equation 14 but restricted to the points in Γ_α:

$$\hat{\mu}_\alpha^{(u)} = \frac{1}{N_p} \sum_{\{k=1, \mathbf{X}_k \in \Gamma_\alpha\}}^{N_p} x_k^{(u)}, \quad \text{(Equation 32)}$$

with N_p the number of points in the ensemble Γ_α and $u = 1..2$. Similarly, we extend the estimator for the covariance matrix Σ (Equation 15) to

$$\widehat{\sigma}_{\alpha}^{(u,v)} = \frac{1}{N_p - 1} \sum_{\{k=1, \mathbf{X}_k \in \Gamma_\alpha\}}^{N_p} \left(x_k^{(u)} - \widehat{\mu}_\alpha^{(u)}\right)\left(x_k^{(v)} - \widehat{\mu}_\alpha^{(v)}\right). \quad \text{(Equation 33)}$$

We now define the density-based algorithm:

Initiation

Search for high-density regions: the image is partitioned by a grid $G_{\Delta x}$ with square bins of size Δx from which we compute the density map $\rho_{\Delta x}(x)$ (Equation 10). We then select the bins from $\rho_{\Delta x}(x)$ with the highest d% density as possible regions containing a potential well.

Iterations

For each selected high-density bin, we initialize the well center $\tilde{\boldsymbol{\mu}}_0$ at the center of the bin. We then construct a refined grid centered at $\tilde{\boldsymbol{\mu}}_0$ and with bin size $\Delta x' < \Delta x$ (parameter `locGridDx` as defined in Methods S1). In this grid, we compute the centers $\boldsymbol{\mu}_{0,\alpha_k}$, for different values of α: $\alpha_1 < \ldots < \alpha_N$, selected by the user. The refined center $\boldsymbol{\mu}_0$ is obtained as the center of mass of the centers $\boldsymbol{\mu}_{0,\alpha_k}$ for $k = 1..N$.

We then apply an iterative procedure that considers increasingly larger concentric annulus of center $\boldsymbol{\mu}_0$, width Δr and radius r_k for $k = 1..K$. Where the number of iterations K is determined based on a minimal $r_1 = r_{\min}$ and maximal $r_K = r_{\max}$ distances defined by the user (see Methods S1). For each iteration k, we compute the confidence ellipse $\varepsilon_k = (\boldsymbol{\mu}_0, a_k, b_k, \varphi_k)$ (see sub-section Method details) obtained from the covariance matrix Σ_k (Equation 33) computed only for the points that fall in the annulus of radius r_k. We then search for the iteration r^* that maximizes the ratio $C_v(r_k) = \sqrt{a_k/b_k}$ (in practice, we limit the maximal distance for finding C_v to $r_k <$ `ratMaxDist`, which value is specified by the user (see Methods S1)) and use it to define the refined distance to the center

$$r_e(\boldsymbol{X}) = \sqrt{\left(x^{(1)} - \mu_0^{(1)}\right)^2 + \kappa\left(x^{(2)} - \mu_0^{(2)}\right)^2}, \quad \text{(Equation 34)}$$

where $\kappa = C_v(r^*)$, that transforms an ellipse into a circle with the same center. Finally, we compute the density of points $N_e(r_k)$ falling in the annulus of radius r_k based on the refined distance measure r_e.

Termination

We select the first iteration k^* such that $N_e(r_k^*) > N_e(r_{k^*-1})$: it is the first iteration where the derivative of the density with respect to the distance to the center, stops decreasing. This criteria is more stable on empirical data than searching for the minimum of the density (see Figures 3 and 4 panel B3 of (Parutto et al., 2019)). The elliptic boundary of the well ε^* is centered at $\boldsymbol{\mu}_0$, has semi-axis lengths a^*, b^* given by $a^* = r_{k^*}$ and $b^* = \kappa r_{k^*}$ and orientation φ_{k^*}. We then use the ML estimator (Equation 27) to estimate the constant diffusion coefficient D inside ε^*. Finally, to compute A^* we use the diagonal form of the covariance matrix estimated (Equation 33) for all the points falling in ε^*:

$$\Sigma = \frac{D}{A}\begin{bmatrix} a^2 & 0 \\ 0 & b^2 \end{bmatrix}. \quad \text{(Equation 35)}$$

and estimate

$$A^* = \frac{D}{2}\left(\frac{(a^*)^2}{\sigma_{11}} + \frac{(b^*)^2}{\sigma_{22}}\right). \quad \text{(Equation 36)}$$

Drift-based algorithm

The drift based algorithm uses an error function in the space of the vector field to estimate the characteristics of a well and was introduced in (Heck et al., 2019). Its principle is as follows:

Initiation

Search for high-density regions: the image is partitioned by a grid $G_{\Delta x}$ with square bins of size Δx from which we compute the density map $\rho_{\Delta x}(x)$ (Equation 10). We then select the bins from $\rho_{\Delta x}(x)$ with the highest d% density as possible regions containing a potential well.

Iterations

For each region selected in the initiation, we apply the following iterative procedure for $k = 1..K$:

(a) We select only the sub-trajectories falling into a square $\Gamma_k(\boldsymbol{\mu}_k, \Delta x)$ centered at $\boldsymbol{\mu}_{k-1}$ and of size $(2k+1)\Delta x \times (2k+1)\Delta x$. We take $\boldsymbol{\mu}_0$ to be the center of the high-density bin.

(b) We estimate the elliptic well boundary $\varepsilon_k = [\boldsymbol{\mu}_k, a_k, b_k, \varphi_k]$ as the x% confidence ellipse (parameter `ellPerc` as discussed in Methods S1) from the cloud of points in $\Gamma_k(c_k, \Delta x)$. Where x is a parameter selected by the user (usually 90, 95 or 99).

(c) We then compute a new grid $G_{\Delta x}(\boldsymbol{\mu}_k)$ centered at $\boldsymbol{\mu}_k$, that we use to estimate the local drift map (Equation 6) $a_k(\boldsymbol{X}) = [a_k^{(1)}(\boldsymbol{X}), a_k^{(2)}(\boldsymbol{X})]$ and estimate the attraction coefficient A_k of the well using the least-square regression formula

$$A_k = \frac{1}{2}\frac{\sum_{i=1}^{M}\frac{a_k^{(1)}(\boldsymbol{X}_i)x_i^{(1)}}{a^2}+\frac{a_k^{(2)}(\boldsymbol{X}_i)x_i^{(2)}}{b^2}}{\sum_{i=1}^{M}\frac{\left(x_i^{(1)}\right)^2}{a^4}+\frac{\left(x_i^{(2)}\right)^2}{b^4}}, \quad \text{(Equation 37)}$$

where $\boldsymbol{X}_i = [x_i^{(1)}, x_i^{(2)}]$ $(i = 1\ldots M)$ are the centers of the M bins from $G_{\Delta x}(\boldsymbol{\mu}_k)$ that are contained inside the ellipse ε_k.

(d) Finally, we estimate the quality of the well (parabolic index) based on the residual least square error:

$$S_k(a_k, A_k) = 1 - \frac{\left(\sum_{i=1}^{M}\frac{a_k^{(1)}(\boldsymbol{X}_i)x_i^{(1)}}{a^2}+\frac{a_k^{(2)}(\boldsymbol{X}_i)x_i^{(2)}}{b^2}\right)^2}{\left(\sum_{i=1}^{M}\frac{\left(x_i^{(1)}\right)^2}{a^4}+\frac{\left(x_i^{(2)}\right)^2}{b^4}\right)\left(\sum_{i=1}^{M}\|a_k(\boldsymbol{X}_i)\|^2\right)}. \quad \text{(Equation 38)}$$

The index $S_k \in [0,1]$ is defined such that $S_k \to 0$ for a drift field generated by a parabolic potential well and $S_k \to 1$ for a random drift vector field as observed for diffusive motion.

The number of iterations is given by $N = \lfloor w_{\max} / \Delta x \rfloor$ where $w_{\max}$ is the maximum size of the region to consider and is given by the user.

Termination

We select the iteration k^* that minimizes the parabolic index S: $k^* = \mathrm{argmin}_{k=1\ldots K} S_k(a_k, A_k)$. We estimate the diffusion coefficient inside the well using the local estimator (Equation 4) for all the displacements inside the ellipse ε_{k^*}.

Sliding window analysis to study the stability of the wells over time

To determine the stability of the potential wells, we use a non-overlapping sliding window of 20 s (Heck et al., 2019), to recover the ellipse characteristics, as shown on different examples in Figure S6A and S6C. When a well disappears in a given time window, but reappears latter, we kept the well for the entire period.

Reconstructing the graph of the network explored by SPTs

We describe here a new variant of the algorithm to reconstruct a graph from SPTs exploring a network. This algorithm is based on the graph reconstruction algorithm introduced in (Holcman et al., 2018) and exploits the heterogeneous distribution of points caused by trajectories spending more time inside nodes than in tubules.

Recursive velocity based graph reconstruction algorithm of a network explored by SPTs

The recursive graph reconstruction algorithm first generates an ensemble of points from slow trajectory displacements based on a maximum displacement length threshold v_{th} and then recursively uses the `dbscan` algorithm (Ester et al., 1996) to cluster these points based on their local density. The algorithm requires specification of two ensembles of parameters:

1. An ensemble of distances $R = \{R_u, u = 1..U\}$ (in μm) defining the neighborhood radius around points.
2. An ensemble of counts $N = \{N_v, v = 1..V\}$ defining the numbers of points required in the neighborhood to form a cluster (Ester et al., 1996).

A pair of these two parameters (R_u, N_v) for any u, v defines a local density $\frac{N_v}{R_u}$ (points/ μm^2) inside each cluster. The values of R and N depend on the local number of recorded trajectories and can vary inside the image. For each dataset, these values can be determined such that the computed clusters overlap with the structure of the organelle formed by the trajectories.

We now present the steps of the algorithm:

1. We form the ensemble of points belonging to low-velocity trajectory fragments $S = \left\{X_i(t_j), \left|\frac{\|\Delta X_i(t_j)\|}{\Delta t} \leq v_{th}\right.\right\}$.
2. We apply the `dbscan` procedure with parameters (R_U, N_1) to obtain a first ensemble of K clusters $c_1, \ldots, c_K$ from the points in S.
3. We then perform a "recursive" step where we refine the initial clusters possessing more than `maxClustNpts` points. For any such cluster c_k:
   (a) We iteratively re-apply the `dbscan` algorithm with the more stringent parameter pair (R_u, N_v) for $u = (U-1)..1$ and $v = 2..V$ and replace the initial cluster with the resulting sub-cluster(s). We continue iterating over the generated sub-clusters until they all possess less than `maxClustNpts` points.
4. We then approximate the boundary of each cluster either by its minimum volume ellipsoid (Todd, 2016) or its convex hull polygon and assign each point discarded in step 1 to the cluster in which they fall if possible.
5. Finally, we merge any overlapping pair of clusters by computing the boundary of the combined ensemble of points (either elliptic or the convex hull) and we iterate this procedure until no more clusters overlap.

This first step allows to find the K nodes of the network. In the second step, we define tubules by constructing a connectivity matrix C of size $K \times K$ where $c_{i,j}$ $(1 \le i,j \le K)$ is the number of first or second order trajectory segments connecting nodes i and j. Specifically, we increment $c_{i,j}$ for each data point $X_k(t_l)$ $(1 \le k \le N_t,\ 0 \le l < M_k - 1)$ in the following cases:

1. $X_k(t_l)$ is located in node i and $X_k(t_{l+1})$ in node j
2. $X_k(t_l)$ is located in node i, $X_k(t_{l+1})$ does not belong to any node and $X_k(t_{l+2})$ is located in node j (in this case $0 \le l < M_k - 2$).

Lysosome analysis

Trajectories analysis for lysosome SPTs

To study the dynamics of lysosomes, we plotted the distribution of instantaneous velocities, computed from each trajectory displacement $\boldsymbol{X}(t+\Delta t) - \boldsymbol{X}(t)$ by

$$v = \frac{\boldsymbol{X}(t+\Delta t) - \boldsymbol{X}(t)}{\Delta t}, \quad \text{(Equation 39)}$$

where $\Delta t = 1.5$ s. We approximate the distribution of instantaneous velocities using a two exponential model obtained by fitting $f(v) = Ae^{-\frac{v}{v_0}} + Be^{-\frac{v}{v_1}}$ to the distribution using MATLAB's `fit` toolbox. The density and diffusion maps were computed using the estimators from Equation 4 described above.

Local high-density region analysis: Ellipse approximation of the boundary

High-density regions are extracted from trajectories as follows: we construct a density map (Equation 10) based on a square grid with bin size $\Delta x = 480$ nm. From this density map, we select only the 5% highest density bins and in case multiple such bins appear within a distance of two squares of each other, only the one with the highest value was kept. For each selected bin of center c, we computed a refined density map of size 5×5 squares, centered at c and with bin size $\Delta x' = 200$ nm. From this local map, we collected trajectory points falling into the bins that have a density > 80% of the maximal bin value and use them to estimate the elliptic boundary of the region from the 95% confidence ellipse (see sub-section Method details). Finally, when a pair of ellipses overlap, we replaced them by the ellipse computed over their combined ensemble of points and iterated this procedure until no more overlaps could be found.

Transient confinement detection

To detect transient confinement periods along individual trajectories, we used the following procedure: for each point $\boldsymbol{X}(t_j)$ of a trajectory, we considered the ensemble of its successors $e_{t_j,n} = \{\boldsymbol{X}(t_j), \ldots, \boldsymbol{X}(t_{j+n})\}$, where initially $n = N_{nh}$ is set by the user. We then computed the center of mass $\mu_{t_j,n}$ and checked that all the points $\boldsymbol{X}(t_k) \in e_{t_j,n}$ have a distance to the center of mass $\|\boldsymbol{X}(t_j) - \mu_{t_k,n}\| < R_{nh}$, for a chosen distance threshold R_{nh} ($\|.\|$ is the Euclidean norm). We then iterate the procedure, considering increasingly larger ensembles of successors $n = \{N_{nh}, N_{nh}+1, \ldots, N_{nh}+K\}$ until either reaching the end of the recorded trajectory or when the next point $\boldsymbol{X}(t_{j+n+1})$ do not fall into the neighborhood of the center of mass $\mu_{t_j,n}$. The confinement duration is then computed by considering the difference $t_{j+n} - t_j$ in time between the two endpoints of the ensemble. Finally, the spring constant λ and diffusion coefficient D of the confinement is obtained by applying the Ornstein-Ulenbeck maximum likelihood estimators (Tang and Chen, 2009; Parutto et al., 2019), where the OU-process is given by

$$dX = -\lambda(X - \mu)dt + \sqrt{2D}dW. \quad \text{(Equation 40)}$$

ImageJ plugin

The present method and algorithms are implemented into an ImageJ plugin called "SPTAnalysis". The plugin allows to reconstruct the various maps (trajectories, density, drift, diffusion), detect potential wells and reconstruct the graph associated to trajectories. It allows to extract various statistics such as the distribution of diffusion coefficients or the energy and the size of potential wells. The algorithms performances are summarized in Figure S9 and Method S1.

QUANTIFICATION AND STATISTICAL ANALYSIS

No statistical tests have been used in this paper. Data quantification is reported as average ± standard deviation, where the meanings of n are indicated in the corresponding legends.

Cell Reports Methods, Volume *2*

Supplemental information

High-throughput super-resolution single-particle trajectory analysis reconstructs organelle dynamics and membrane reorganization

Pierre Parutto, Jennifer Heck, Meng Lu, Clemens Kaminski, Edward Avezov, Martin Heine, and David Holcman

Supplementary Figures and Tables

P. Parutto[1], J. Heck[2], M. Lu, C. Kaminski*
E. Avezov*, M. Heine[2*] and D. Holcman[13] *

July 23, 2022

[1] Group of Data Modeling and Computational Biology, IBENS, Ecole Normale Supérieure,75005 Paris, France. [2] Research Group Functional Neurobiology at the Institute of Developmental Biology and Neurobiology, Johannes Gutenberg University Mainz, Mainz, Germany and [3] DAMPT, University Of Cambridge, DAMPT and Churchill College CB30DS, United Kingdom. equally.

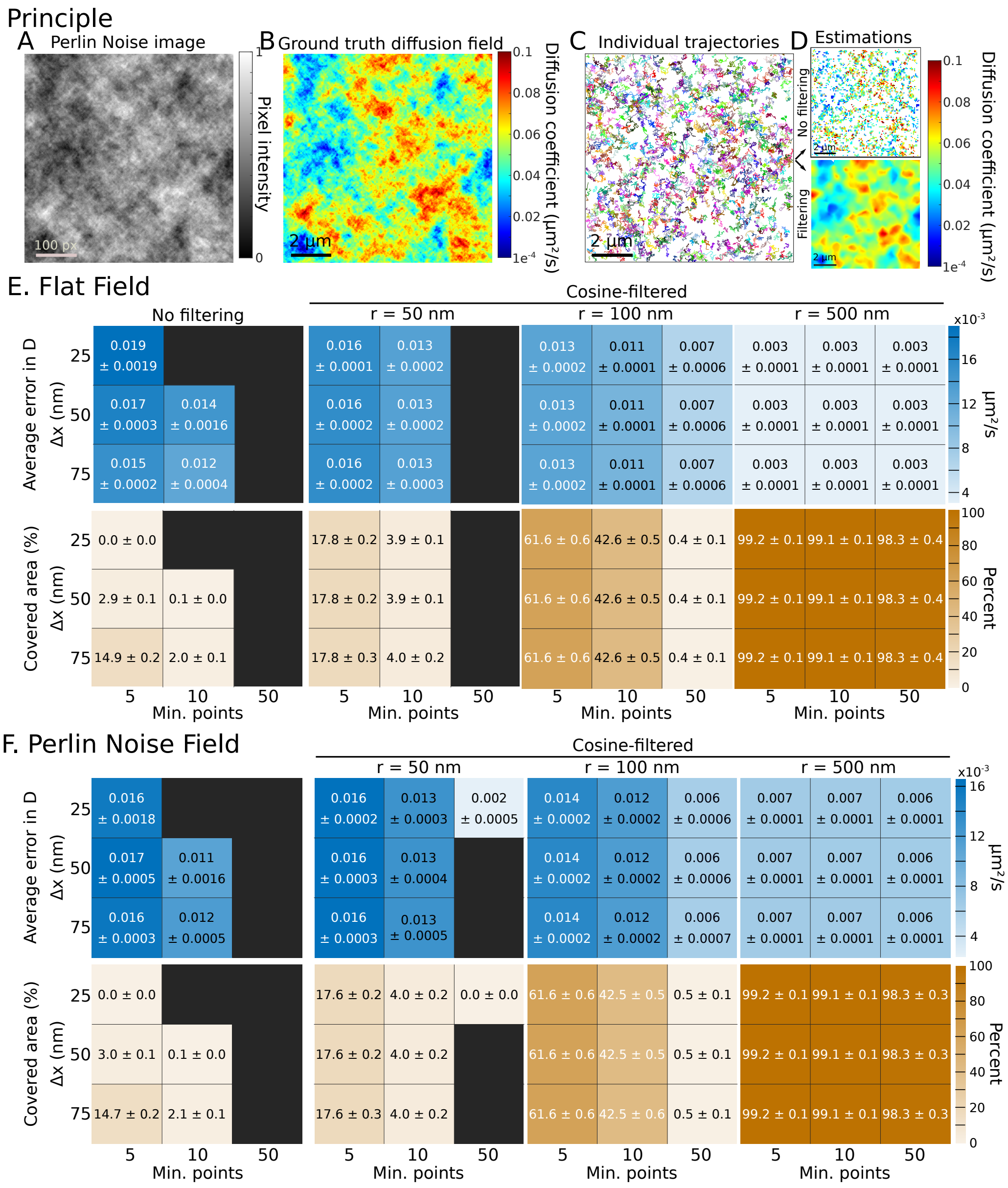

Figure S1: **Evaluation of diffusion field estimation**, related to STAR Methods. **A.** Perlin noise image used to generate a non-uniform diffusion field. **B.** The non-uniform diffusion field generated from A by setting a pixel size of 64.5 nm, $D_{min} = 1e^{-4}\mu m^2/s$ and $D_{max} = 0.1\mu m^2/s$. **C.** Individual Brownian trajectories simulated from the diffusion field presented in B. **D.** Diffusion maps estimated from the trajectories presented in C without filtering (top, parameters $\Delta x = 100$ nm, $minPts = 5$) and with cosine-filtering (bottom, parameters $\Delta x = 50$ nm, $minPts = 10$, $r_{filt} = 500$ nm). **E.** Heat maps evaluating the quality of the reconstructed diffusion field for a uniform field with $D = 0.05\mu m^2/s$ obtained over 100simulations. The top line (blue shade) reports the average error (see eq.1 of Data 1) between the reconstructed ground truth field while the bottom line (brown shade) shows the average percentage of the field area recovered. **F.** Same as E but for the non-uniform field presented in B. Black squares correspond to conditions where not enough data could be recovered.

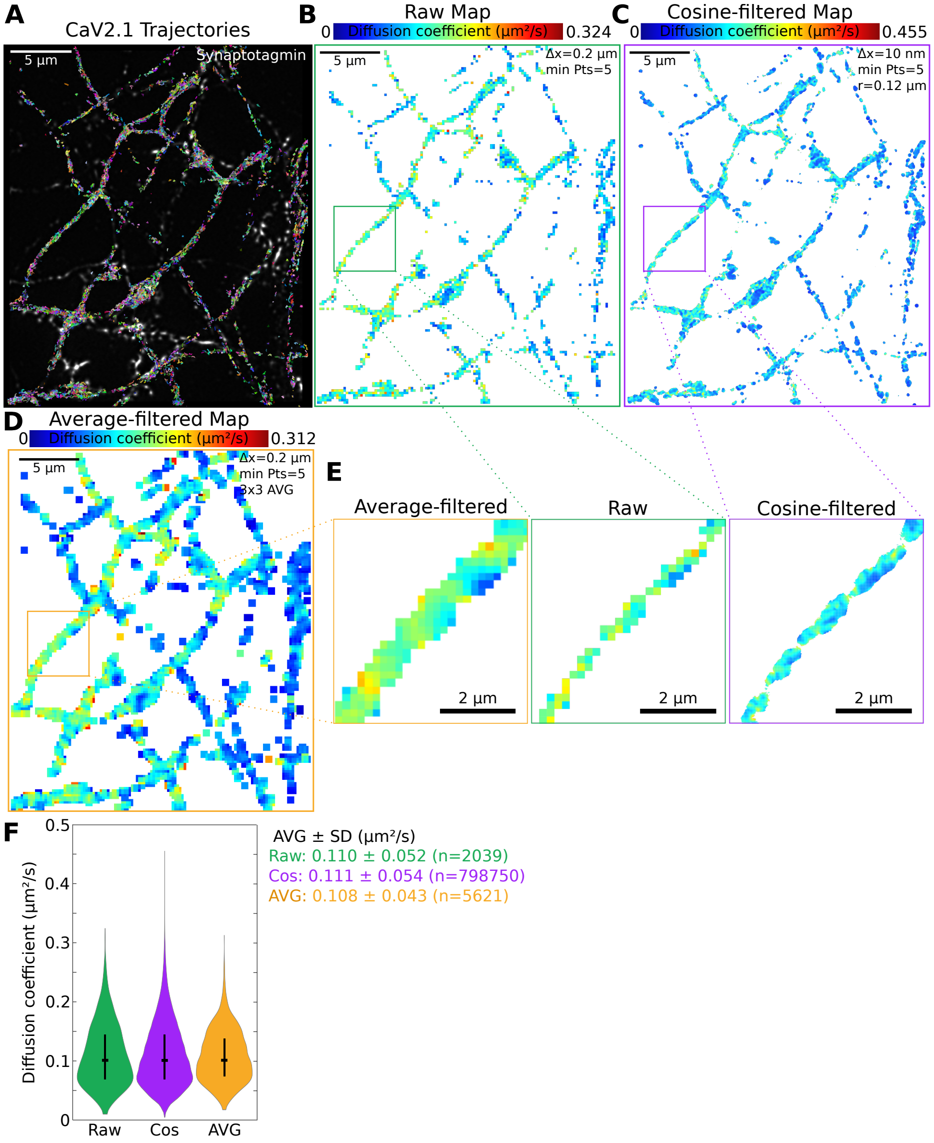

Figure S2: **Comparing the diffusion maps for CaV2.1 SPTs from three estimators**, related to STAR Methods. **A.** Individual CaV2.1+47 trajectories overlaid on top of the synaptotagmin pre-synaptic terminal marker's signal. **B.** Diffusion map computed from the classical estimator (Eq. 7 main text). **C.** Diffusion map computed with the cosine-estimator (Eq. 9 Main text). The cosine estimator is computed over a sliding window with a moving disk of radius $r_{filt} = 0.12$ μm and a grid size $\Delta x = 0.01$ μm. **D**. Map computed using a local average filter (using a 3×3 window around each bin). **E.** Comparison of the tree approaches from a representative regions. **F.** Violin plots of the diffusion coefficients computed in each bin for the three methods (n represents the number of bins).

Ground truth data sets and hybrid algorithm

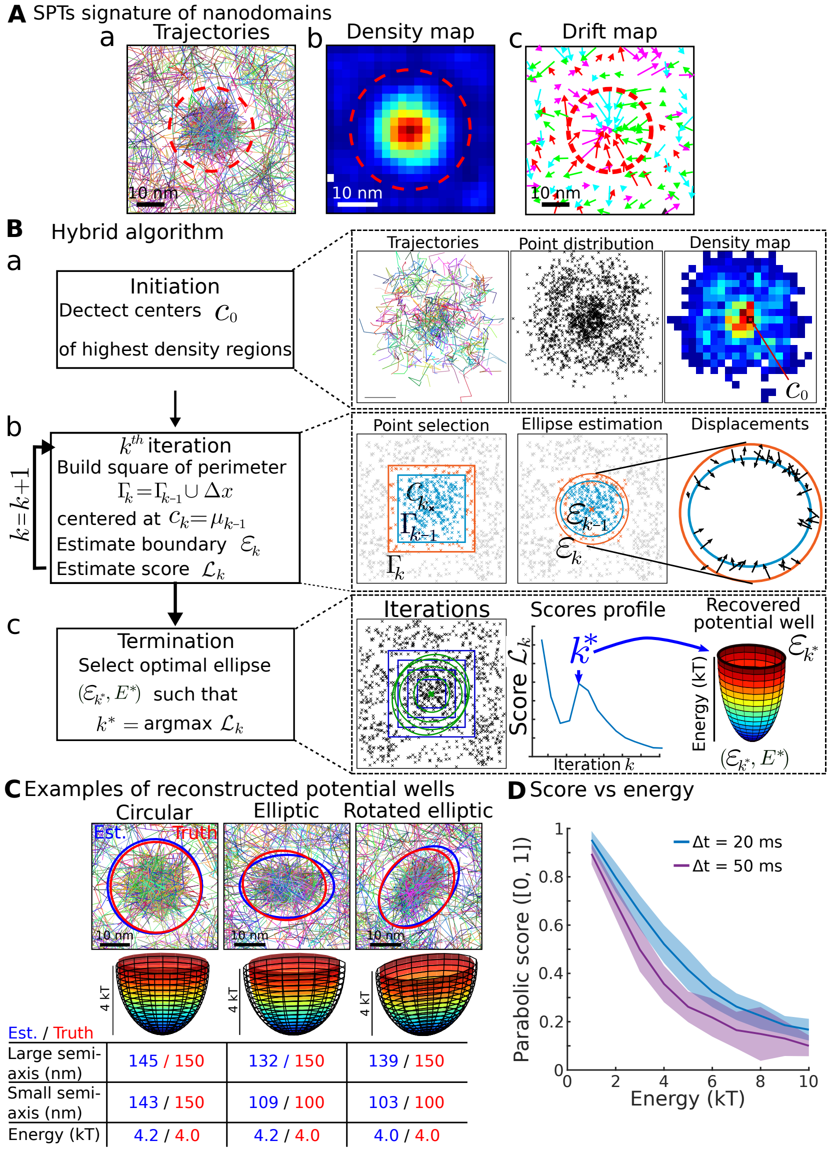

Figure S3: **Reconstruction algorithm of nanodomains from super-resolution SPTs**, related to Figures 2 and 3. **A.** a- Simulated trajectories attracted in a well of known boundary. b- density map c- drift field. **B.** Algorithm to reconstruct the center, boundary and energy of the nanodomain characterized as a potential well. Initiation of the center c_0 with an ellipse γ_0 and the center of mass μ_0. The error between the vector field and the reconstructed field is measured by the score S_0. The spatial resolution is fixed by Δx. k-iteration step: the domain is enlarged to Γ_k, the center is revaluated at point c_k and the error S_k is recomputed. In the termination step, the optimal value k^* is selected that minimizes the error S_{k^*}, leading to the optimal center and ellipse γ_{k^*}, for which the energy is computed as the ratio A/D of the field to the diffusion coefficient. **C**. Three examples of potential wells reconstructed from trajectories. **D** as a function of the energy of the well and depending on the acquisition time. The simulation, well detection algorithm and its parameters are the same as in Table S3.

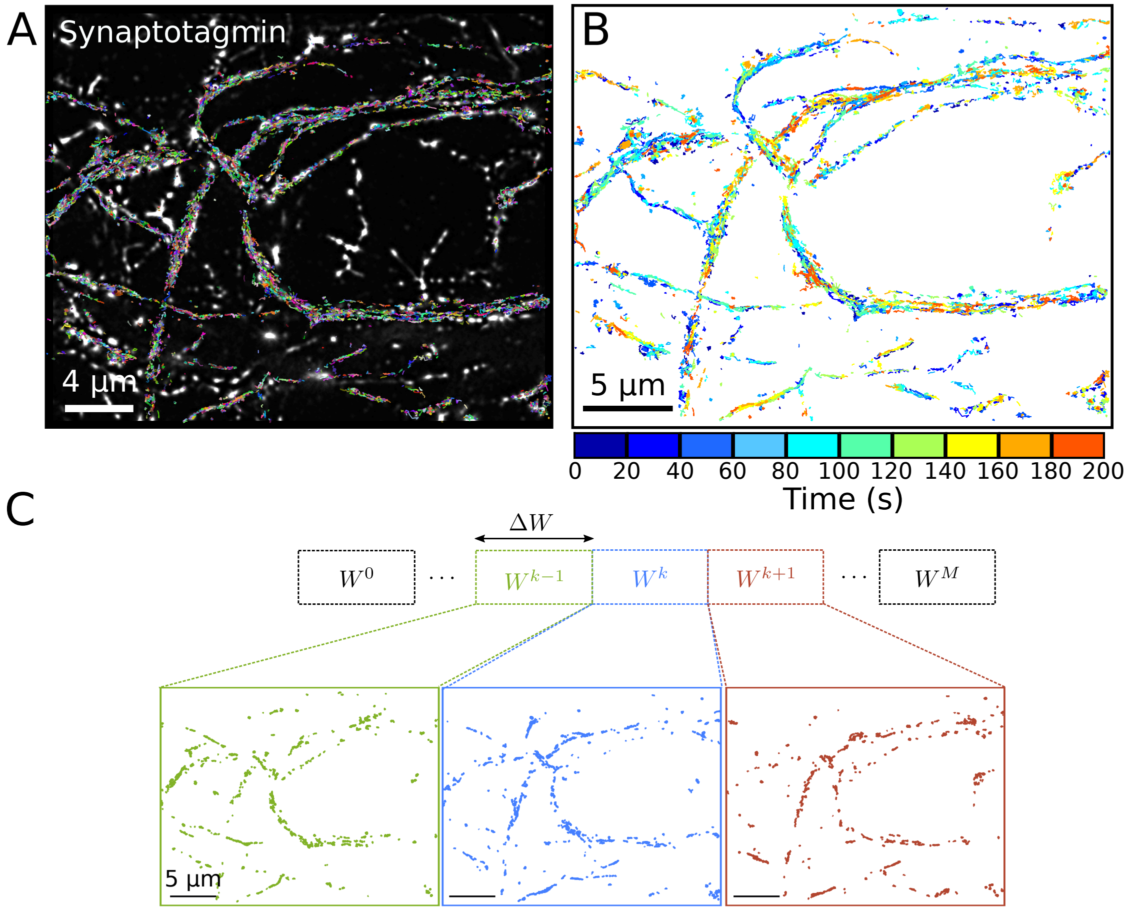

Figure S4: **Time Lapse analysis based on a sliding window**, related to Figures 2 and 3. **A.** Example of CaV2.1Δ47 SPTs plotted over the entire acquisition time and overlaid on top of the synaptotagmin pre-synaptic terminal marker's signal. **B.** Time-splitting of the dataset into 20 s time windows with no overlap. Trajectories are colored by the time window in they appear. **C.** Time-splitting principle: the entire duration spanned by the dataset is divided into windows of duration $\Delta W = 20$ s. In the case of overlapping windows, a trajectory belonging to two consecutive time windows will serve for the statistical estimations for each period.

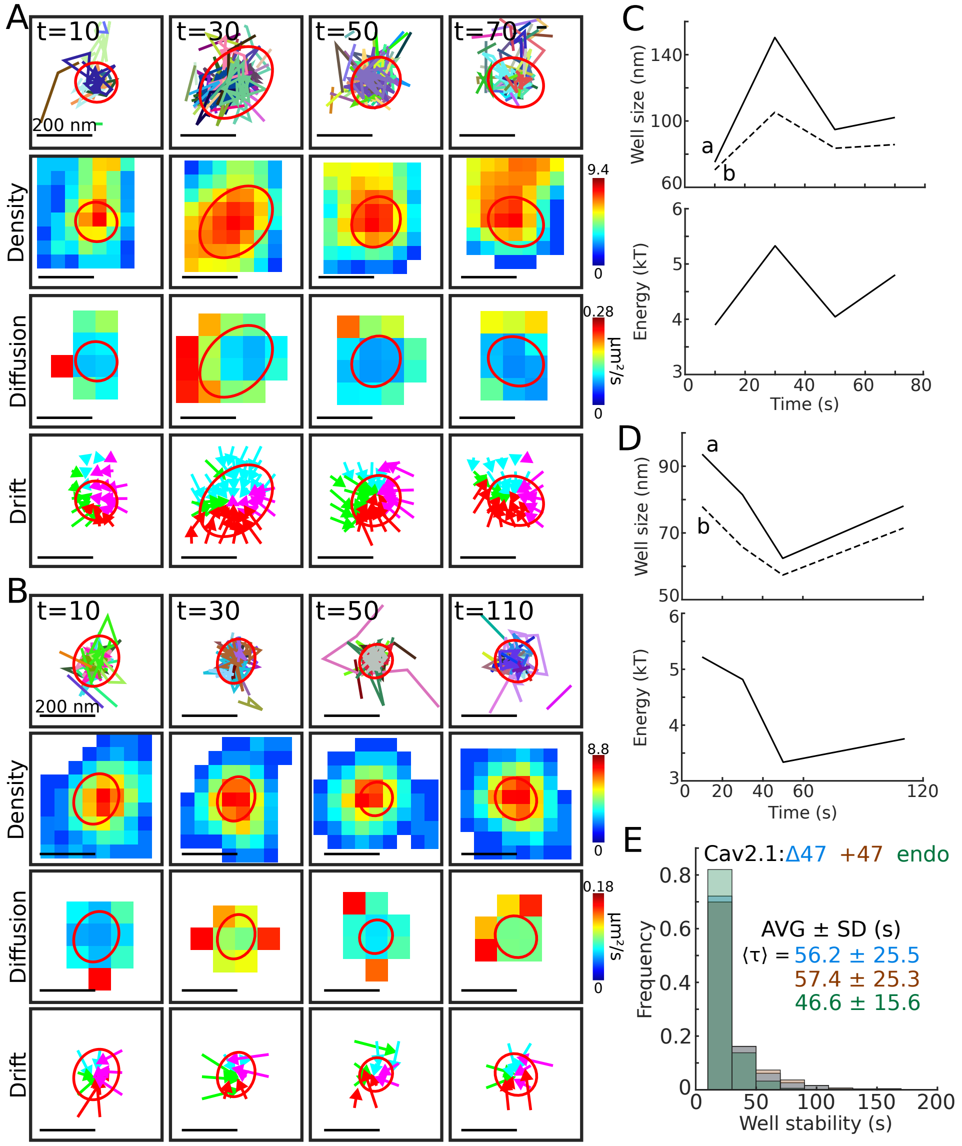

Figure S5: **Two examples of endogenous CaV2.1 nanodomains automatically identified during a time lapse analysis**, related to STAR Methods. **A.** A high density region identified as potential well in successive 20 s time windows. From top to bottom: individual trajectories, associated density, diffusion and drift maps. **B.** Another example of the temporal evolution of a potential well, similar to A. **C-D.** Temporal evolution of size and energy characteristics associated to the wells presented in A and B respectively. **E.** Population characteristics for three experimental conditions: CaV2.1Δ47, CaV2.1+47 or CaV2.1 showing the mean life time of potential wells extracted by an exponential fit (n values are given in Table S5).

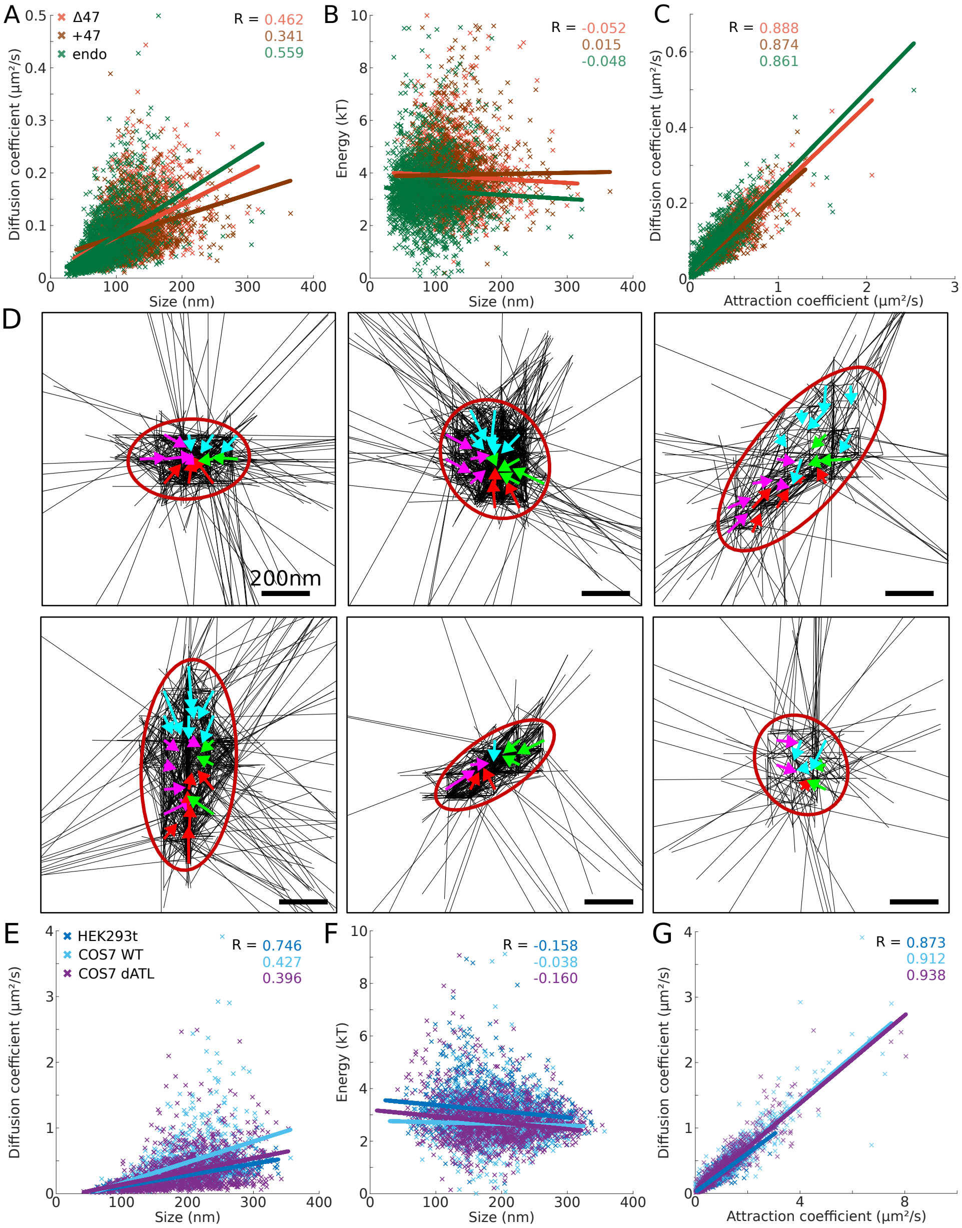

Figure S6: **Potential wells' characteristics**, related to Figures 2 and 3. **A.** Diffusion vs size (high correlation) **B.** energy vs size (no correlation). **C.** Diffusion vs attraction coefficient (high correlation). **D.** Six examples of converging arrows in potential wells associated to ER nodes. **E.** Distribution of the energy of the wells vs the size and the correlation coefficients. **F.** Energy vs size (no correlation). **G.** Diffusion coefficient vs attraction coefficient showing strong correlations between the two parameters. For A-C: $n = 1587, 1713, 1878$ for the Δ47, +47 and endogenous cases respectively; For E-G: $n = 1057, 859, 1250$ for the HEK293t, COS7WT and COS7dATL cases respectively.

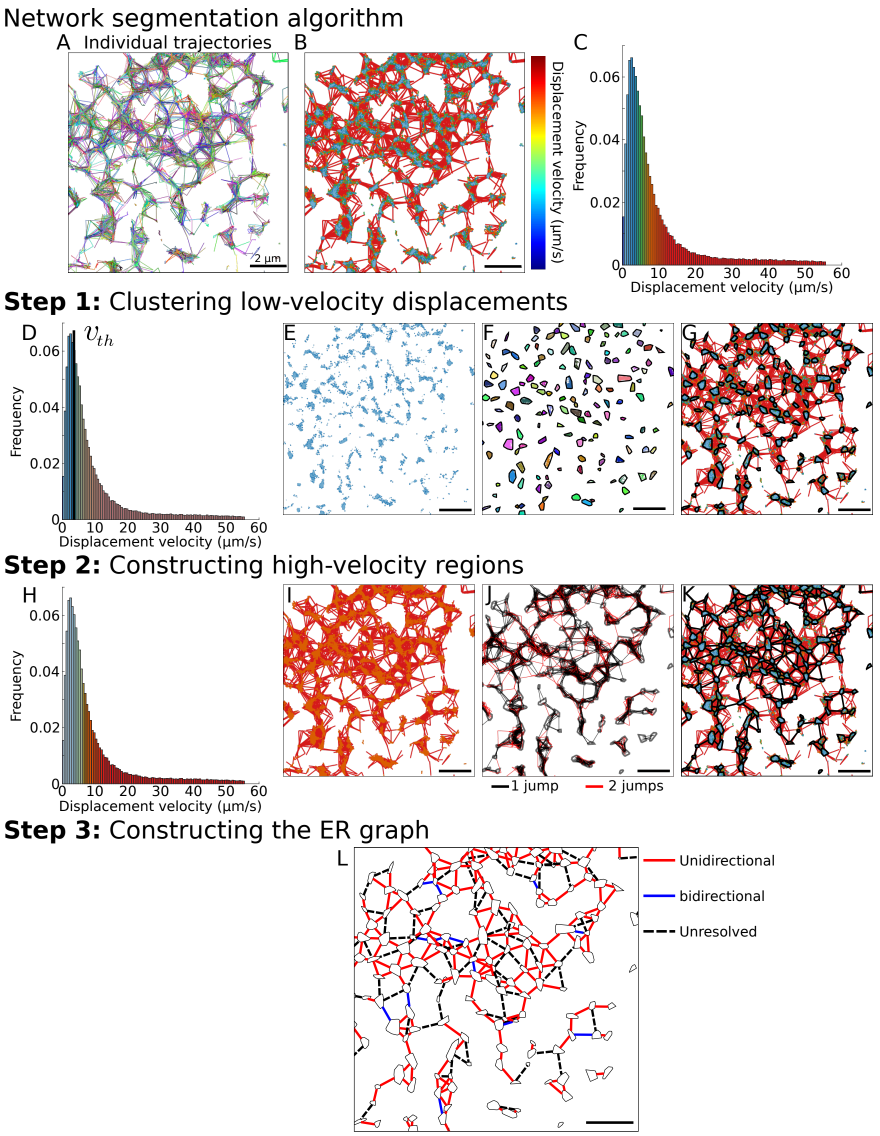

Figure S7: **Automatic network reconstruction from super-resolution SPTs**, related to Figures 4 and 7. **A.** Individually color-coded ER-luminal trajectories. **B.** the same trajectories as in A but where each displacement is color-coded by its instantaneous velocity. **C.** Distribution of instantaneous velocities color-coded according to their amplitude as in B. **D-G.** Extraction of the network nodes from low-velocity regions: a threshold v_{th} is selected on the distribution of instantaneous velocities (D) and the corresponding displacements are shown (E), these points are clustered using the dbscan algorithm (or a recursive version of it, see Method) and the convex hull of each cluster is computed to obtain its boundary (F). Finally, the corresponding boundaries are overlaid on top of the original trajectories (G). **H-K.** Extraction of the graph links from high-velocity jumps: we now select only the displacements with velocities above the threshold used in step 1 (H) shown in (I). We then filter them to keep only those that either: i. have their endpoints that fall into two separate ellipses (single-jump) extracted from step 1 or ii. successive displacements where the first point falls in an ellipse, the middle point is outside and the third point fall in a different ellipse (double-jump) (J) from which we obtained the reconstructed graph (K). **L.** A directionality analysis applied to the graph recovered from step 2 allowing to recover an oriented graph of the ER network.

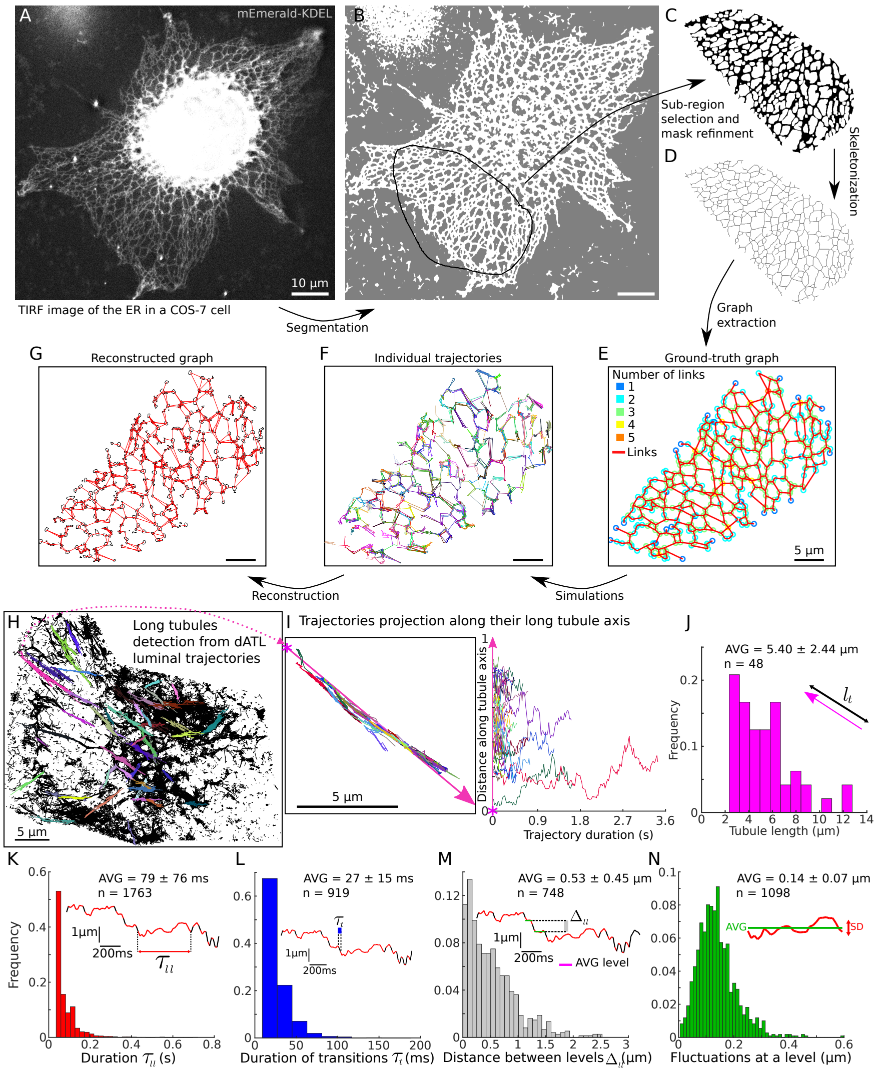

Figure S8: **Simulation of trajectories in an empirical ER network and dATL long tubule analysis**, related to STAR Methods. **A.** TIRF microscopy image of the ER network of a COS-7 cell transfected with an mEmerald-KDEL marker. **B.** Segmented image obtained with Ilastik where the ER network appears in white. **C.** Magnification on a well segmented sub-region of the ER after applying dilation filters. **D.** Skeletonization of the region from C. **E.** Graph extracted from the skeleton from D. where intersection are replaced by disks of radius $r = 400$ nm. The nodes are color-coded by the number of links they have. **F.** Individual trajectories generated from the geometry presented in E. **G.** Reconstructed graph obtain by applying the DBSCANRec algorithm to the trajectories presented in F. **H** SPTs (black) recorded in the ER of a COS-7 dATL cell with detected long-tubules (colored). **I** Magnification of a single detected long tubule (left) and projection of its trajectories along the tubule axis (right). **J** Length distribution for the long tubules detected in A (n: number of detected long tubules). **K-N** Decomposition of the projected motion of trajectories in long tubules showing stalling and transition between different levels. D: Duration distribution at a given level (n: number of detected levels), E: Distribution of transitions between two successive levels (n: number of transition events). F: Distribution of distances between two successive levels (n: number of distances between successive levels). G: Fluctuation amplitude of the positions inside a level (n: number of levels with enough data).

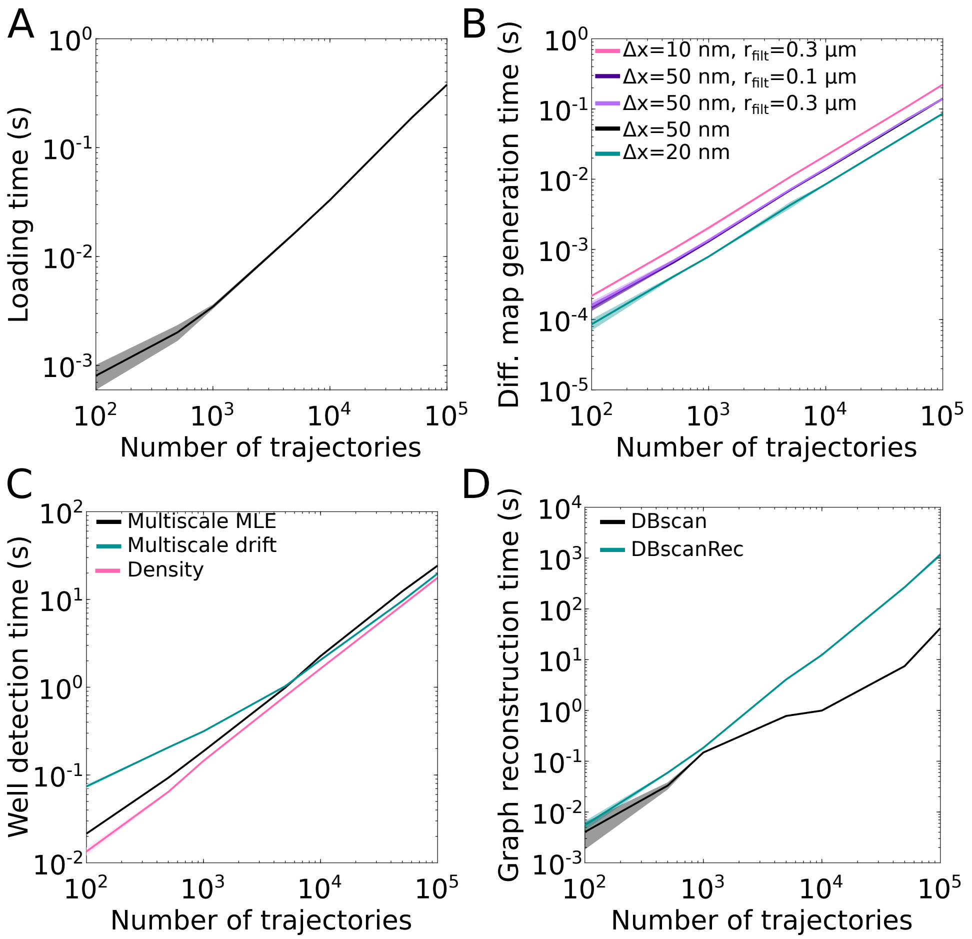

Figure S9: **Time benchmarks of the main plugin procedures**, related to STAR Methods. **A.** Loading time as a function of the number of trajectories. **B.** Diffusion maps reconstruction as a function of the number of trajectories and different set of parameters (with $minPts = 10$ in every case). **C.** Well detection time as a function of the number of trajectories for the three different methods implemented in the plugin. **D.** Graph reconstruction time as a function of the number of trajectories for the two different reconstruction algorithms implemented in the plugin. All values are given as average (solid line) ± std (shade) over 100 different sets of trajectories.

	Algo	Δt=20 ms		Δt=50 ms	
		E=4 kT	E=6 kT	E=4 kT	E=6 kT
$\|\|\mu - \mu_{true}\|\|$ (nm)	MLE	8 ± 6	5 ± 3	5 ± 4	4 ± 2
	Drift	26 ± 10	12 ± 5	19 ± 9	7 ± 3
	Dens	32 ± 5	24 ± 4	20 ± 4	14 ± 3
a (nm)	MLE	159 ± 15	146 ± 17	162 ± 17	140 ± 22
	Drift	136 ± 12	135 ± 10	142 ± 12	142 ± 11
	Dens	145 ± 24	145 ± 11	153 ± 14	148 ± 8
b (nm)	MLE	152 ± 14	140 ± 16	156 ± 17	134 ± 21
	Drift	126 ± 12	126 ± 10	132 ± 12	134 ± 11
	Dens	139 ± 23	139 ± 13	149 ± 14	144 ± 9
A (μm^2/s)	MLE	0.24 ± 0.03	0.29 ± 0.05	0.25 ± 0.04	0.27 ± 0.08
	Drift	0.12 ± 0.03	0.17 ± 0.03	0.09 ± 0.02	0.13 ± 0.02
	Dens	0.16 ± 0.02	0.22 ± 0.02	0.19 ± 0.02	0.26 ± 0.02
D (μm^2/s)	MLE	0.050 ± 0.002	0.051 ± 0.002	0.050 ± 0.003	0.050 ± 0.004
	Drift	0.043 ± 0.001	0.041 ± 0.001	0.034 ± 0.001	0.029 ± 0.001
	Dens	0.049 ± 0.003	0.051 ± 0.002	0.050 ± 0.003	0.051 ± 0.003
E (kT)	MLE	4.78 ± 0.52	5.72 ± 1.03	4.88 ± 0.67	5.28 ± 1.38
	Drift	2.71 ± 0.57	4.07 ± 0.63	2.75 ± 0.46	4.41 ± 0.62
	Dens	3.35 ± 0.27	4.40 ± 0.29	3.80 ± 0.23	5.04 ± 0.31
Error (%)	MLE	7.23 ± 3.89	6.43 ± 4.56	9.10 ± 4.59	10.05 ± 5.15
	Drift	13.82 ± 5.13	15.13 ± 3.98	17.45 ± 4.15	19.56 ± 3.61
	Dens	7.41 ± 6.42	7.32 ± 3.46	4.42 ± 3.84	4.79 ± 3.54

Table S1: **Estimation of the potential well characteristics from simulations**, related to STAR Methods. Comparing the hybrid MLE algorithm with the Density and the Drift algorithms for an circular well with $a = b = 150$ nm, $E = 4$ (resp. 6) kT corresponds to $A = 0.02$ (resp. $A = 0.03$) μm^2/s.

	Algo	Δt=20 ms		Δt=50 ms	
		E=4 kT	E=6 kT	E=4 kT	E=6 kT
$\|\|\mu - \mu_{true}\|\|$	MLE	6 ± 3	3 ± 2	3 ± 2	3 ± 2
(nm)	Drift	19 ± 7	11 ± 5	15 ± 7	8 ± 4
	Dens	23 ± 5	16 ± 4	15 ± 3	10 ± 2
a (nm)	MLE	148 ± 6	139 ± 9	156 ± 7	145 ± 24
	Drift	124 ± 11	125 ± 11	129 ± 9	127 ± 11
	Dens	136 ± 20	128 ± 14	142 ± 22	129 ± 16
b (nm)	MLE	121 ± 6	113 ± 9	129 ± 8	124 ± 23
	Drift	104 ± 8	100 ± 8	108 ± 7	104 ± 10
	Dens	116 ± 17	111 ± 14	123 ± 20	112 ± 15
A (μm^2/s)	MLE	0.26 ± 0.02	0.34 ± 0.04	0.27 ± 0.02	0.34 ± 0.07
	Drift	0.12 ± 0.02	0.16 ± 0.03	0.08 ± 0.01	0.10 ± 0.02
	Dens	0.17 ± 0.02	0.23 ± 0.02	0.18 ± 0.02	0.25 ± 0.04
D (μm^2/s)	MLE	0.051 ± 0.002	0.052 ± 0.003	0.050 ± 0.003	0.049 ± 0.006
	Drift	0.040 ± 0.002	0.036 ± 0.001	0.030 ± 0.001	0.023 ± 0.001
	Dens	0.050 ± 0.002	0.051 ± 0.003	0.048 ± 0.004	0.051 ± 0.007
E (kT)	MLE	5.04 ± 0.30	6.48 ± 0.75	5.45 ± 0.33	6.92 ± 1.24
	Drift	3.00 ± 0.44	4.34 ± 0.64	2.83 ± 0.40	4.29 ± 0.76
	Dens	3.50 ± 0.22	4.54 ± 0.30	3.75 ± 0.23	4.82 ± 0.36
Error (%)	MLE	9.38 ± 3.48	7.14 ± 4.46	12.31 ± 4.53	11.38 ± 9.57
	Drift	14.21 ± 4.01	16.59 ± 3.73	20.12 ± 3.47	23.79 ± 3.87
	Dens	8.40 ± 5.45	9.62 ± 4.93	9.37 ± 6.79	9.89 ± 6.73

Table S2: **Estimation of the potential well characteristics from simulations**, related to STAR Methods. Comparing the hybrid MLE algorithm with the Density and the Drift algorithms for an elliptic well. The parameters are the same as for Table. S1 except that we use an elliptic boundary with $a = 150$ nm and $b = 100$ nm, $E = 4$ (resp. 6) kT corresponds to $A = 0.02$ (resp. $A = 0.03$) μm^2/s.

E (kT)	$\|\|\mu - \mu_{true}\|\|$ (nm)	a (nm)	b (nm)	A (μm^2/s)	D (μm^2/s)	E (kT)	Error (%)
1	21 ± 9	150 ± 23	145 ± 23	0.12 ± 0.01	0.047 ± 0.002	2.63 ± 0.11	29.49 ± 7.18
2	12 ± 7	144 ± 19	139 ± 19	0.15 ± 0.02	0.047 ± 0.003	3.06 ± 0.30	12.54 ± 6.88
3	7 ± 4	145 ± 24	140 ± 23	0.18 ± 0.03	0.049 ± 0.003	3.59 ± 0.60	9.27 ± 6.22
4	6 ± 4	148 ± 20	143 ± 19	0.22 ± 0.05	0.050 ± 0.003	4.31 ± 0.77	8.13 ± 5.15
5	4 ± 3	157 ± 17	151 ± 16	0.28 ± 0.05	0.050 ± 0.003	5.54 ± 0.90	7.01 ± 4.65
6	3 ± 2	163 ± 12	155 ± 11	0.34 ± 0.05	0.051 ± 0.003	6.69 ± 0.80	6.66 ± 4.15
7	3 ± 2	159 ± 14	152 ± 13	0.38 ± 0.06	0.051 ± 0.003	7.52 ± 1.11	6.48 ± 4.35
8	4 ± 14	157 ± 33	149 ± 24	0.41 ± 0.09	0.051 ± 0.004	8.00 ± 1.70	7.68 ± 8.65
9	3 ± 1	156 ± 18	149 ± 17	0.47 ± 0.10	0.051 ± 0.004	9.26 ± 1.80	7.32 ± 5.24
10	3 ± 1	155 ± 17	146 ± 17	0.51 ± 0.11	0.051 ± 0.004	9.97 ± 1.91	6.61 ± 5.86

Table S3: **Estimation of the potential well characteristics as a function of the energy**, related to STAR Methods. Based on a circular well ($a = b = 150$ nm) and using hybrid MLE algorithm and an acquisition time $\Delta t = 50$ ms.

Exp.	a (nm)	b (nm)	D (μm^2/s)	A (μm^2/s)	E (kT)	τ_e (ms)	n
Cav2.1 Δ47	143 ± 51	104 ± 33	0.091 ± 0.052	0.345 ± 0.209	3.9 ± 1.1	174 ± 134	1587
Cav2.1+47	145 ± 52	103 ± 33	0.087 ± 0.045	0.337 ± 0.189	3.9 ± 1.1	181 ± 141	1713
Cav2.1 endo	100 ± 47	73 ± 31	0.069 ± 0.051	0.224 ± 0.184	3.3 ± 1.2	94 ± 92	1878

Table S4: **Potential wells parameters extracted from CaV SPTs** related to Figure 2, n is the number of potential wells.

Exp.	Stability (s)	n
Cav2.1Δ47	56.2 ± 25.5	308
Cav2.1+47	57.4 ± 25.3	351
Cav2.1 endo	46.6 ± 15.6	284

Table S5: **CaV2.1 potential wells stability** related to Figures 2 and S5, n: number of families with as least two wells.

Exp.	a (nm)	b (nm)	D ($\mu m^2/s$)	A ($\mu m^2/s$)	E (kT)	τ_e (ms)	n
HEK293t	219 ± 71	155 ± 56	0.252 ± 0.136	0.787 ± 0.401	3.3 ± 0.9	101 ± 64	1057
COS-7 WT	230 ± 68	167 ± 45	0.461 ± 0.369	1.227 ± 0.984	2.7 ± 0.8	63 ± 46	859
COS-7 dATL	225 ± 70	163 ± 54	0.321 ± 0.292	0.868 ± 0.814	2.8 ± 0.9	106 ± 89	1250

Table S6: **Recovered potential wells parameters for ER datasets for HEK-293t, COS-7 WT and COS-7 dATL** related to Figure 3, n is the number of potential wells.

Algo.	# nodes	# links	# matched nodes	# matched links	Error (%)
DBSCAN	667 ± 22	687 ± 33	374 ± 4	336 ± 12	28.4 ± 2.2
DBSCANRec	638 ± 16	928 ± 38	391 ± 1	450 ± 8	8.1 ± 1.8

Table S7: **Estimation of the quality of the graph reconstruction algorithms**, related to Figures 5, 7, S7 and S8. Based on simulated trajectories and obtained from an optimised set of parameters (see Methods S1). The ground truth graph has 393 nodes and 550 links.
